# Supplementary material for: TBC1D5-Catalyzed Cycling of Rab7 Is Required for Retromer-Mediated Human Papillomavirus Trafficking during Virus Entry
Source: Cell Rep. Author manuscript; Available in PMC 2020 Jul 7. (PMC7339955; doi:10.1016/j.celrep.2020.107750)
Supplement: 1 [file NIHMS1603430-supplement-1.pdf]

Cell Reports, Volume *31*

Supplemental Information

TBC1D5-Catalyzed Cycling of Rab7 Is Required for Retromer-Mediated Human Papillomavirus Trafficking during Virus Entry

Jian Xie, Erin N. Heim, Mac Crite, and Daniel DiMaio

Figure S1

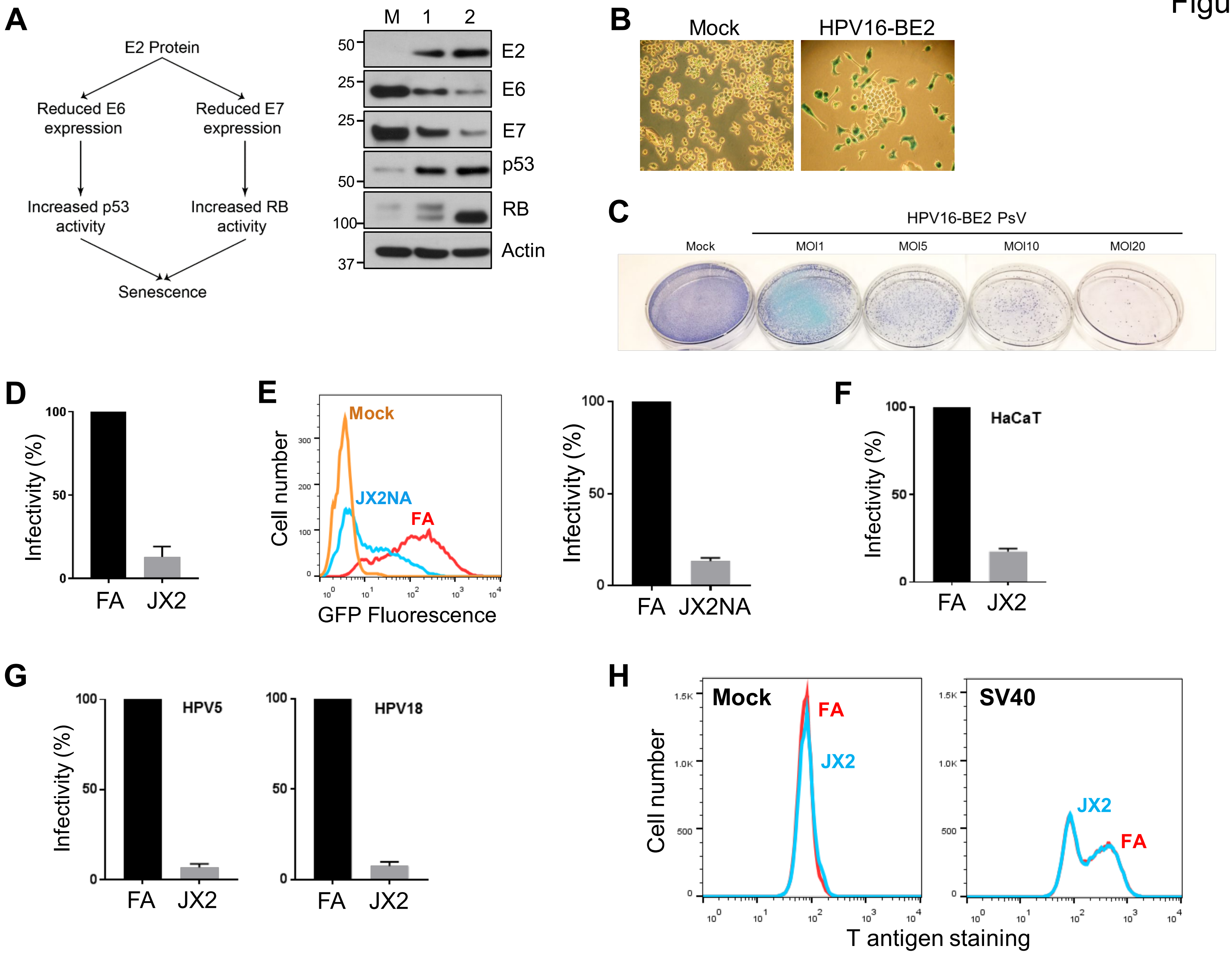

Related to Figure 1

Figure S1. Related to Figure 1. Isolation and validation of a traptamer that inhibits HPV infection. (A) Left panel. Schematic of biochemical pathway leading to senescence following E2 expression. Right panel. Extracts were prepared from mock-infected HeLa S3 cells (M) and HeLa S3 cells one and two days after infection with HPV16-BE2 at MOI of 20. Samples were subjected to SDS-PAGE and immunoblotted with antibodies recognizing BPV E2, HPV18 E6, HPV18 E7, p53, $p105^{Rb}$, and actin as loading control. **(B)** Photomicrographs of mock-infected HeLa S3 cells (left panel) and HeLa S3 cells 14 days after infection with HPV16-BE2 at MOI of 5 (right panel) stained for senescence-associated β-galactosidase. **(C)** HeLa S3 cells were mock-infected or infected with HPV16-BE2 at the indicated multiplicities of infection (MOI), plated at low cell density, incubated for two weeks, and stained with Giemsa. **(D)** Clonal HeLa-tTA cells expressing pT-FA or pT-JX2 in the absence of doxycycline were infected with HPV16-GFP PsV at MOI of 2, and the fraction of cells expressing GFP two days later was quantified by flow cytometry as a measure of infectivity. Graph shows mean and standard deviation of three independent experiments relative to cells expressing FA, which was set at 100%. **(E)** Clonal HeLa-tTA cells expressing pT-FA or pT-JX2NA, which lacks the APEX2 segment, were mock-infected or infected with HPV16-GFP PsV in the absence of doxycycline and analyzed as in **panel D**. Graph on left shows flow cytometry histograms. Averaged results of three independent experiments are shown at right. **(F)** HaCaT keratinocytes expressing vector control or JX2 were infected and analyzed as in **panel D**. **(G)** Clonal HeLa-tTA cells expressing pT-FA or pT-JX2 in the absence of doxycycline were infected with HPV5 and HPV18 PsV, as indicated, at MOI of 2 and analyzed as in **panel D. (H)** Clonal HeLa-tTA cells expressing pT-FA or pT-JX2 in the absence of doxycycline were mock-infected (left panel) or infected with SV40 at MOI of 1 (right panel). Two days later, cells were stained with antibody recognizing SV40 large T antigen and analyzed by flow cytometry.

Figure S2

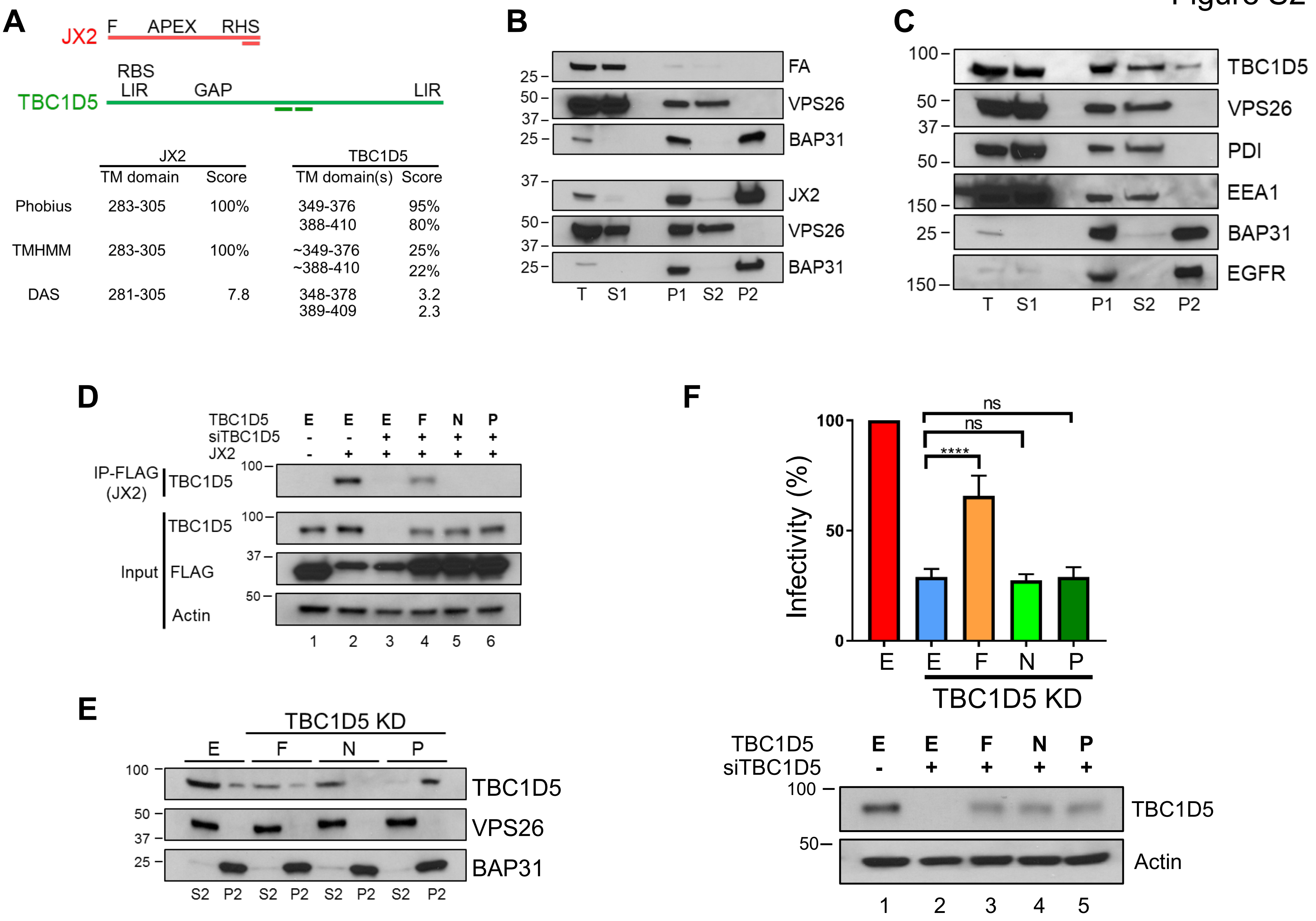

Related to Figures 1 and 4

Figure S2. Related to Figures 1 and 4. JX2 is a transmembrane protein. (A) Schematic diagram of JX2 (red) and TBC1D5 (green) with domains indicated: F, FLAG epitope; APEX, APEX2 segment; RHS, randomized hydrophobic segment; RBS, retromer binding site; GAP, GTPase activating segment; LIR, LC3 interacting region. Short lines represent putative TM domains. Table at bottom shows TM domains predicted by Phobius, TMHMM, or DAS prediction programs. The numbers in the TM domain columns give the boundaries of the predicted TM domain(s). The score indicates the probability that these sequences adopt a TM existence or the DAS score, where numbers higher than 2.2 reflect a high confidence of adopting a TM existence. **(B)** Clonal HeLa-tTA cells expressing pT-FA (top panels) or pT-JX2 (bottom panels) were osmotically swollen, mechanically sheared, fractionated into soluble and membrane fractions, and extracted with carbonate. Fractions were subjected to SDS-PAGE and immunoblotted for FLAG (recognizing FA and JX2), VPS26 (a peripheral membrane protein), and BAP31 (a TM protein). T, total extract; S1, supernatant containing soluble proteins; P1, total membrane pellet; S2, supernatant containing carbonate-extracted luminal and peripheral membrane proteins; P2, carbonate-resistant integral membrane protein pellet. **(C)** Lysates of uninfected HeLa S3 cells were prepared and processed as in **panel B**. Fractions were also immunoblotted for TBC1D5; the peripheral membrane protein, EEA1; the luminal protein, protein disulfide isomerase (PDI); and the TM protein, EGF receptor. **(D)** Clonal HeLa-tTA cells expressing JX2 (+) or FA (-) in the absence of doxycycline were transduced to express full-length TBC1D5 (F), the TBC1D5 null mutant (N) or the TBC1D5 PRTM mutant (P) or expressed only endogenous TBC1D5 (E). Cells were then transfected with scrambled control siRNA (-) or siRNA targeting the 3' untranslated region of the *TBC1D5* gene (+). Forty-eight hours later, extracts were prepared and expression of TBC1D5 was determined by immunoblotting (Input) or were analyzed by co-immunoprecipitation for JX2 binding as in Figure 3B. **(E)** Clonal HeLa-tTA cells expressing endogenous TBC1D5 or exogenous TBC1D5 and knocked down for endogenous expression as in **panel D** were subjected to carbonate extraction as in **panel B**. Only P2 and S2 fractions are shown. **(F)** Clonal HeLa-tTA cells as in **panel D** were infected 48 h after siRNA transfection with HPV16-GFP PsV at MOI of 2, and GFP fluorescence was measured 48 h.p.i. Infectivity was normalized to cells expressing endogenous TBC1D5. Bottom panels show immunoblot of TBC1D5 and actin expression.

Figure S3

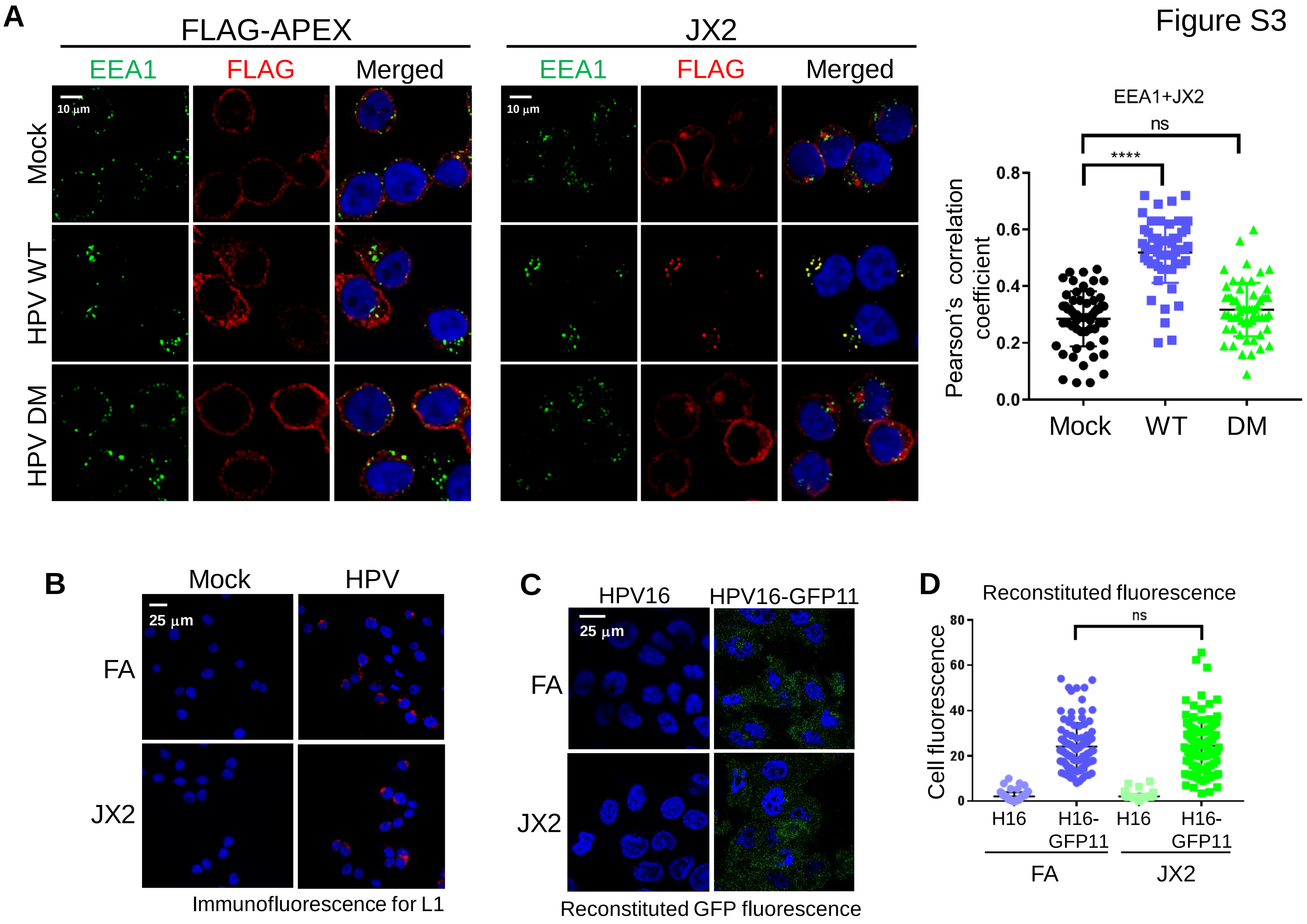

Related to Figures 2 and 5

Figure S3. Related to Figures 2 and 5. JX2 does not inhibit HPV internalization or cytoplasmic protrusion of L2. (A) HeLa-tTA cells expressing pT-FA or pT-JX2 in the absence of doxycycline were mock-infected or infected with wild-type HPV16-HcRed PsV at MOI of 150 or with DM HPV16-HcRed PsV containing the same number of encapsidated HcRed reporter plasmids. At 12 h.p.i., cells were stained with antibodies recognizing EEA1 (green) and FLAG-tagged JX2 (red) and visualized by confocal microscopy. Co-localization of EEA1 and JX2 is pseudocolored yellow in the Merged panels. A single confocal Z-plane is shown in each image. Graph shows Pearson's Coefficient for co-staining, with each dot representing an individual cell (at least 60 cells for each condition). ns, not significant; ****, $p<0.0001$. **(B)** Clonal HeLa-tTA cells expressing pT-FA or pT-JX2 in the absence of doxycycline were mock-infected or infected with HPV16 PsV at MOI of 50. At 8 h.p.i., cells were permeabilized and stained with antibody recognizing HPV16 L1 (red) and DAPI to stain nuclei (blue). **(C)** Clonal HaCaT-GFP1-10NES cells expressing pT-JX2 (bottom panels) or pT-FA (top panels) were infected at MOI of 2,000 with HPV16-HcRed PsV containing wild-type L2 (left panels) or L2 containing GFP11 fused to its C terminus (HPV16-GFP11) (right panels). Three h.p.i., cells were examined by confocal microscopy to detect reconstituted GFP fluorescence as a measure of cytoplasmic protrusion of L2. Fluorescence, green; nuclei, blue. **(D)** Fluorescence of cells as in **panel C** (at least 100 cells for each condition) was quantitated and shown as mean and standard deviation. H16, HPV16 PsV; H16-GFP11, HPV16-GFP11. Each dot represents an individual cell. ns, not significant.

Figure S4

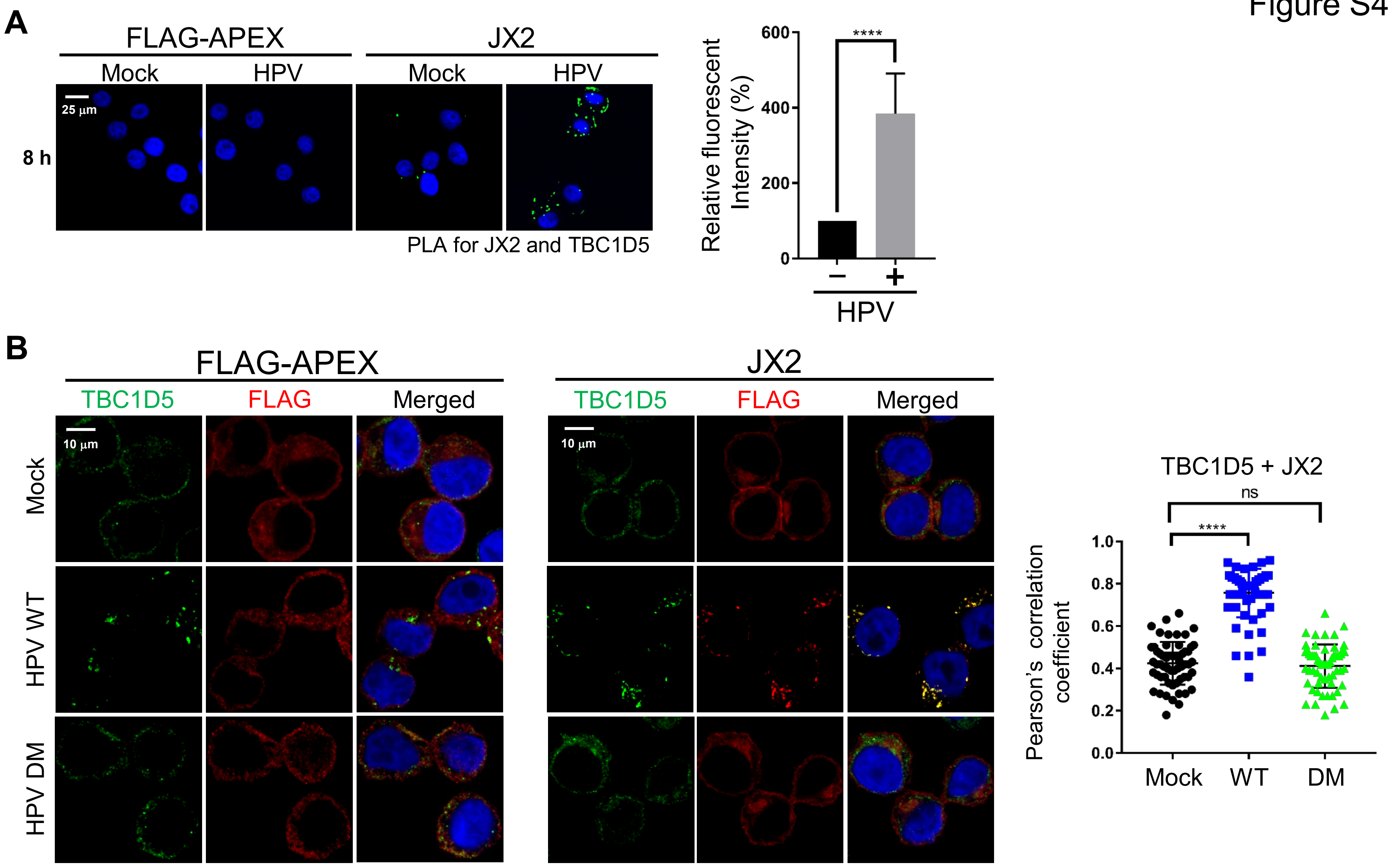

Related to Figures 2 and 5

Figure S4. Related to Figures 2 and 5. HPV infection induces co-localization of TBC1D5 and JX2. (A) Left panels. Clonal HeLa-tTA cells expressing pT-FA or pT-JX2 in the absence of doxycycline were mock-infected or infected with HPV16-HcRed PsV at MOI of 150. Eight h.p.i., cells were processed for PLA with antibodies recognizing TBC1D5 and FLAG-tagged JX2. PLA signal is green; nuclei are stained blue. Right panel. JX2-TBC1D5 PLA signal in at least 200 cells for each condition expressing JX2 with and without HPV16 infection was quantified. Results show mean signal and standard deviation normalized to mock-infected cells, which were set to 100%. ****, $p<0.0001$. **(B)** Cells as in **panel A** were mock-infected or infected with wild-type HPV16-HcRed PsV at MOI of 150 or DM mutant PsV containing the same number of encapsidated HcRed reporter plasmids. Twelve h.p.i., cells were stained with antibody recognizing TBC1D5 (green) or FLAG (red) and visualized by confocal microscopy. Overlap between TBC1D5 and FLAG is pseudocolored yellow in the Merged panels. Nuclei are stained blue. A single confocal Z-plane is shown in all images. Graph shows Pearson's correlation co-efficient of the overlap for at least 60 cells for each condition, displayed as in Figure 5B. ns, not significant; ****, $p<0.0001$.

Figure S5

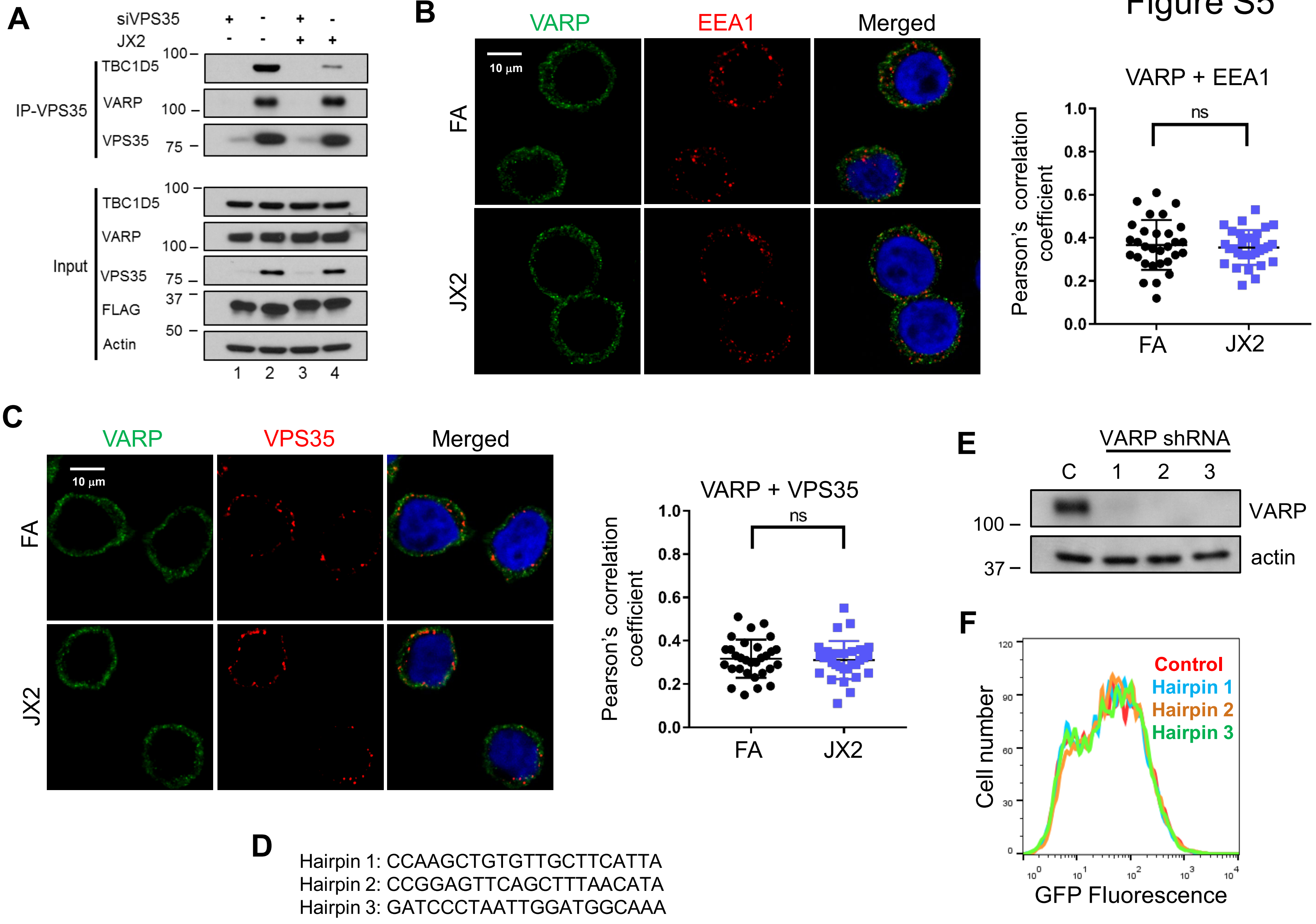

Related to Figures 3 and 4

Figure S5. Related to Figures 3 and 4. Lack of role of VARP in JX2 activity or HPV infection. (A) HeLa-tTA cells expressing pT-FA (-) or pT-JX2 (+) in the absence of doxycycline were transfected with control siRNA (-) or siRNA targeting VPS35 (+). Extracts were prepared two days after transfection and immunoprecipitated with anti-VPS35, electrophoresed, and immunoblotted for the indicated proteins. Input shows samples without immunoprecipitation. **(B and C)** HeLa-tTA cells expressing pT-FA or pT-JX2 in the absence of doxycycline were stained with antibody recognizing VARP (green) and either EEA1 (**panel B**) or VPS35 (**panel C**) (red). Co-localization is pseudocolored yellow in Merged panels, which is quantitated in the graphs. **(D)** Targeting sequences of shRNAs inhibiting VARP expression. **(E)** Western blot showing knockdown of VARP expression by the indicated shRNAs. C, controlled scrambled shRNA. **(F)** Flow cytometry histogram of cells expressing the indicated shRNAs and infected with HPV16-GFP PsV at MOI of 2. GFP fluorescence was measured 48 h.p.i. ns, not significant.

Figure S6

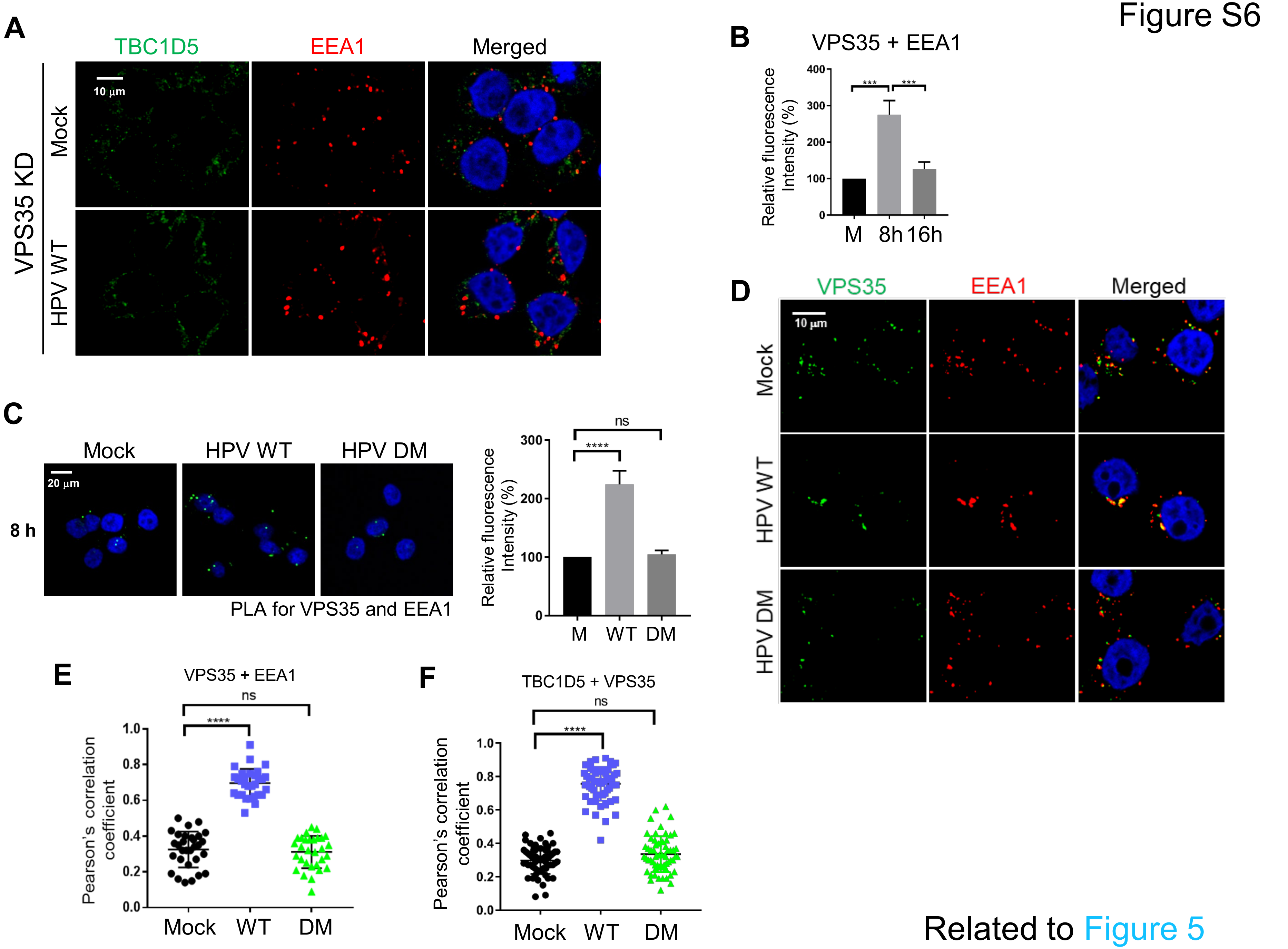

Related to Figure 5

Figure S6. Related to Figure 5. Effect of HPV infection on distribution of VPS35, EEA1, and TBC1D5. (A) See legend to Figure 5A. **(B)** VPS35 and EEA1 PLA signal as in Figure 5C (left panels) at 8 and 16 h.p.i were quantified as described in the legend to Figure 2B. ***, $p<0.001$. **(C)** HeLa S3 cells were mock-infected or infected with wild-type HPV16-HcRed PsV at MOI of 150 or DM mutant PsV containing the same number of encapsidated HcRed reporter plasmids. At 8 h.p.i., cells were subjected to PLA with antibodies recognizing VPS35 and EEA1. PLA signal is green, and nuclei are blue. VPS35-EEA1 PLA signal was quantified and displayed as described in legend to Fig. 2B. **(D)** HeLa S3 cells were infected as in **panel C**. At 12 h.p.i., cells were stained with antibodies recognizing VPS35 (green) and EEA1 (red) and visualized by confocal microscopy. Overlap between VPS35 and EEA1 is pseudocolored yellow in Merged panel. Nuclei are stained blue. **(E)** VPS35-EEA1 overlap in images as in **panel D** was quantified as described in Figure 5B. **(F)** TBC1D5-VPS35 overlap in images as in Figure 5D was quantified as described in Figure 5B, for at least 60 cells for each condition. ns, not significant; ****, $p<0.0001$.

Figure S7

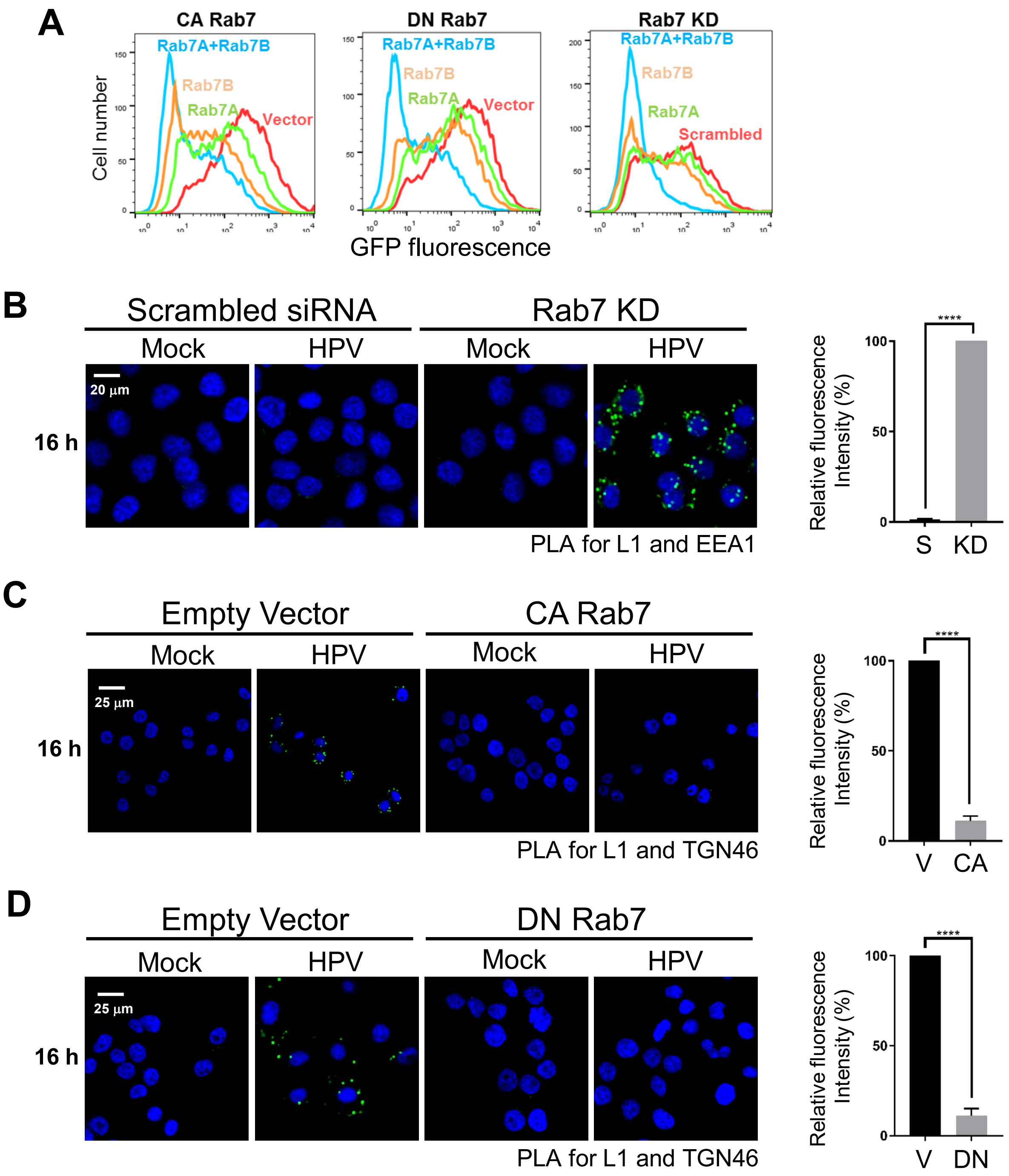

Related to Figure 6

Figure S7. Related to Figure 6. Rab7 cycling is required for HPV infection and transit to the trans-Golgi network. (A) HeLa-tTA cells as in Figure 6B were infected with HPV16-GFP PsV at MOI of 2. Two days after infection, GFP fluorescence was measured by flow cytometry. Graphs show representative flow cytometry histograms. **(B)** Left panels. HeLa S3 cells were transfected with control scrambled siRNA or siRNAs targeting Rab7A and Rab7B. Two days later, cells were mock-infected or infected with HPV16-HcRed PsV at MOI of 150. Sixteen h.p.i., cells were subjected to PLA with antibodies recognizing HPV L1 and EEA1. PLA signal is green; nuclei are stained blue. Right panel. L1-EEA1 PLA signal in at least 200 cells for each HPV-infected condition was quantified and displayed as described in legend to Figure 2B. S, control scrambled siRNA; KD, Rab7A and Rab7B siRNA. ****, $p<0.0001$. **(C)** Left panels. Clonal HeLa-tTA cells expressing pT_{puro} and pT_{hygro} (Empty Vector) or expressing CA Rab7A plus Rab7B in the absence of doxycycline were mock-infected or infected with HPV16-HcRed PsV at MOI of 150. Sixteen h.p.i., cells were subjected to PLA with antibodies recognizing HPV L1 and TGN46. PLA signal is green; nuclei are stained blue. Right panel. L1-TGN46 PLA signal in at least 200 cells for each HPV-infected condition was quantified and displayed as described in legend to Figure 2B. V, empty vector; CA, constitutively-active Rab7A and Rab7B. ****, $p<0.0001$. **(D)** Left panels. As in **panel C**, except cells expressed DN Rab7A plus Rab7B instead of CA Rab7. Right panel. L1-TGN46 PLA signal in at least 200 cells for each HPV-infected condition was quantified and displayed as described in legend to Figure 2B. V, empty vector; DN, dominant-negative Rab7A and Rab7B. ****, $p<0.0001$.

Table S1. Related to Figures 4, 6, 7, and S7. Summary of the Effect of TBC1D5 and Rab7 on Retromer-mediated Cargo Trafficking

	infectivity HPV	endosome entry		endosome accumulation		TGN entry		retromer association	retromer dissociation
		HPV	cell cargo	HPV	cell cargo	HPV	cell cargo	HPV	HPV
wild-type cells	+	+	+	-	-	+	+	+	+
TBC1D5 knockdown	-	+	+	+	-	-	+	+	-
CA Rab7	-	+	+	+	-	-	+	+	-
DN Rab7	-	+	+	+	+	-	-	-	n.a.

n.a. – not applicable

Table S2. Related to STAR METHODS. Oligonucleotides for Library Construction and Recovery

FWD long	GCCTGCTAGGGATCCGGCGGC N5B N5B N5B N5B N5B N5B N5B N5B N5B N5B N5B N5B N5B N5B N5B N5B N5B N5B N5B N5B N5B N5B N5B N5B N5B N5B N5B N5B N5B N5B N5B TAG TAG CCT GAT GAG GAA<br><br>N is A:G:C:T = 1:1:1:1<br><br>5 is A:G:C:T = 1:1:1:7<br><br>B is A:G:C:T = 0:1:1:1
REV Long	GAATTCTCCCTACACTGCTCACTCATCAGGCTACTA
FWD short	GGCTCTTTTGTCTGACCCTGTATTCCGCCC
REV short	GACACT GAATTC TCC CTA CAC TGC TCA CTC

Table S3. Related to STAR METHODS. Antibodies Used for Proximity Ligation Assays

Antibody 1 (Mouse)		Antibody 2 (Rabbit)	
anti-HPV16 L1	BD Biosciences 554171; 1:75[a]	anti-EEA1<br>anti-TGN46<br>anti-VSP35	Cell Signaling Technologies 2411; 1:50<br>Abcam ab50595; 1:150<br>Abcam ab157220; 1:75
anti-FLAG (recognizes JX2)	Sigma F3165; 1:200	anti-TBC1D5	Abcam ab203896; 1:50
EEA1	BD Biosciences 610457; 1:75	anti-VPS35	as above

[a] Dilutions used are indicated for all antibodies.
