# Supplementary material for: TBC1D5-Catalyzed Cycling of Rab7 Is Required for Retromer-Mediated Human Papillomavirus Trafficking during Virus Entry
Source: Cell Rep. Author manuscript; Available in PMC 2020 Jul 7. (PMC7339955; doi:10.1016/j.celrep.2020.107750)
Supplement: 2 [file NIHMS1603430-supplement-2.pdf]

Cell Reports

Article

TBC1D5-Catalyzed Cycling of Rab7 Is Required for Retromer-Mediated Human Papillomavirus Trafficking during Virus Entry

Graphical Abstract

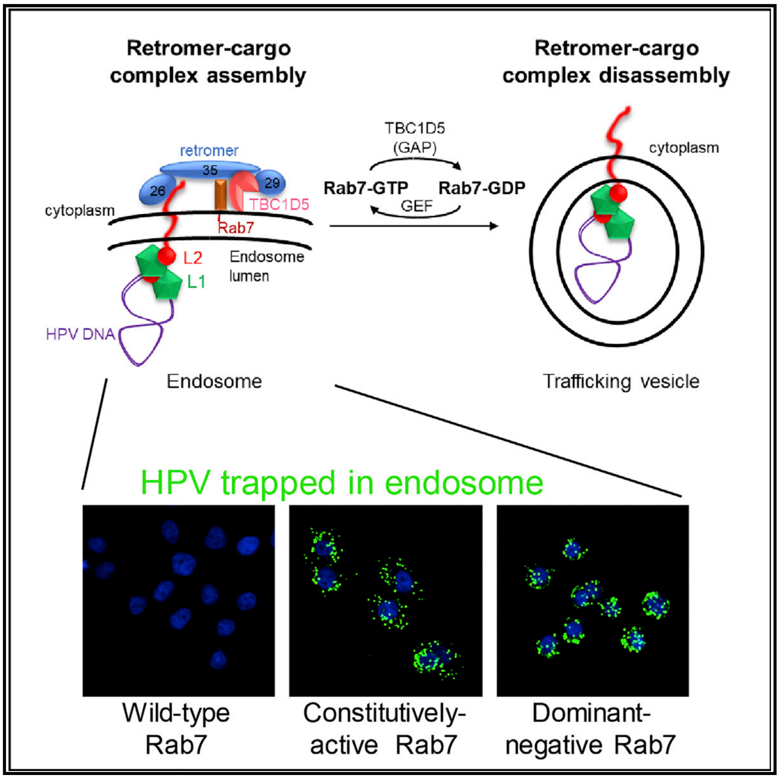

Highlights

- Traptamer screening identifies TBC1D5 as a human papillomavirus entry factor
- TBC1D5 is a Rab7 GTPase-activating protein that activates retromer during HPV entry
- Rab7 cycling between GTP- and GDP-bound forms is required for HPV entry
- HPV trafficking displays different Rab7 requirements than cellular cargo

Authors

Jian Xie, Erin N. Heim, Mac Crite, Daniel DiMaio

Correspondence

daniel.dimaio@yale.edu

In Brief

Xie et al. designed a protein interference screen that identified TBC1D5 as a cellular protein required for HPV entry. TBC1D5 stimulates the GTPase activity of Rab7, which is required for retromer to deliver HPV to the retrograde transport pathway for trafficking of incoming HPV to the nucleus.

Xie et al., 2020, Cell Reports *31*, 107750
June 9, 2020 © 2020 The Author(s).
https://doi.org/10.1016/j.celrep.2020.107750

Cell Reports

CellPress
OPEN ACCESS

Article

TBC1D5-Catalyzed Cycling of Rab7 Is Required for Retromer-Mediated Human Papillomavirus Trafficking during Virus Entry

Jian Xie,[1] Erin N. Heim,[1] Mac Crite,[2] and Daniel DiMaio[1,3,4,5,6,*]

[1]Department of Genetics, Yale School of Medicine, PO Box 208005, New Haven, CT 06520-8005, USA
[2]Department of Microbial Pathogenesis, Yale School of Medicine, 295 Congress Avenue, New Haven, CT 06519, USA
[3]Department of Therapeutic Radiology, Yale School of Medicine, PO Box 208040, New Haven, CT 06520-8040, USA
[4]Department of Molecular Biophysics & Biochemistry, Yale School of Medicine, PO Box 208024, New Haven, CT 06520-8024, USA
[5]Yale Cancer Center, PO Box 208028, New Haven, CT 06520-8028, USA
[6]Lead Contact
*Correspondence: daniel.dimaio@yale.edu
https://doi.org/10.1016/j.celrep.2020.107750

SUMMARY

During virus entry, human papillomaviruses are sorted by the cellular trafficking complex, called retromer, into the retrograde transport pathway to traffic from the endosome to downstream cellular compartments, but regulation of retromer activity during HPV entry is poorly understood. Here we selected artificial proteins that modulate cellular proteins required for HPV infection and discovered that entry requires TBC1D5, a retromer-associated, Rab7-specific GTPase-activating protein. Binding of retromer to the HPV L2 capsid protein recruits TBC1D5 to retromer at the endosome membrane, which then stimulates hydrolysis of Rab7-GTP to drive retromer disassembly from HPV and delivery of HPV to the retrograde pathway. Although the cellular retromer cargos CIMPR and DMT1-II require only GTP-bound Rab7 for trafficking, HPV trafficking requires cycling between GTP- and GDP-bound Rab7. Thus, ongoing cargo-induced membrane recruitment, assembly, and disassembly of retromer complexes drive HPV trafficking.

INTRODUCTION

Human papillomaviruses (HPVs) are non-enveloped DNA viruses that play an etiologic role in ~5% of human cancers. During virus entry, incoming HPV virions remain in membranous vesicles after endocytosis until they reach the nucleus, where viral DNA replication occurs (Schelhaas et al., 2012; Day et al., 2013; DiGiuseppe et al., 2016; Lipovsky et al., 2013; Siddiqa et al., 2018a, 2018b; Aydin et al., 2017; Day et al., 2019). Although internalized HPV is in the endosome lumen, the L2 minor capsid protein binds directly to cellular proteins confined to the cytoplasm (Bergant Marušič et al., 2012; Popa et al., 2015). The L2 protein contains a cationic cell-penetrating peptide (CPP) that drives protrusion of the C terminus of L2 through the endosomal membrane into the cytoplasm to bind retromer (Zhang et al., 2018), a cytoplasmic coat protein complex consisting of three subunits (VPS26, VPS29, and VPS35) that regulates cellular protein trafficking (Burd and Cullen, 2014). Retromer bound to L2 sorts the incoming HPV virion into the vesicular retrograde pathway for transport to the *trans*-Golgi network (TGN). The major retromer binding site on HPV16 L2 resembles a retromer binding motif present in some cellular retromer cargos, including divalent metal transporter 1 isoform II (DMT1-II) and cation-independent mannose phosphate receptor (CIMPR) (Popa et al., 2015). In DMT1-II, this motif binds directly to retromer subunit VPS26 (Lucas et al., 2016). After trafficking to the TGN, HPV remains in retrograde transport compartments until it enters the nucleus (DiGiuseppe et al., 2016; Aydin et al., 2017; Day et al., 2019).

HPV trafficking also requires Rab7B (Young et al., 2019; Day et al., 2013; Lipovsky et al., 2013). The Rab proteins are small guanosine triphosphatases (GTPases) that regulate intracellular membrane trafficking events by cycling between a membrane-associated guanosine triphosphate (GTP)-bound form and a cytosolic guanosine diphosphate (GDP)-bound form (Guerra and Bucci, 2016; Stroupe, 2018). GTP-bound G proteins recruit coat protein complexes such as clathrin and COPI to membranes, and their cognate GTPase-activating proteins (GAPs) promote hydrolysis of bound GTP to trigger release of the coats from membranes, allowing trafficking to proceed (Barlowe et al., 1994; Eugster et al., 2000; Harrison et al., 2014; Orcl et al., 1993; Palmer et al., 1993; Szafer et al., 2001; Yoshihisa et al., 1993). It is not known whether Rab7B plays a direct role in retromer-mediated delivery of HPV into the retrograde pathway or whether it regulates trafficking of a cellular protein required for HPV entry.

Retromer is structurally distinct from the other coat protein complexes (Dell'Angelica and Bonifacino, 2019), and the molecular mechanisms that regulate assembly of retromer and the cargo at the endosomal membrane and disassembly of retromer from the cargo remain elusive. Furthermore, other than HPV, all

This is an open access article under the CC BY license (http://creativecommons.org/licenses/by/4.0/).

known retromer cargos are cellular transmembrane (TM) proteins, so mechanistic study of HPV entry will likely reveal new aspects of retromer action and its regulation. Binding of cellular cargos to retromer recruits retromer to the endosomal membrane in association with Rab7-GTP and SNX proteins, whereas GDP-trapped Rab7 causes retromer dissociation from the endosome membrane (Harrison et al., 2014, 2016; Jimenez-Orgaz et al., 2018; Lucas et al., 2016; Rojas et al., 2008; Seaman et al., 2009; Vardarajan et al., 2012; Liu et al., 2012; Priya et al., 2015; Harbour et al., 2010). After budding from the endosome and retromer dissociation, vesicles containing cargo traffic to the TGN, where the cargo is delivered by membrane fusion. TBC1D5 is a retromer-associated GAP that stimulates the GTPase activity of Rab7. TBC1D5 overexpression induces dissociation of retromer from membranes (Borg Distefano et al., 2018; Jia et al., 2016; Jimenez-Orgaz et al., 2018; Seaman et al., 2009). Thus, by analogy to other GAPs that stimulate membrane dissociation of coat protein complexes, TBC1D5 may catalyze disassembly of the retromer-HPV complex at the endosome membrane to coordinate retrograde trafficking.

To identify additional cellular proteins that regulate HPV trafficking, we developed a protein interference screen that employs a retroviral library expressing many artificial proteins with a randomized hydrophobic segment that can serve as a TM domain. We then use biological selection to isolate cells with the desired phenotype because of the ability of an artificial protein to bind a cellular protein and modulate its activity (Freeman-Cook and DiMaio, 2005). We named these artificial proteins ‘‘traptamers’’ for TM protein aptamers. We have isolated traptamers that specifically modulate the activity of several different TM proteins (e.g., Cammett et al., 2010).

Here we screened a library for traptamers that inhibit HPV infection. Bovine papillomavirus (BPV) encodes the E2 transcription factor, which represses transcription of the HPV E6 and E7 oncogenes in cervical cancer cells and imposes senescent growth arrest (Goodwin and DiMaio, 2000; Goodwin et al., 2000). Traptamers that block infection by an HPV16 pseudovirus (PsV) expressing BPV E2 allow continued growth of HeLa cells. We used this screen to isolate a traptamer, JX2, that inhibits HPV endosomal exit by inhibiting the action of TBC1D5, the Rab7 GAP. Further analysis showed that TBC1D5 itself is essential for HPV entry and is recruited to retromer and the endosome by binding of HPV to retromer. Importantly, TBC1D5-catalyzed cycling of Rab7 is critical for disassembly of the retromer-HPV complex and sorting of HPV into the retrograde pathway. These results reveal the mechanism by which a non-enveloped virus engages the cellular trafficking machinery to establish infection and provide insight into mechanisms that regulate retromer activity.

RESULTS

Isolation of Traptamers that Inhibit HPV Infection

We designed a genetic screen to isolate traptamers that inhibit infection by HPV16 PsV (Figure 1A). We inserted the BPV E2 gene into a HPV16 PsV and generated HPV16-BE2 PsV stocks in 293TT cells. Infection of HeLa S3 cells with HPV16-BE2 repressed HPV18 E6 and E7 expression, activated the p53 and $p105^{Rb}$ tumor suppressors, and caused most of the cells to undergo senescence (Figures S1A–S1C).

We constructed a library (designated EHFA) encoding traptamers consisting of an N-terminal FLAG epitope tag, the APEX2 ascorbic peroxidase, and a 26-residue segment comprised of ~80% hydrophobic amino acids in random order to serve as a TM domain (Figure 1B). The FLAG tag and the APEX2 segment were included to allow immunoprecipitation of the traptamers and biotinylation of traptamer-associated proteins, respectively. DNA encoding these elements was cloned into the $MSCV_{puro}$ vector and transformed into bacteria. Plasmid DNA from ~250,000 pooled bacterial colonies was packaged into retrovirus particle cells to generate the EHFA library. For a control, we constructed MSCVpFA encoding the FLAG-APEX2 segment (FA) without the randomized hydrophobic sequence.

We infected HeLa S3 cells with the EHFA traptamer library at a low multiplicity of infection (MOI) to express one or a few traptamers in each cell. Transduced cells were infected twice with HPV16-BE2 PsV at an MOI of 20. The vast majority of cells senesced, but 2 weeks after infection, rare proliferating colonies were picked and expanded. PCR with primers that annealed to fixed sequences flanking the randomized segment of the traptamer genes was used to recover the gene encoding a traptamer designated JX2 from DNA isolated from proliferating cells. Here we describe our analysis of JX2 and identification of TBC1D5 as an HPV entry factor. Other inhibitory traptamers will be reported separately.

Validation of Traptamers that Inhibit HPV Entry

To confirm the activity of JX2, we cloned the JX2 gene into the Retro-X Tet-off inducible expression system, pTight (pT), which allows doxycycline-regulated gene expression in cells expressing the tetracycline transcriptional factor, tTA. Multiple clonal HeLa-tTA cells expressing JX2 in the absence of doxycycline showed significant resistance to HPV16 infection compared with cells expressing FA lacking a randomized hydrophobic segment (Figures 1C and S1D).

As expected, the randomized segment of JX2 was highly hydrophobic (23 of 26 residues were valine, leucine, isoleucine, phenylalanine, or methionine) and displayed no significant sequence similarity to any known protein (Figure 1B). Multiple TM domain prediction programs predicted the hydrophobic segment of JX2 to form a TM domain (Figure S2A). To test whether JX2 was stably inserted into cell membranes, we isolated crude membranes from mechanically disrupted cells and performed carbonate extraction at high pH, which extracts proteins peripherally associated with membranes (Fujiki et al., 1982). As expected, the *bona fide* TM proteins BAP31 and epidermal growth factor (EGF) receptor were exclusively in the crude membrane fraction, P1, resistant to carbonate extraction, and present in the final membrane pellet fraction, P2 (Figures S2B and S2C). In contrast, the peripheral membrane proteins VPS26 and EEA1 and the luminal protein PDI (protein disulfide isomerase) were mostly in the soluble S1 fraction, and the portion in the P1 fraction was extracted by carbonate into the soluble S2 fraction. Like the TM protein controls, JX2 was in the P1 membrane fraction and resistant to carbonate extraction (Figure S2B), strongly suggesting that it is a TM protein. In

CellPress
OPEN ACCESS

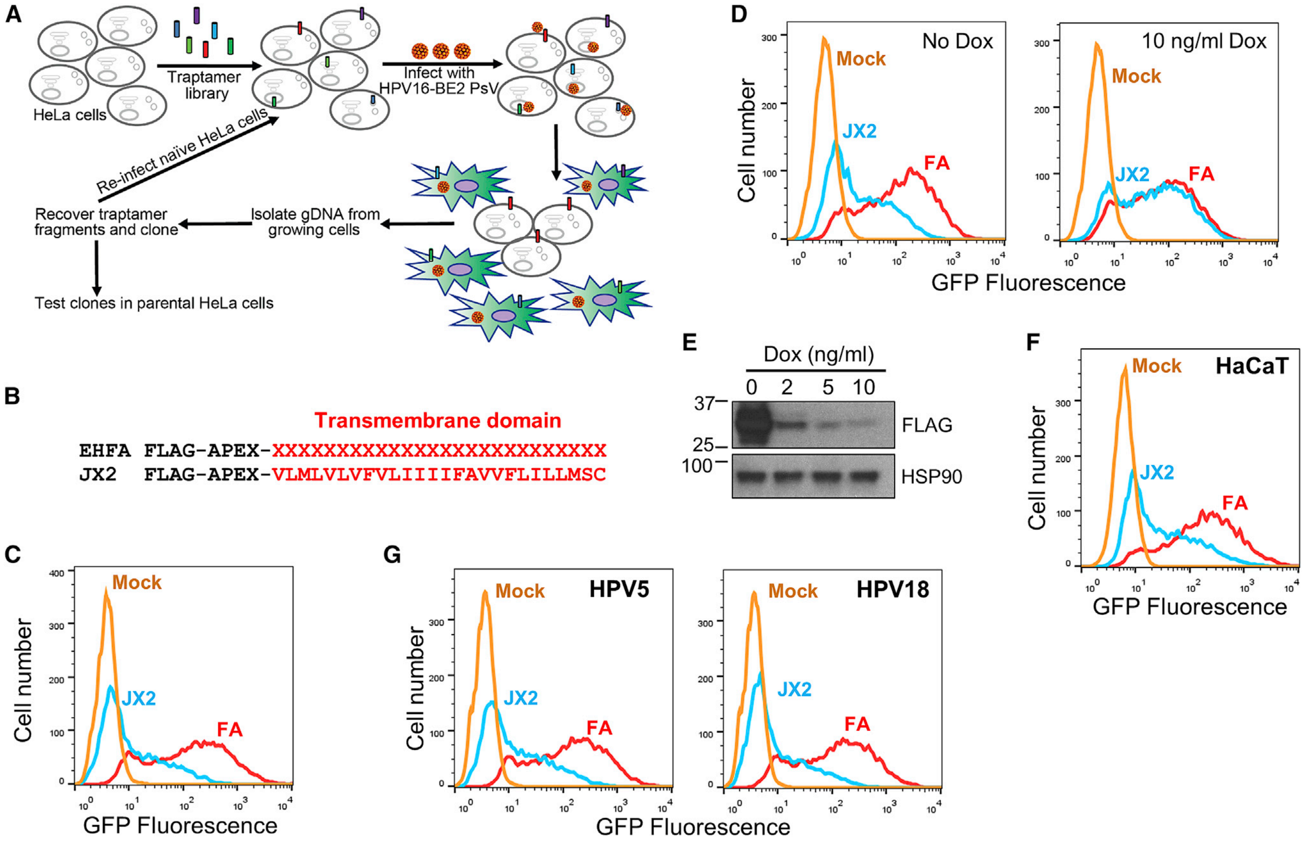

Figure 1. Senescence-Based Traptamer Screen for Inhibitors of HPV Infection

(A) Scheme to isolate traptamers that inhibit HPV infection. See text for details.

(B) Traptamer sequences. In the EHFA library, FLAG is the epitope tag, and APEX is the APEX2 ascorbate peroxidase segment. Randomized hydrophobic positions are indicated by a red X. The bottom line shows the active traptamer JX2, with the sequence of the hydrophobic TM domain shown in red in single-letter amino acid representation.

(C) Clonal HeLa-tTA cells stably expressing empty vector FA (red) or JX2 (blue) in the absence of doxycycline were mock-infected (mock, orange) or infected with HPV16 PsV containing the GFP reporter plasmid (HPV16-GFP) at an MOI of 2. Two days later, GFP fluorescence was measured by flow cytometry to assess infectivity. The graph shows flow cytometry histograms. Similar results were obtained and quantified in three independent JX2 cell lines (Figure S1D).

(D) Clonal HeLa-tTA cells expressing FA or JX2 were treated with 10 ng/ml doxycycline for 2 days to repress JX2 (right panel) or left untreated (left panel). The cells were then infected with HPV16-GFP PsV at an MOI of 2 and maintained in the presence or absence of doxycycline. Two days after infection, GFP fluorescence was measured as in (C).

(E) JX2 expression in HeLa-tTA/pT-JX2 cells incubated at the indicated concentration of doxycycline was monitored by SDS-PAGE and immunoblotting with anti-FLAG antibody.

(F) HaCaT keratinocytes stably expressing FA or JX2 were infected with HPV16-GFP PsV at an MOI of 2. Two days later, GFP fluorescence was measured as in (C). Similar results were obtained and quantified in three independent experiments (Figure S2F).

(G) Cells as in (C) were infected with HPV5-GFP or HPV18-GFP PsV at an MOI of 2. Two days later, GFP fluorescence was measured as in (C). Similar results were obtained and quantified in three independent experiments (Figure S1G).

See also Figures S1 and S2.

contrast, FA was present in the soluble S1 fraction only, indicating that the TM character of JX2 was due to its hydrophobic segment.

Immunofluorescence showed that JX2 and FA were widely distributed in the cell, with nuclear exclusion and minimal overlap with the endosome marker EEA1 (Figure S3A). HPV infection caused a marked redistribution of JX2 and EEA1 to discrete punctate structures with substantial overlap, whereas the distribution of FA did not change upon infection. Furthermore, JX2 and EEA1 redistribution did not occur when cells were infected with an HPV L2 mutant lacking the retromer binding sites (double mutant [DM mutant]). Thus, HPV-induced redistribution of JX2 to the endosome required the TM domain of JX2 and the retromer binding site on L2.

Doxycycline repressed expression of JX2 and restored sensitivity to HPV infection, showing that resistance to HPV16 was caused by JX2 expression (Figures 1D and 1E). In addition, the APEX2 segment of JX2 was not required for inhibitory activity (Figure S1E). In most of the experiments described below, we used clonal HeLa-tTA cells expressing JX2 from pT-JX2 in the absence of doxycycline. As a control, we used cells expressing the pT vector encoding FA without a TM domain (pT-FA).

JX2 also inhibited HPV16 PsV infection in human HaCaT skin keratinocytes (Figures 1F and S1F). HeLa-tTA cells expressing JX2 were also resistant to infection by HPV18 or HPV5 PsV, HPV types that infect the genital mucosa, like HPV16, and skin, respectively (Figures 1G and S1G), but JX2 did not inhibit infection by SV40 (Figure S1H), a non-enveloped DNA virus that undergoes retromer-independent entry. Thus, JX2 specifically inhibits several pathogenic HPV types in two epithelial cell lines commonly used to study HPV infection.

JX2 Causes Accumulation of Incoming HPV in the Endosome without Blocking L2 Protrusion

We next determined the step of HPV infection that was blocked by JX2. HeLa-tTA cells expressing JX2 or FA were infected with HPV16 PsV at an MOI of 50. Eight hours later, cells were permeabilized and stained with an antibody recognizing the L1 protein. As shown in Figure S3B, control cells expressing FA and cells expressing JX2 showed similar L1 staining, indicating that JX2 does not interfere with virus binding or internalization.

To examine HPV trafficking in cells expressing JX2, we performed proximity ligation assays (PLAs), in which localization of an incoming virus is determined with antibodies that recognize a viral protein and a cellular protein confined to a particular cell compartment (Lipovsky et al., 2015). In PLAs, a fluorescent signal is generated only when the two proteins are in the same compartment in close proximity. Cells expressing JX2 or FA were mock-infected or infected with HPV16 PsV at an MOI of 150, and PLA was performed with antibodies recognizing HPV16 L1 and the endosomal marker EEA1 (Figures 2A and 2B). There was no PLA signal in uninfected cells. At 8 h post-infection (h.p.i.), control and JX2 cells displayed similar PLA signals, confirming that JX2 did not inhibit virus internalization or endosome arrival. At 16 h.p.i., there was little PLA signal in control-infected cells because incoming HPV had departed from the endosome, as reported previously (Zhang et al., 2014). In striking contrast, there was more L1-EEA1 PLA signal in JX2 cells at 16 h.p.i. than at 8 h.p.i., showing that JX2 impaired exit of HPV from the endosome. Consistent with this result, at 16 h.p.i., JX2 reduced the PLA signal for L1 and TGN46, a TGN marker, compared with control cells expressing FA (Figures 2C and 2D). These results show that JX2 inhibits HPV16 infection by blocking endosome exit and preventing trafficking to the TGN.

Because mutations in the L2 CPP that prevent protrusion of the L2 C terminus into the cytoplasm also cause endosome accumulation (Zhang et al., 2018), we used a split GFP assay in HaCaT cells to test whether JX2 affected L2 protrusion. In this assay, fluorescence is reconstituted only when a short segment of GFP fused to the C terminus of L2 protrudes through the endosomal membrane and associates with the rest of GFP expressed in the cytoplasm (Zhang et al., 2018). As shown in Figures S3C and S3D, JX2 did not affect the level of reconstituted fluorescence. Thus, JX2 does not inhibit protrusion of the L2 protein into the cytoplasm.

JX2 Inhibits Disassembly of the Retromer-HPV Complex and Causes Accumulation of GTP-Bound Rab7

Because manipulations that impair binding of L2 to retromer cause HPV to accumulate in the endosome (Popa et al., 2015; Zhang et al., 2018), we hypothesized that JX2 interfered with binding of HPV to retromer. To test this, we performed PLA for L1 and the retromer subunit VPS35. As reported previously (Popa et al., 2015), in cells lacking JX2, there was readily detectable L1-VPS35 PLA signal at 8 h.p.i. but not at 16 h.p.i. (Figures 2E and 2F). Similarly, a PLA signal was detectable in cells expressing JX2 at 8 h.p.i. In contrast, at 16 h.p.i., cells expressing JX2 displayed an increased L1-VPS35 PLA signal compared with control cells. Thus, rather than inhibiting association between retromer and L2, JX2 appears to arrest the L2-retromer complex in a non-productive state.

Rab7B is required for HPV trafficking (Day et al., 2013; Lipovsky et al., 2013), but the biochemical role of Rab7 in HPV entry is not known. Because expression of dominant-negative (i.e., GDP-trapped) Rab7 causes dissociation of retromer from the endosomal membrane, we hypothesized that increased Rab7-GTP would inhibit disassembly of the retromer-HPV complex and block HPV endosome exit, the phenotype caused by JX2. To test whether JX2 affected the level of Rab7-GTP, we performed pull-downs with the Rab7 effector Rab-interacting lysosomal protein (RILP), which binds to GTP-bound but not GDP-bound Rab7 (Sun et al., 2009). Purified glutathione S-transferase (GST) and a GST-RILP fusion protein were incubated with extracts of control HeLa cells and cells expressing JX2. Cellular proteins bound to GST or GST-RILP were pulled down with glutathione beads, and GTP-bound Rab7 in the pellet was identified by SDS-polyacrylamide gel electrophoresis (PAGE) and immunoblotting. As shown in Figure 3A, lane 2, a basal level of Rab7-GTP is present in control cells expressing FA. Notably, the level of Rab7-GTP was markedly increased in cells expressing JX2, although the total level of Rab7 was not affected by JX2 (Figure 3A, lane 3). JX2 also caused accumulation of Rab7-GTP in HPV-infected cells (data not shown).

JX2 Is in a Stable Complex with the Rab7 GAP, TBC1D5

We next considered how JX2 increased Rab7-GTP levels. The retromer subunits VPS29 and VPS35 associate directly with the GAP TBC1D5, which stimulates conversion of Rab7-GTP to Rab7-GDP (Borg Distefano et al., 2018; Jia et al., 2016; Jimenez-Orgaz et al., 2018; Seaman et al., 2009, 2018; Harbour et al., 2010). RILP pull-down experiments confirmed that TBC1D5 knockdown increased the level of Rab7-GTP, similar to the level in cells expressing JX2 (Figure 3A, lane 4).

To test whether JX2 was in a complex with TBC1D5, we prepared detergent extracts from uninfected HeLa-tTA cells expressing JX2 or FA. There was no difference in the total amount of TBC1D5 in cells, regardless of whether they express JX2 (Figure 3B, input, lanes 1 and 4). We then immunoprecipitated JX2 with an anti-FLAG antibody, subjected the immunoprecipitate to SDS-PAGE, and probed with an antibody that recognizes TBC1D5. As shown in the top panel of Figure 3B, lanes 1 and 4, anti-FLAG co-immunoprecipitated TBC1D5 from cells expressing JX2 but not from control cells expressing FA. These results indicate that JX2 is in a stable complex with TBC1D5 and that complex formation requires the TM domain of JX2. TBC1D5 co-immunoprecipitation was not significantly affected by knockdown of the retromer subunit VPS35 (Figure 3B, lane

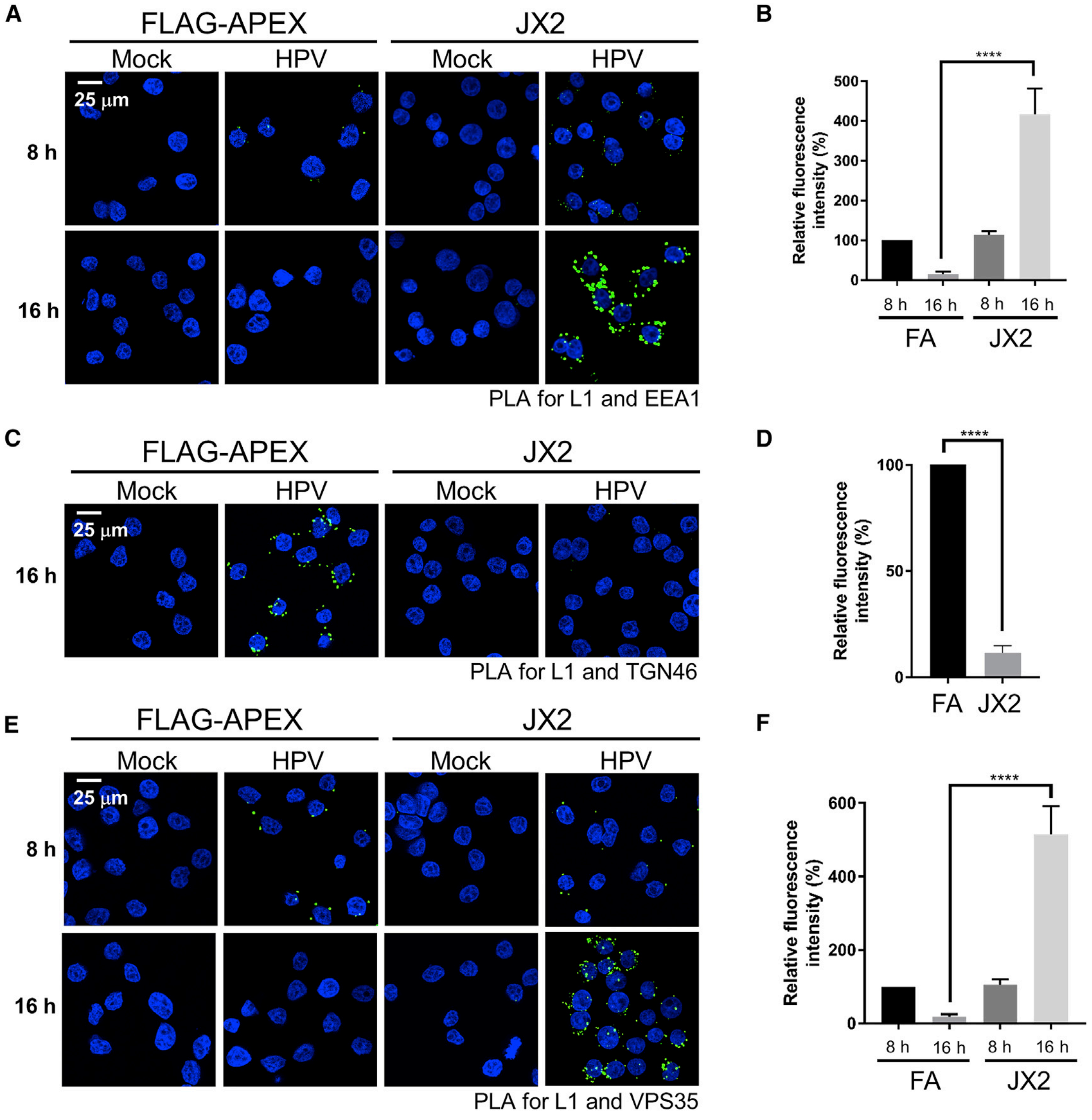

Figure 2. The Traptamer JX2 Causes Accumulation of Incoming HPV in the Endosome and Inhibits Retromer Dissociation

(A) Cells as in Figure 1C were mock-infected or infected with HPV16-HcRed PsV at an MOI of 150. At 8 or 16 h.p.i., a PLA was performed with antibodies recognizing HPV L1 and EEA1 (green). Nuclei were stained with DAPI (blue).

(B) Multiple images as in (A) were processed by BlobFinder software to measure the PLA fluorescence intensity per cell (~200 cells total for each condition). The graphs show mean and standard deviation of three independent experiments normalized to the L1-EEA1 PLA signal of FA cells at 8 h.p.i., set at 100%.

(C) Cells were infected as in (A). At 16 h.p.i., PLA was performed as in (A) with antibodies recognizing L1 and TGN46.

(D) Multiple images obtained as in (C) were processed as in (B). The average fluorescence intensity per cell was normalized to the L1-TGN46 PLA signal of FA cells at 16 h.p.i., set at 100%.

(E) Cells were infected as in (A). At 8 and 16 h.p.i., PLA was performed as in (A) with antibodies recognizing HPV L1 and VPS35.

(F) Multiple images obtained as in (E) were processed as in (B). The average fluorescence intensity per cell was normalized to the L1-VPS35 PLA signal of FA cells at 8 h.p.i., set at 100%.

In all confocal images, a single Z-plane is shown. ****$p < 0.0001$. See also Figures S3 and S4.

5), showing that complex formation between JX2 and TBC1D5 does not require VPS35.

To determine whether HPV infection affected the association of TBC1D5 with JX2, HeLa-tTA cells expressing FA or JX2 were infected with HPV16 PsV at an MOI of 150. Twelve hours later, extracts were prepared and analyzed by co-immunoprecipitation as above. Although the JX2-TBC1D5 interaction was observed in extracts of uninfected cells, as noted above, far more TBC1D5 was co-immunoprecipitated from extracts of infected cells (Figure 3C, compare lane 4 with lane 3), even though infection did not increase the total amount of TBC1D5. Similarly, PLA showed enhanced association between JX2 and TBC1D5 in

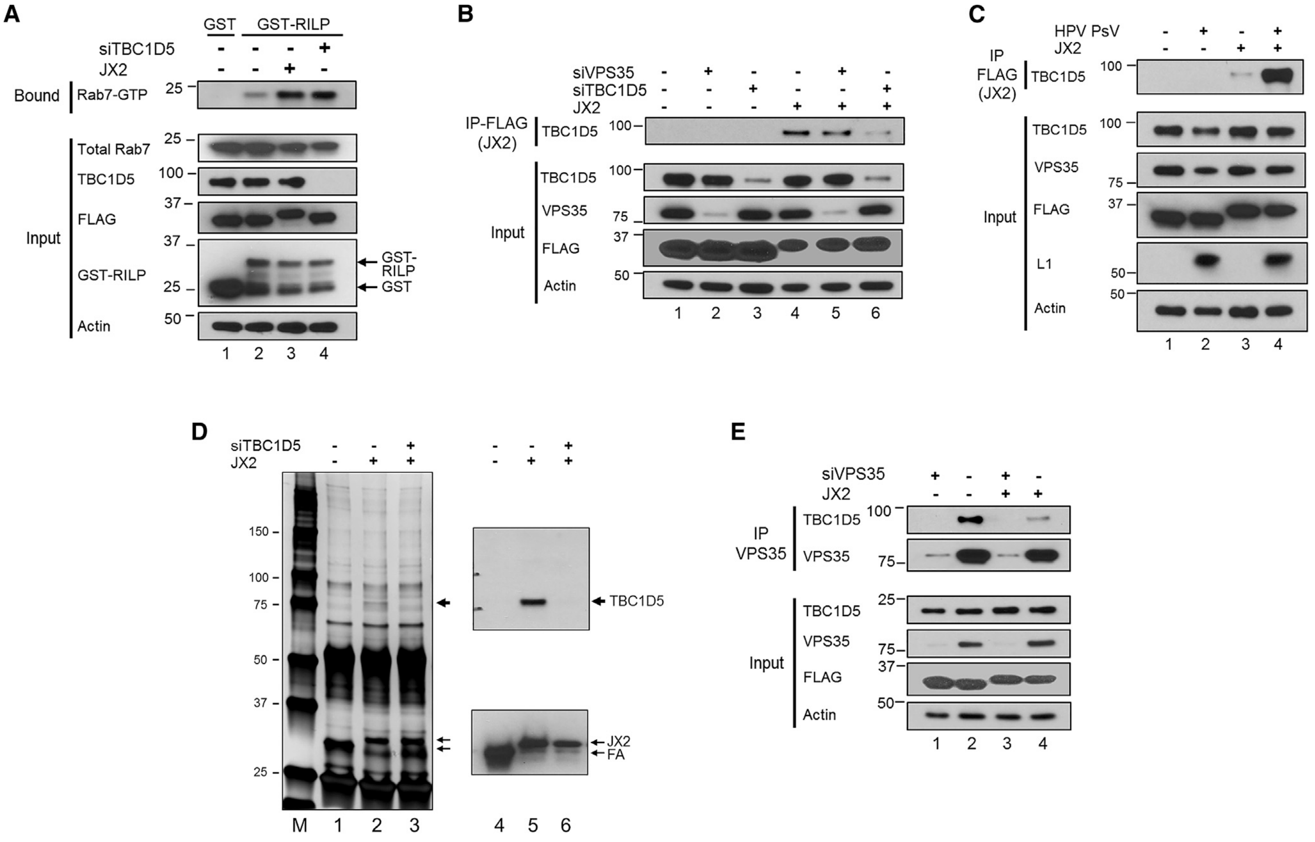

Figure 3. JX2 Forms a Complex with TBC1D5, Inhibits VPS35-TBC1D5 Interaction, and Causes Accumulation of Rab7-GTP

(A) Clonal HeLa-tTA cells stably expressing FA (–) or JX2 (+) in the absence of doxycycline were transfected with non-targeting control scrambled siRNA (–) or TBC1D5 siRNA (+). After 48 h, lysates were pulled down with GST or GST-RILP and subjected to SDS-PAGE. Rab7 was detected by immunoblotting with an antibody that recognizes Rab7A and Rab7B. Samples not pulled down (input) were electrophoresed and immunoblotted for the indicated proteins. Actin is a loading control. The positions of GST and RILP-GST are indicated.

(B) Cells as in (A) were transfected with non-targeting control scrambled siRNA (–) or siRNA targeting VPS35 or TBC1D5 (+). After 48 h, lysates were prepared, and JX2 was immunoprecipitated with anti-FLAG antibody. Samples were subjected to SDS-PAGE and immunoblotted for TBC1D5. Non-immunoprecipitated samples (input) were electrophoresed and blotted for the indicated proteins.

(C) Clonal HeLa-tTA cells expressing FA (–) or JX2 (+) in the absence of doxycycline were mock-infected or infected with HPV16-GFP PsV at an MOI of 150. At 12 h.p.i., samples were collected and processed as in (B).

(D) HeLa-tTA cells expressing FA (–) or JX2 (+) in the absence of doxycycline were transfected with siRNA targeting TBC1D5 or left untreated. Extracts were prepared 2 days later, immunoprecipitated with anti-FLAG antibody, and electrophoresed in parallel on two gels. The gel on the left was stained with silver. Arrowheads show the positions of novel immunoprecipitated bands in cells expressing JX2. M indicates the marker lane. The gel on the right was immunoblotted for TBC1D5 (top) or FLAG (bottom).

(E) Cells as in (A) were transfected with control scrambled siRNA (–) or VPS35 siRNA (+). After 48 h, extracts were prepared and immunoprecipitated with anti-VPS35 antibody and blotted for TBC1D5 and VPS35. Non-immunoprecipitated samples (input) were processed as in (B).

See also Figure S5.

infected cells (Figure S4A), and immunofluorescence showed that infection with wild-type but not DM mutant HPV16 PsV caused dramatic co-localization of JX2 (but not FA) and TBC1D5 (Figure S4B). Thus, as was the case with JX2 and EEA1, co-localization of JX2 and TBC1D5 at the endosome required the TM domain of JX2 and the L2 retromer binding sites.

We also tested whether TBC1D5 might be a TM protein target of JX2. According to some TM prediction programs, TBC1D5 contains two closely spaced hydrophobic segments with lower TM prediction scores than JX2 (Figure S2A). However, in HeLa S3 cells lacking JX2, much TBC1D5 was in the soluble S1 fraction in carbonate extraction experiments, and of the portion of TBC1D5 that was membrane-associated, most was extracted by carbonate (Figure S2C). We conclude that TBC1D5 is not an integral membrane protein. To determine whether the hydrophobic segment of TBC1D5 is required for complex formation with JX2, we knocked down endogenous TBC1D5 and reconstituted expression with full-length TBC1D5, a TBC1D5 mutant lacking the hydrophobic segment (null mutant), or a TBC1D5 mutant in which the hydrophobic segment was replaced with the TM domain of the PDGF β receptor (PRTM mutant). The exogenous wild-type and mutant versions of TBC1D5 were expressed at similar levels, lower than endogenous expression of TBC1D5 (Figure S2D). Co-immunoprecipitation experiments

CellPress
OPEN ACCESS

showed that full-length exogenous TBC1D5, but not the mutants, was present in a complex with JX2 (Figure S2D, top panel; compare lane 4 with lanes 5 and 6). The reconstituted cells were also subjected to carbonate extraction. Full-length exogenous TBC1D5, like the endogenous protein, partitioned between the S2 and P2 fraction as described above (Figure S2E, top panel). In contrast, the null mutant was in the carbonate-extracted S2 fraction, whereas the PRTM mutant was in the carbonate-resistant P2 fraction. These results show that the central hydrophobic segment of TBC1D5 is required for complex formation with JX2 and that it dictates its sensitivity to carbonate extraction. The aberrant fractionation of the TBC1D5 mutants suggests that the mutations are likely to disrupt the proper folding, localization, or topology of TBC1D5, indirectly interfering with its ability to associate with JX2.

To examine the proteins associated with JX2 more globally, we prepared detergent extracts from uninfected HeLa-tTA cells expressing FA or JX2, immunoprecipitated the traptamer with anti-FLAG antibody, and subjected the samples to SDS-PAGE and silver staining. The immunoprecipitates from cells expressing JX2 contained only two detectable proteins missing from the control FA sample, one migrating at ~75 kDa and the other at ~30 kDa (Figure 3D, lane 2). Western blotting identified the 75-kDa band as TBC1D5, consistent with its absence from immunoprecipitates from TBC1D5 knockdown cells, and the 30-kDa band as JX2 (Figure 3D, lanes 4–6). These results suggest that TBC1D5 is one of the major cell proteins specifically associated with JX2.

JX2 Inhibits Complex Formation between Retromer and TBC1D5

We next tested whether JX2 affected the interaction between TBC1D5 and retromer in uninfected HeLa cells. VPS35 immunoprecipitation followed by anti-TBC1D5 immunoblotting confirmed stable complex formation between retromer and TBC1D5 (Figure 3E, lanes 1 and 2). Notably, when extracts were prepared from cells expressing JX2, markedly less TBC1D5 was co-immunoprecipitated by the VPS35 antibody even though the total amounts of VPS35 and TBC1D5 were unchanged (Figure 3E, lane 4). JX2 also inhibited VPS35-TBC1D5 complex formation in HPV-infected cells (data not shown). These results show that JX2 expression interferes with complex formation between retromer and TBC1D5 and suggest that JX2 inhibits the ability of TBC1D5 to function as a Rab7 GAP.

To determine whether the inhibition of retromer-TBC1D5 complex formation by JX2 was specific, we also examined VPS9-domain ankyrin repeat protein (VARP), a cellular protein that binds to the VPS29 subunit of retromer (Fukuda, 2016; Hesketh et al., 2014). Similar levels of VARP were in a stable complex with retromer in the presence and absence of JX2 (Figure S5A). In addition, there was no evident difference in VARP distribution or co-localization with EEA1 or VPS35 in response to JX2 (Figures S5B and S5C).

TBC1D5 Is an HPV Entry Factor Required for Retromer Dissociation and Endosome Exit

If JX2 acts by inhibiting TBC1D5, then TBC1D5 knockdown should phenocopy the effect of JX2 expression. To test this, we used small interfering RNA (siRNA) to knock down TBC1D5 expression in HeLa cells lacking JX2 (Figure 3A, input lane 4). We then infected control and TBC1D5 knockdown cells with HPV16 PsV at an MOI of 2 and assessed infection by flow cytometry. As shown in Figure 4A, TBC1D5 knockdown caused a marked inhibition of infection comparable with the effect of expressing JX2. Furthermore, infectivity in the knockdown cells was partially restored by exogenous expression of full-length TBC1D5, consistent with the lower expression of exogenous TBC1D5 (Figure S2F). These experiments establish that reduced HPV16 PsV infection in the knockdown cells is, in fact, due to TBC1D5 knockdown. The TBC1D5 mutants did not restore infectivity, further suggesting that the mutations affect TBC1D5 structure or function. In contrast, VARP knockdown did not inhibit HPV16 PsV infectivity (Figures S5D–S5F).

PLA experiments showed that TBC1D5 knockdown caused accumulation of HPV16 PsV in the endosome at 16 h.p.i., prevented arrival of HPV in the TGN, and inhibited disassembly of VPS35 from HPV at 16 h.p.i. (Figures 4B–4G). Thus, JX2 expression and knockdown of TBC1D5 have the same effect on HPV infection and trafficking. This finding, together with complex formation between JX2 and TBC1D5, inhibition of retromer-TBC1D5 binding by JX2 expression, and accumulation of Rab7-GTP in response to JX2 expression or TBC1D5 knockdown strongly suggest that JX2 inhibits HPV infection by inhibiting TBC1D5 action. Most importantly, these experiments identify TBC1D5 as an HPV entry factor that acts by regulating retromer function via its effects on Rab7.

HPV Infection Enhances TBC1D5-Retromer Complex Formation and Localization to the Endosome

We next used immunofluorescence and PLA to assess the effect of HPV infection on recruitment of retromer and TBC1D5 to the endosome. HeLa S3 cells were mock-infected or infected at an MOI of 150 with wild-type HPV16 or DM mutant PsV. The DM mutant accumulates in the endosome because it does not bind retromer (Popa et al., 2015). At 12 h.p.i., cells were stained with antibodies recognizing TBC1D5 and EEA1. As shown in Figure 5A, top row, in uninfected cells. TBC1D5 displayed a faint diffuse distribution throughout the cell (with nuclear exclusion) with a few punctate structures and little overlap with dispersed, punctate EEA1 staining. HPV infection caused TBC1D5 and EEA1 to appear in larger, more localized puncta showing extensive overlap (Figure 5A, second row). Redistribution of TBC1D5 to the endosome did not occur in cells infected with the DM mutant (Figure 5A, third row) or in cells knocked down for VPS35 expression and infected with wild-type HPV16 PsV (Figure S6A). These experiments, quantified in Figure 5B, show that HPV16 infection and binding of retromer to the L2 protein recruits TBC1D5 to the endosome. HPV infection also caused colocalization of VPS35 and EEA1 at 8 h.p.i., which returned to baseline levels by 16 h.p.i. (Figures 5C and S6B). The HPV DM mutant PsV did not cause EEA1-VPS35 co-localization (Figures S6C–S6E). Taken together, these data indicate that the HPV L2 C terminus protruding through the endosomal membrane transiently binds retromer and recruits retromer and TBC1D5 to the endosome.

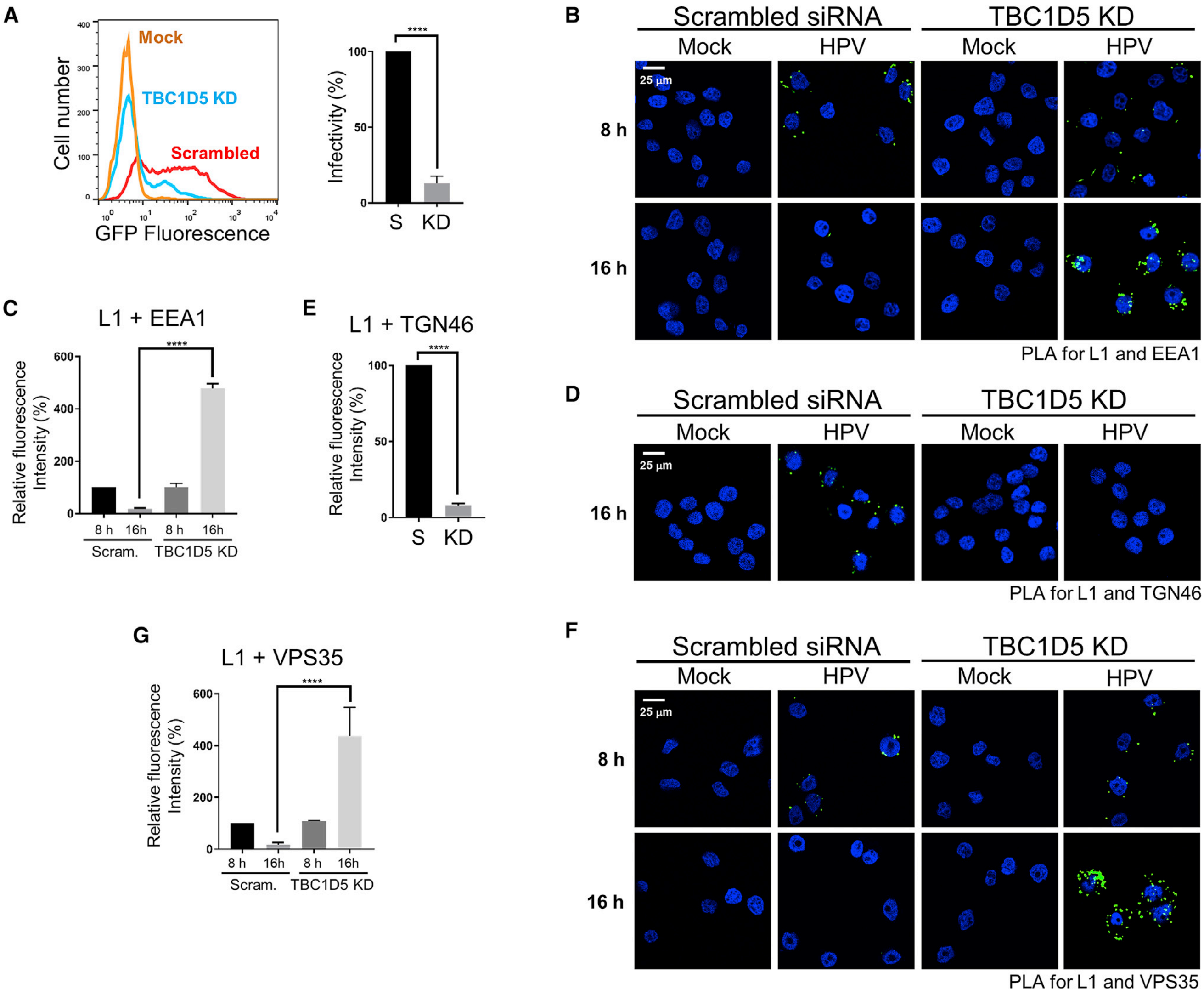

Figure 4. TBC1D5 Is Required for HPV Infection and Disassembly of the Retromer-HPV Complex

(A) HeLa S3 cells were transfected with non-targeting control scrambled siRNA or TBC1D5 siRNA. After 48 h, cells were mock-infected or infected with HPV16-GFP PsV at an MOI of 2. At 48 h.p.i., GFP fluorescence was measured by flow cytometry. Left panel: flow cytometry histograms. Right panel: average results and standard deviation of three independent experiments. Infectivity was normalized to infected cells treated with control siRNA, set at 100%. S, control scrambled siRNA; KD, TBC1D5 knockdown.

(B) HeLa S3 cells transfected with control scrambled siRNA or TBC1D5 siRNA. After 48 h, cells were mock-infected or infected with HPV16-HcRed PsV at an MOI of 150. At 8 and 16 h.p.i., a PLA was performed with antibodies recognizing HPV L1 and EEA1.

(C) Multiple images as in (B) were processed and presented as in Figure 2B.

(D) HeLa S3 cells were transfected and infected as in (B). At 16 h.p.i., a PLA was performed with antibodies recognizing HPV L1 and TGN46.

(E) Multiple images as in (D) were processed and presented as in Figure 2D.

(F) Cells were transfected and infected as in (B). At 8 and 16 h.p.i., a PLA was performed with antibodies recognizing HPV L1 and VPS35.

(G) Multiple images as in (F) were processed and presented as in Figure 2F.

In all confocal images, a single Z-plane is shown. ****$p < 0.0001$. See also Figures S2 and S5.

To test whether HPV infection affects complex formation between TBC1D5 and retromer, we first stained the cells with anti-TBC1D5 and anti-VPS35 antibodies. In uninfected cells, VPS35 staining was observed on punctate structures scattered throughout the cytoplasm with little overlap with TBC1D5 staining (Figures 5D, top row, and S6F). Infection with wild-type HPV PsV but not the HPV DM mutant caused the VPS35-positive puncta to largely co-localize with the TBC1D5-positive puncta (Figures 5D, center and bottom rows, and S6F). These experiments suggest that binding of HPV L2 to retromer facilitates formation of the retromer-TBC1D5 complex at the endosome.

We next performed co-immunoprecipitation experiments to assess assembly of the retromer-TBC1D5 complex in response

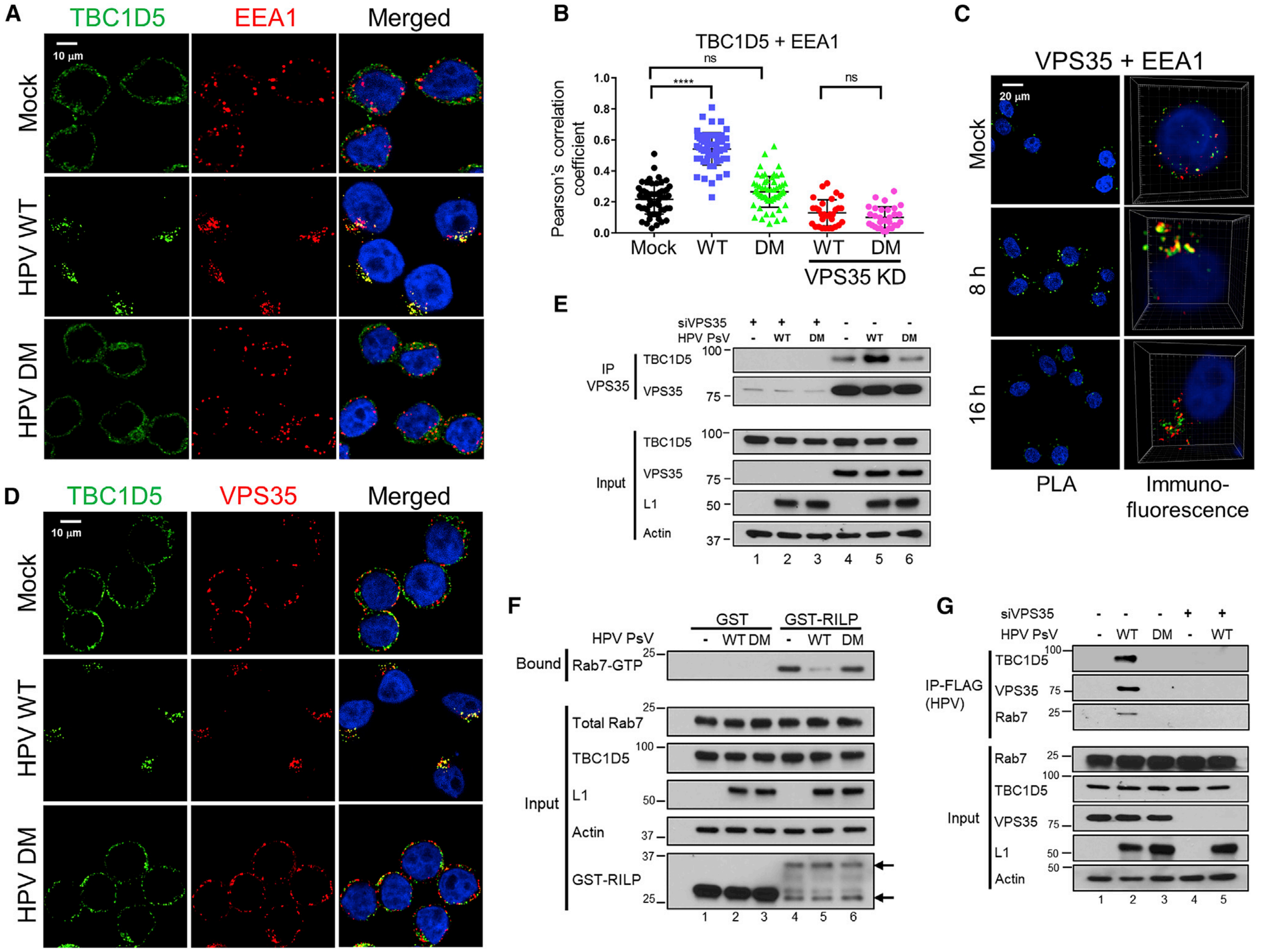

Figure 5. HPV Recruits TBC1D5 to Endosomes via Retromer

(A) HeLa S3 cells transfected with control siRNA or VPS35 siRNA were mock-infected or infected 48 h later with wild-type HPV16-HcRed PsV at an MOI of 150 or with HPV16 DM PsV containing the same number of encapsidated HcRed reporter plasmids. At 12 h.p.i., cells were stained with anti-EEA1 and anti-TBC1D5 antibodies, and fluorescence was visualized by confocal microscopy. TBC1D5, green; EEA1, red; nuclei, blue. Co-localization of EEA1 and TBC1D5 is pseudocolored yellow in the merged panels. These images show cells transfected with control siRNA; VPS35 KD cells are shown in Figure S6A.

(B) Co-localization signal in the merged images as in (A) was quantified by ImageJ software from at least 60 cells in three independent experiments, evaluated by the Pearson's correlation coefficient, and presented as mean and standard deviation. At least 30 cells as in Figure S6A were analyzed similarly. Each dot represents an individual cell. ****p < 0.0001; n.s., not significant.

(C) HeLa S3 cells were mock-infected or infected as in (A) in the absence of transfection. At 8 and 16 h.p.i., cells were subjected to a PLA for VPS35 and EEA1 (left panels) or immunostained with anti-VPS35 (green) and anti-EEA1 (red) (right panels). Overlap between VPS35 and EEA1 staining is pseudocolored yellow in the right panels, which show 3D reconstructions. VPS35-EEA1 PLA signals are quantified in Figure S6B.

(D) HeLa S3 cells were mock-infected or infected as in (A). At 12 h.p.i., cells were stained with anti-TBC1D5 and anti-VPS35 antibodies. TBC1D5, green; VPS35, red; nuclei, blue. Co-localization of VPS35 and TBC1D5 is pseudocolored yellow in the merged panels and quantified in Figure S6F.

(E) HeLa S3 cells were transfected and infected as described in (A). At 12 h.p.i., extracts were prepared and processed as in the legend for Figure 3E to detect VPS35-TBC1D5 complex formation. -, mock-infected; WT, wild-type; DM, double mutant.

(F) HeLa S3 cells were infected as in (A) in the absence of transfection. At 12 h.p.i., extracts were processed with GST or GST-RILP fusion protein as in Figure 3A to detect GTP-Rab7. Lanes identified as in (E). The positions of GST and RILP-GST are indicated by arrows.

(G) HeLa S3 cells were transfected with scrambled siRNA (–) or siRNA targeting VPS35 (+). After 48 h, cells were infected with wild-type HPV16 PsV at an MOI of 150 or DM HPV16 PsV containing the same number of encapsidated reporter plasmids, both containing FLAG-tagged L2. At 12 h.p.i, cells were treated with cross-linker, and HPV PsV were immunoprecipitated with anti-FLAG antibody and immunoblotted with the indicated antibodies. Lanes identified as in (E).

In all confocal images, except as noted in (C), a single Z-plane is shown. See also Figures S3,S4, and S6.

to infection. Infection with wild-type HPV16 PsV increased co-immunoprecipitation between TBC1D5 and VPS35 whereas infection with the DM mutant PsV did not (Figure 5E, lanes 4–6). Importantly, RILP pull-down experiments showed that infection with wild-type but not DM mutant HPV16 PsV decreased the amount of Rab7-GTP without affecting the overall

levels of Rab7 (Figure 5F, lanes 4–6). We also tested whether HPV itself was present in the TBC1D5-retromer complex. HeLa cells were infected with HPV16 PsV containing a FLAG epitope tag at the C terminus of the wild-type or DM mutant L2 protein. At 12 h.p.i., cells were treated with a cell-permeable chemical cross-linker, and detergent extracts were prepared. HPV16 PsV was immunoprecipitated with anti-FLAG antibody, and HPV-associated proteins were detected by SDS-PAGE and immunoblotting. Wild-type HPV16 PsV was in a complex with TBC1D5, VPS35, and Rab7 (Figure 5G, lane 2), and mutation of the retromer binding sites in L2 or VPS35 knockdown eliminated the association of HPV with TBC1D5 and Rab7 (Figure 5G, lanes 3 and 5). Taken together, these results show that HPV binding to retromer enhances complex formation between retromer, Rab7, and TBC1D5 at the endosome, resulting in reduced levels of Rab7-GTP and disassembly of the retromer-HPV complex.

Rab7 Cycling Is Required for Disassembly of the Retromer-HPV Complex and Sorting of HPV into the Retrograde Pathway

The results presented above showed that HPV infection induces complex formation between the Rab7 GAP TBC1D5 and VPS35 at the endosomal membrane, leading to hydrolysis of Rab7-GTP, disassembly of the retromer-HPV complex, and sorting of HPV into the retrograde pathway. To determine the role of Rab7 in HPV trafficking, we directly modulated Rab7 activity. First, the constitutively active Rab7A and Rab7B Q67L mutants, which constitutively bind GTP (Figure 6A, constitutively active [CA] Rab7; (Pan et al., 2006; Harrison et al., 2014; Borg Distefano et al., 2018; Spinosa et al., 2008), were expressed in HeLa cells, and the sensitivity of the cells to HPV16 PsV infection was tested. As shown in Figures 6B, left panel, and S7A, CA Rab7B and, to a lesser extent, Rab7A inhibited HPV infection. Combined expression of both mutants caused more dramatic inhibition. We next tested dominant-negative Rab7A and Rab7B T22N mutants, which bind GDP only (Figure 6A, dominant-negative [DN] Rab7; Borg Distefano et al., 2018; Harrison et al., 2014; Spinosa et al., 2008). DN Rab7B and, to a lesser extent, Rab7A inhibited HPV infection (Figures 6B, center panel, and S7A), similar to results published previously (Day et al., 2013). Combined expression of DN Rab7A and Rab7B caused more profound inhibition. We also used siRNA to deplete Rab7A and Rab7B separately and together and found that Rab7 knockdown also inhibited HPV infection, with Rab7B being more important than Rab7A (Figures 6B, right panel, and S7A).

We performed PLA experiments to assess the effect of combined modulation of Rab7A and Rab7B (collectively referred to as "Rab7" below). PLA for L1 and EEA1 revealed that CA Rab7, DN Rab7, and Rab7 knockdown caused incoming HPV to accumulate in the endosome at 16 h.p.i. and inhibited HPV trafficking to the TGN (Figures 6C, 6D, and S7B–S7D). Thus, manipulations that caused accumulation of Rab7-GTP, accumulation of Rab7-GDP, or global depletion of Rab7 inhibited HPV endosome exit. These results show that cycling of Rab7 between GTP- and GDP-bound forms is essential for sorting of HPV into the retrograde transport pathway. However, a PLA for VPS35 and L1 did reveal an important difference. The CA Rab7 mutants, like TBC1D5 knockdown, did not interfere with retromer-HPV complex assembly at 8 h.p.i. and caused an increased VPS35-L1 PLA signal at 16 h.p.i., indicating that these mutants inhibited retromer disassembly from cargo (Figures 6E and 6F). In contrast, the DN Rab7 mutants prevented assembly of HPV with retromer at both time points (Figures 6E and 6F). This finding confirms the requirement for Rab7-GTP in assembling the complex between retromer and cargo (in this case HPV) at the endosome membrane.

Rab7 Cycling Is Not Required for Trafficking of Cellular Retrograde Cargos

Finally, we tested whether Rab7 cycling is required for trafficking of two cellular retromer cargos, CIMPR and DMT1-II. To analyze CIMPR, we transfected cells with a plasmid expressing the extracellular domain of CD8 fused to the TM and cytoplasmic domain of CIMPR, which contains a retromer binding site that directs sorting to the TGN (Seaman, 2004, 2007). We then incubated live non-permeabilized cells for 3 h at 37°C with an anti-CD8 antibody. Cells were then fixed, permeabilized, and stained with an antibody recognizing TGN46. In control cells, CIMPR trafficked to the TGN, as assessed by strong co-localization of CD8 and TGN46 staining by confocal microscopy (Figures 7A and 7B). CIMPR also trafficked to the TGN in cells knocked down for TBC1D5 or expressing CA Rab7. In contrast, DN Rab7 markedly reduced co-localization of CIMPR and TGN46, indicating impaired sorting of CIMPR into the retrograde pathway. Thus, Rab7-GTP was necessary and sufficient to support trafficking of CIMPR, even though it was not sufficient for HPV trafficking.

To analyze DMT1-II trafficking, we transfected cells with a plasmid expressing a GFP-DMT1-II fusion protein. Twenty-four hours later, we stained fixed cells with anti-GFP and anti-EEA1 antibodies. As shown in Figures 7C and 7D, in TBC1D5 knockdown cells and in cells expressing CA Rab7, DMT1-II did not accumulate in the endosome, suggesting that trafficking was not affected. In contrast, there was markedly increased co-localization of DMT1-II and EEA1 in cells expressing DN Rab7 compared with control cells, indicating that DMT1-II exit from the endosome was impaired in the absence of GTP-Rab7. Taken together, these results show that Rab7 locked in a GTP-bound form could mediate sorting of CIMPR and DMT1-II, but not HPV, into the retrograde trafficking pathway. Thus, Rab7 cycling is not required for trafficking of these cellular cargos, although it is required for HPV (Table S1).

DISCUSSION

Functional genetic screens based on knockdown or knockout of gene expression are powerful tools to identify cellular proteins that act in various biological processes. We developed an alternative screening approach based on the expression of traptamers, artificial proteins that modulate cellular proteins. Traptamer screening can, in principle, be used to identify proteins whose depletion is toxic to cells and proteins that yield the desired phenotype when their activity is enhanced. Here we used the BPV E2 gene expressed from an HPV16 PsV to select HeLa cells resistant to HPV16 infection and recovered traptamer JX2, which caused incoming HPV to accumulate in the

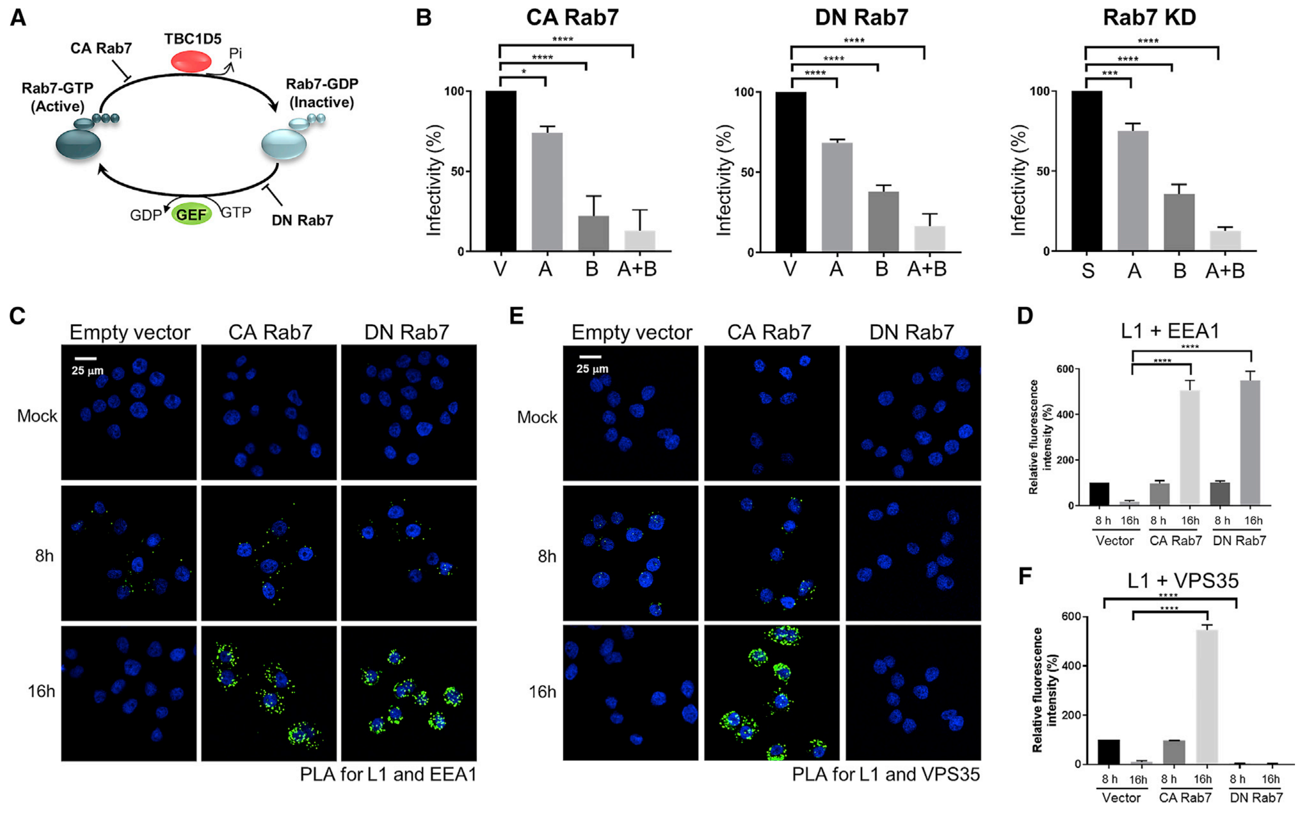

Figure 6. HPV Trafficking Requires Rab7 Cycling

(A) Schematic diagram of the Rab7 cycle and the effects of constitutively active (CA) and dominant-negative (DN) Rab7 mutants. GEF, guanine nucleotide exchange factor.

(B) HeLa-tTA cells stably expressing empty vector or expressing CA or DN Rab7A and/or Rab7B in the absence of doxycycline or knocked down for Rab7A and/or Rab7B were infected with HPV16-GFP PsV at an MOI of 2. Two days later, GFP fluorescence was measured by flow cytometry. The graphs show mean and standard deviation of three independent experiments normalized to control-infected cells, set at 100%. V, vector; A, Rab7A; B, Rab7B; A + B, Rab7A plus Rab7B. Representative primary data are shown in Figure S7A.

(C) HeLa-tTA cells stably expressing empty vector or expressing CA or DN Rab7A plus 7B in the absence of doxycycline were mock-infected or infected with HPV16-HcRed PsV at an MOI of 150. At 8 and 16 h.p.i., a PLA was performed with antibodies recognizing HPV L1 and EEA1. The PLA signal is green, and nuclei were stained with DAPI (blue).

(D) Multiple images as in (C) were processed and presented as in Figure 2B.

(E) Cells as in (C) were infected, and at 8 and 16 h.p.i., a PLA was performed with antibodies recognizing HPV L1 and VPS35.

(F) Multiple images as in (E) were processed and presented as in Figure 2F.

A single Z-plane is shown in all confocal images. ****$p < 0.0001$. See also Figure S7 and Table S1.

endosome and inhibited disassembly of the retromer-HPV16 complex. This phenotype led us to consider the Rab7 axis as a potential target of JX2. We discovered that the Rab7-specific GAP TBC1D5 was in a complex with JX2 and that JX2 inhibited the association between TBC1D5 and retromer, prevented disassembly of the retromer-HPV complex, and caused accumulation of Rab7 in a GTP-bound state. Thus, JX2 is functionally similar to the RidL 131-kDa effector protein of *Legionella pneumophila*, the etiological agent of Legionnaires' disease, which displaces TBC1D5 from the retromer complex (Bärlocher et al., 2017; Romano-Moreno et al., 2017; Yao et al., 2018). The mechanism by which JX2 inhibits TBC1D5 requires the JX2 TM domain, which is also required for coexistence of TBC1D5 and JX2 in a stable complex. We speculate that the TM domain of JX2 binds to and modulates the activity of a still unidentified TM protein in the complex that regulates TBC1D5 activity.

Notably, TBC1D5 knockdown causes the same HPV entry and retromer disassembly defect as JX2 expression. The finding that TBC1D5 knockdown is sufficient to increase Rab7-GTP implies that TBC1D5 is the primary GAP regulating Rab7 activity in these cells, although other GAPs may regulate other aspects of retromer function or HPV entry. Taken together, our results identify TBC1D5 as an HPV entry factor that acts by stimulating hydrolysis of Rab7-bound GTP to GDP. TBC1D5 was not identified in previous knockdown screens for HPV entry factors or proteomics screens for L2-interacting proteins (Lipovsky et al., 2013; Aydin et al., 2014; Bergant Marušič et al., 2012), demonstrating the value of traptamer screening.

Our results provide important insight into the mechanism of TBC1D5 recruitment to the endosomal membrane. Even though TBC1D5 binds retromer tightly *in vitro* (Jia et al., 2016), retromer-TBC1D5 complex formation is increased by HPV infection

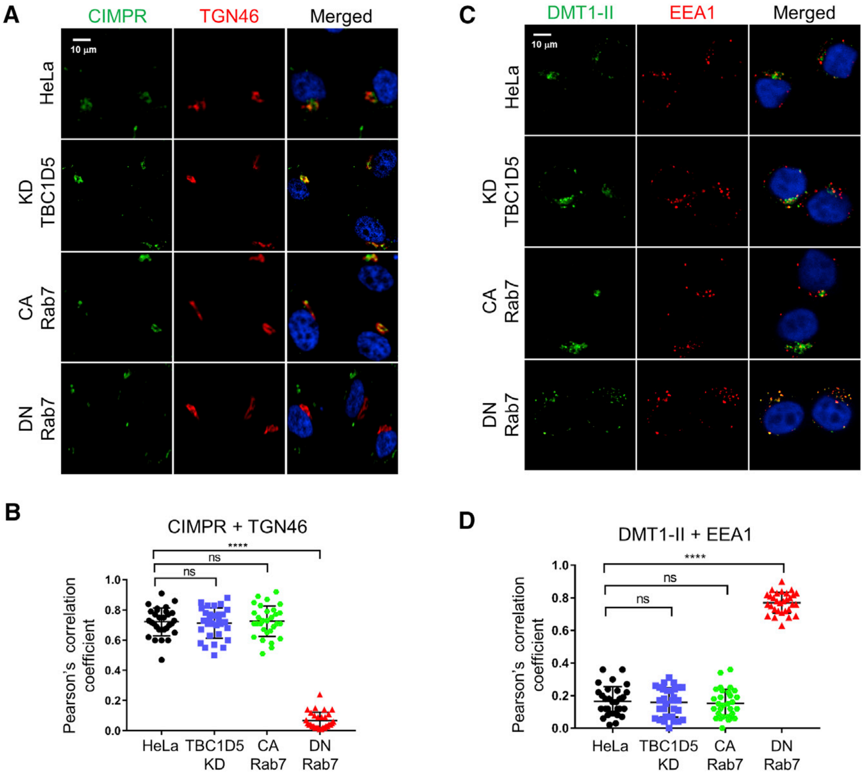

Figure 7. Rab7 Cycling Is Not Required for Trafficking of Cellular Retromer Cargos

(A) HeLa-tTA cells stably expressing empty vector or expressing DN or CA Rab7A plus Rab7B in the absence of doxycycline were transfected with a plasmid expressing the CD8-CIMPR fusion protein. After 24 h, live cells were incubated with anti-CD8 antibody for 3 h at 37°C and then fixed and stained with anti-TGN46. In the second row, cells expressing empty vector were transfected with siRNA targeting TBC1D5 24 h before transfection of the CD8-CIMPR plasmid. CD8-CIMPR, green; TGN46, red; nuclei, blue. Co-localization of CD8-CIMPR and TGN46 is pseudocolored yellow in the merged panels.

(B) Pearson's correlation coefficient for TGN46-CIMPR overlap is shown for 30 cells for each condition, presented as mean and standard deviation. Each dot represents an individual cell.

(C) HeLa-tTA cells as in (A) were transfected with a plasmid expressing GFP-tagged DMT1-II. After 24 h, cells were stained with antibodies recognizing EEA1 and GFP. GFP-DMT1-II, green; EEA1, red; nuclei, blue. Co-localization of GFP-DMT1-II and EEA1 is pseudocolored yellow in the merged panels.

(D) Pearson's correlation coefficient for EEA1-DMT1-II overlap is presented as in (B).

A single Z-plane is shown in all confocal images.

****$p < 0.0001$. See also Table S1.

(Figure 5E), implying that, in intact cells, retromer binding to L2 stabilizes the retromer-TBC1D5 complex. Recruitment of TBC1D5 to retromer at the endosome membrane is dependent on retromer binding to HPV L2, just as SNX3-retromer complex formation and membrane association is enhanced by cargo binding (Harrison et al., 2014; Lucas et al., 2016), highlighting the central role of cargo binding in membrane recruitment and assembly of the active retromer complex. In a negative feedback mechanism, TBC1D5 then acts as a GAP on Rab7-GTP, resulting in GTP hydrolysis, disassembly of the retromer-HPV complex, and dissociation of retromer from the endosome membrane, allowing HPV endosome exit. The transient recruitment of retromer to endosomes (Figure 5C) and the decreased level of Rab7-GTP upon infection with wild-type HPV, but not with an HPV mutant unable to bind retromer (Figure 5F), are consistent with this model. Furthermore, the ability of CA Rab7 to phenocopy the effect of TBC1D5 knockdown on HPV trafficking (see below) implies that the role of TBC1D5 in HPV entry is to stimulate Rab7 GTPase activity.

To test directly whether Rab7 plays a role in HPV trafficking, we used genetic approaches to manipulate Rab7 expression or activity. Exit of HPV from the endosome was blocked by CA Rab7, DN Rab7, or Rab7 knockdown (Figure 6; Table S1), showing that cycling of Rab7 between GTP- and GDP-bound forms, rather than accumulation of GTP- or GDP-bound Rab7, is required for retromer-mediated retrograde sorting of HPV. Increased Rab7-GTP impairs disassembly of retromer from HPV, whereas DN Rab7-GDP blocks HPV-retromer complex formation, but both manipulations inhibit exit of HPV from the endosome, consistent with earlier reports that Rab7-GTP is required to recruit retromer to the endosomal membrane and that disassembly of the retromer-cargo complex is required for trafficking (Harrison et al., 2014; Rojas et al., 2008; Seaman et al., 2009, 2012). In addition, the direct involvement of Rab7 in endosome exit implies that HPV exits the late endosome because Rab7 is thought to primarily act in the later, more acidic stage of the early-to-late endosome continuum.

Strikingly, CA Rab7 or TBC1D5 knockdown did not inhibit trafficking of CIMPR or DMT1-II, showing that Rab7 cycling is not required for trafficking of these cellular cargos (Table S1). Thus, transport of HPV is regulated differently than the cellular cargo. The differential requirement for Rab7 cycling might be due to different affinities of the viral and cellular cargos for retromer or increased avidity of the interaction with HPV due to the multiple copies of L2 in each capsid. Alternatively, different cargos may require a different threshold of GTP-Rab7 for trafficking, or a cellular cargo may be able to utilize multiple transport pathways, including some that do not require Rab7 cycling. Consistent with this final explanation, CIMPR trafficking can proceed by retromer-dependent and -independent pathways (Chen et al., 2019; Cui et al., 2019).

Our findings have implications for the development of agents that target HPV. Agents that regulate Rab7 may be useful in preventing HPV infection in individuals who are not vaccinated against HPV or who do not mount an effective vaccine response. The requirement for Rab7 cycling implies that agents that inhibit generation of either Rab7-GTP or Rab7-GDP will inhibit HPV

entry. Our results also imply that agents that trap Rab7 in its GTP-bound form, including inhibitors of TBC1D5, may block HPV trafficking while sparing cellular cargos.

STAR★METHODS

Detailed methods are provided in the online version of this paper and include the following:

- KEY RESOURCES TABLE
- RESOURCE AVAILABILITY
  - Lead Contact
  - Materials Availability
  - Data and Code Availability
- EXPERIMENTAL MODEL AND SUBJECT DETAILS
  - HeLa S3 cells
  - HaCaT cells
  - HEK293T/17 cells
  - 293TT cells
- METHOD DETAILS
  - Producing HPV pseudovirus
  - Senescence-associated β-galactosidase staining and colony formation
  - Traptamer library construction
  - Selection and recovery of traptamers that block HPV16-BE2 infection
  - Inducible system for traptamer expression
  - SV40 infectivity
  - Transmembrane domain prediction and carbonate extraction assay
  - HPV internalization
  - Proximity ligation assay
  - Split GFP assay
  - RILP pull down assay
  - Co-immunoprecipitation assays of JX2-FLAG and VPS35
  - Generation of VARP knockdown stable cells
  - TBC1D5 rescue experiment
  - Generation and expression of Rab7 mutants
  - Immunofluorescence microscopy
  - Co-immunoprecipitation of FLAG-tagged L2 protein
  - CD8-CIMPR and GFP-DMT1-II trafficking assays
- QUANTIFICATION AND STATISTICAL ANALYSIS
  - Quantitation of PLA assay
  - Quantitation of Split GFP assay
  - Quantitation of immunofluorescence

SUPPLEMENTAL INFORMATION

Supplemental Information can be found online at https://doi.org/10.1016/j.celrep.2020.107750.

ACKNOWLEDGMENTS

We thank C. Burd, P. Zhang, M. Seaman, A. Shelar, M. Tabuchi, and C. Calderwood for reagents and discussions and Jan Zulkeski for editorial assistance. We also thank the Yale Cancer Center Shared Resources. M.C. was supported by National Science Foundation fellowship DGE-1752134 and NIH training grant T32 AI055403. This work was supported by grants from the NIH (P01 CA016038, R01 AI102876, and R01 CA037157 to D.D.).

AUTHOR CONTRIBUTIONS

Conceptualization, J.X., E.N.H., and D.D.; Data Curation, J.X. and M.C.; Funding Acquisition, M.C. and D.D.; Investigation, J.X. and M.C.; Methodology, J.X., E.N.H., and M.C.; Project Administration, D.D.; Supervision, D.D.; Visualization, J.X., M.C., and D.D.; Writing – Original Draft, J.X.

DECLARATION OF INTERESTS

The authors declare no competing interests.

Received: September 30, 2019
Revised: April 16, 2020
Accepted: May 18, 2020
Published: June 9, 2020

REFERENCES

Allalou, A., and Wählby, C. (2009). BlobFinder, a tool for fluorescence microscopy image cytometry. Comput. Methods Programs Biomed. *94*, 58–65.

Aydin, I., Weber, S., Snijder, B., Samperio Ventayol, P., Kühbacher, A., Becker, M., Day, P.M., Schiller, J.T., Kann, M., Pelkmans, L., et al. (2014). Large scale RNAi reveals the requirement of nuclear envelope breakdown for nuclear import of human papillomaviruses. PLoS Pathog. *10*, e1004162.

Aydin, I., Villalonga-Planells, R., Greune, L., Bronnimann, M.P., Calton, C.M., Becker, M., Lai, K.Y., Campos, S.K., Schmidt, M.A., and Schelhaas, M. (2017). A central region in the minor capsid protein of papillomaviruses facilitates viral genome tethering and membrane penetration for mitotic nuclear entry. PLoS Pathog. *13*, e1006308.

Bärlocher, K., Hutter, C.A.J., Swart, A.L., Steiner, B., Welin, A., Hohl, M., Letourneur, F., Seeger, M.A., and Hilbi, H. (2017). Structural insights into Legionella RidL-Vps29 retromer subunit interaction reveal displacement of the regulator TBC1D5. Nat. Commun. *8*, 1543.

Barlowe, C., Orci, L., Yeung, T., Hosobuchi, M., Hamamoto, S., Salama, N., Rexach, M.F., Ravazzola, M., Amherdt, M., and Schekman, R. (1994). COPII: a membrane coat formed by Sec proteins that drive vesicle budding from the endoplasmic reticulum. Cell *77*, 895–907.

Bergant Marušič, M., Ozbun, M.A., Campos, S.K., Myers, M.P., and Banks, L. (2012). Human papillomavirus L2 facilitates viral escape from late endosomes via sorting nexin 17. Traffic *13*, 455–467.

Borg Distefano, M., Hofstad Haugen, L., Wang, Y., Perdreau-Dahl, H., Kjos, I., Jia, D., Morth, J.P., Neefjes, J., Bakke, O., and Progida, C. (2018). TBC1D5 controls the GTPase cycle of Rab7b. J. Cell Sci. *131*, jcs216630.

Broad Institute.. The RNAi Consortium shRNA Library.https://www.broadinstitute.org/rnai-consortium/rnai-consortium-shrna-library.

Buck, C.B., Pastrana, D.V., Lowy, D.R., and Schiller, J.T. (2005). Generation of HPV pseudovirions using transfection and their use in neutralization assays. Methods Mol. Med. *119*, 445–462.

Burd, C., and Cullen, P.J. (2014). Retromer: a master conductor of endosome sorting. Cold Spring Harb. Perspect. Biol. *6*, a016774.

Cammett, T.J., Jun, S.J., Cohen, E.B., Barrera, F.N., Engelman, D.M., and Dimaio, D. (2010). Construction and genetic selection of small transmembrane proteins that activate the human erythropoietin receptor. Proc. Natl. Acad. Sci. USA *107*, 3447–3452.

Chen, K.E., Healy, M.D., and Collins, B.M. (2019). Towards a molecular understanding of endosomal trafficking by Retromer and Retriever. Traffic *20*, 465–478.

Cui, Y., Carosi, J.M., Yang, Z., Ariotti, N., Kerr, M.C., Parton, R.G., Sargeant, T.J., and Teasdale, R.D. (2019). Retromer has a selective function in cargo sorting via endosome transport carriers. J. Cell Biol. *218*, 615–631.

Day, P.M., Thompson, C.D., Schowalter, R.M., Lowy, D.R., and Schiller, J.T. (2013). Identification of a role for the trans-Golgi network in human papillomavirus 16 pseudovirus infection. J. Virol. *87*, 3862–3870.

Day, P.M., Weisberg, A.S., Thompson, C.D., Hughes, M.M., Pang, Y.Y., Lowy, D.R., and Schiller, J.T. (2019). Human papillomavirus 16 capsids mediate nuclear entry during infection. J. Virol. *93*, e00454-19.

Dell'Angelica, E.C., and Bonifacino, J.S. (2019). Coatopathies: Genetic Disorders of Protein Coats. Annu. Rev. Cell Dev. Biol. *35*, 131–168.

DiGiuseppe, S., Luszczek, W., Keiffer, T.R., Bienkowska-Haba, M., Guion, L.G., and Sapp, M.J. (2016). Incoming human papillomavirus type 16 genome resides in a vesicular compartment throughout mitosis. Proc. Natl. Acad. Sci. USA *113*, 6289–6294.

Eugster, A., Frigerio, G., Dale, M., and Duden, R. (2000). COP I domains required for coatomer integrity, and novel interactions with ARF and ARF-GAP. EMBO J. *19*, 3905–3917.

Freeman-Cook, L.L., and DiMaio, D. (2005). Modulation of Cell Function by Small Transmembrane Proteins Modeled on the Bovine Papillomavirus E5 Protein. Oncogene *24*, 7756–7762.

Fujiki, Y., Hubbard, A.L., Fowler, S., and Lazarow, P.B. (1982). Isolation of intracellular membranes by means of sodium carbonate treatment: application to endoplasmic reticulum. J. Cell Biol. *93*, 97–102.

Fukuda, M. (2016). Multiple Roles of VARP in Endosomal Trafficking: Rabs, Retromer Components and R-SNARE VAMP7 Meet on VARP. Traffic *17*, 709–719.

Goodwin, E.C., and DiMaio, D. (2000). Repression of human papillomavirus oncogenes in HeLa cervical carcinoma cells causes the orderly reactivation of dormant tumor suppressor pathways. Proc. Natl. Acad. Sci. USA *97*, 12513–12518.

Goodwin, E.C., Naeger, L.K., Breiding, D.E., Androphy, E.J., and DiMaio, D. (1998). Transactivation-competent bovine papillomavirus E2 protein is specifically required for efficient repression of human papillomavirus oncogene expression and for acute growth inhibition of cervical carcinoma cell lines. J. Virol. *72*, 3925–3934.

Goodwin, E.C., Yang, E., Lee, C.J., Lee, H.W., DiMaio, D., and Hwang, E.S. (2000). Rapid induction of senescence in human cervical carcinoma cells. Proc. Natl. Acad. Sci. USA *97*, 10978–10983.

Guerra, F., and Bucci, C. (2016). Multiple Roles of the Small GTPase Rab7. Cells *5*, 34.

Harbour, M.E., Breusegem, S.Y., Antrobus, R., Freeman, C., Reid, E., and Seaman, M.N. (2010). The cargo-selective retromer complex is a recruiting hub for protein complexes that regulate endosomal tubule dynamics. J. Cell Sci. *123*, 3703–3717.

Harrison, M.S., Hung, C.S., Liu, T.T., Christiano, R., Walther, T.C., and Burd, C.G. (2014). A mechanism for retromer endosomal coat complex assembly with cargo. Proc. Natl. Acad. Sci. USA *111*, 267–272.

Harrison, K., Haga, I.R., Pechenick Jowers, T., Jasim, S., Cintrat, J.C., Gillet, D., Schmitt-John, T., Digard, P., and Beard, P.M. (2016). Vaccinia Virus Uses Retromer-Independent Cellular Retrograde Transport Pathways To Facilitate the Wrapping of Intracellular Mature Virions during Virus Morphogenesis. J. Virol. *90*, 10120–10132.

Heim, E.N., Marston, J.L., Federman, R.S., Edwards, A.P., Karabadzhak, A.G., Petti, L.M., Engelman, D.M., and DiMaio, D. (2015). Biologically active LIL proteins built with minimal chemical diversity. Proc. Natl. Acad. Sci. USA *112*, E4717–E4725.

Hesketh, G.G., Pérez-Dorado, I., Jackson, L.P., Wartosch, L., Schäfer, I.B., Gray, S.R., McCoy, A.J., Zeldin, O.B., Garman, E.F., Harbour, M.E., et al. (2014). VARP is recruited on to endosomes by direct interaction with retromer, where together they function in export to the cell surface. Dev. Cell *29*, 591–606.

Hung, V., Udeshi, N.D., Lam, S.S., Loh, K.H., Cox, K.J., Pedram, K., Carr, S.A., and Ting, A.Y. (2016). Spatially resolved proteomic mapping in living cells with the engineered peroxidase APEX2. Nat. Protoc. *11*, 456–475.

Jia, D., Zhang, J.S., Li, F., Wang, J., Deng, Z., White, M.A., Osborne, D.G., Phillips-Krawczak, C., Gomez, T.S., Li, H., et al. (2016). Structural and mechanistic insights into regulation of the retromer coat by TBC1d5. Nat. Commun. *7*, 13305.

Jimenez-Orgaz, A., Kvainickas, A., Nägele, H., Denner, J., Eimer, S., Dengjel, J., and Steinberg, F. (2018). Control of RAB7 activity and localization through the retromer-TBC1D5 complex enables RAB7-dependent mitophagy. EMBO J. *37*, 235–254.

Lipovsky, A., Popa, A., Pimienta, G., Wyler, M., Bhan, A., Kuruvilla, L., Guie, M.A., Poffenberger, A.C., Nelson, C.D., Atwood, W.J., and DiMaio, D. (2013). Genome-wide siRNA screen identifies the retromer as a cellular entry factor for human papillomavirus. Proc. Natl. Acad. Sci. USA *110*, 7452–7457.

Lipovsky, A., Zhang, W., Iwasaki, A., and Dimaio, D. (2015). Application of the proximity-dependent assay and fluorescence imaging approaches to study viral entry pathways. Methods Mol. Biol. *1270*, 437–451.

Liu, T.T., Gomez, T.S., Sackey, B.K., Billadeau, D.D., and Burd, C.G. (2012). Rab GTPase regulation of retromer-mediated cargo export during endosome maturation. Mol. Biol. Cell *23*, 2505–2515.

Lucas, M., Gershlick, D.C., Vidaurrazaga, A., Rojas, A.L., Bonifacino, J.S., and Hierro, A. (2016). Structural Mechanism for Cargo Recognition by the Retromer Complex. Cell *167*, 1623–1635.e14.

Orcl, L., Palmer, D.J., Amherdt, M., and Rothman, J.E. (1993). Coated vesicle assembly in the Golgi requires only coatomer and ARF proteins from the cytosol. Nature *364*, 732–734.

Palmer, D.J., Helms, J.B., Beckers, C.J., Orci, L., and Rothman, J.E. (1993). Binding of coatomer to Golgi membranes requires ADP-ribosylation factor. J. Biol. Chem. *268*, 12083–12089.

Pan, X., Eathiraj, S., Munson, M., and Lambright, D.G. (2006). TBC-domain GAPs for Rab GTPases accelerate GTP hydrolysis by a dual-finger mechanism. Nature *442*, 303–306.

Popa, A., Zhang, W., Harrison, M.S., Goodner, K., Kazakov, T., Goodwin, E.C., Lipovsky, A., Burd, C.G., and DiMaio, D. (2015). Direct binding of retromer to human papillomavirus type 16 minor capsid protein L2 mediates endosome exit during viral infection. PLoS Pathog. *11*, e1004699.

Priya, A., Kalaidzidis, I.V., Kalaidzidis, Y., Lambright, D., and Datta, S. (2015). Molecular insights into Rab7-mediated endosomal recruitment of core retromer: deciphering the role of Vps26 and Vps35. Traffic *16*, 68–84.

Rojas, R., van Vlijmen, T., Mardones, G.A., Prabhu, Y., Rojas, A.L., Mohammed, S., Heck, A.J., Raposo, G., van der Sluijs, P., and Bonifacino, J.S. (2008). Regulation of retromer recruitment to endosomes by sequential action of Rab5 and Rab7. J. Cell Biol. *183*, 513–526.

Romano-Moreno, M., Rojas, A.L., Williamson, C.D., Gershlick, D.C., Lucas, M., Isupov, M.N., Bonifacino, J.S., Machner, M.P., and Hierro, A. (2017). Molecular mechanism for the subversion of the retromer coat by the *Legionella* effector RidL. Proc. Natl. Acad. Sci. USA *114*, E11151–E11160.

Schelhaas, M., Shah, B., Holzer, M., Blattmann, P., Kühling, L., Day, P.M., Schiller, J.T., and Helenius, A. (2012). Entry of human papillomavirus type 16 by actin-dependent, clathrin- and lipid raft-independent endocytosis. PLoS Pathog. *8*, e1002657.

Seaman, M.N. (2004). Cargo-selective endosomal sorting for retrieval to the Golgi requires retromer. J. Cell Biol. *165*, 111–122.

Seaman, M.N.J. (2007). Identification of a novel conserved sorting motif required for retromer-mediated endosome-to-TGN retrieval. J. Cell Sci. *120*, 2378–2389.

Seaman, M.N. (2012). The retromer complex - endosomal protein recycling and beyond. J. Cell Sci. *125*, 4693–4702.

Seaman, M.N., Harbour, M.E., Tattersall, D., Read, E., and Bright, N. (2009). Membrane recruitment of the cargo-selective retromer subcomplex is catalysed by the small GTPase Rab7 and inhibited by the Rab-GAP TBC1D5. J. Cell Sci. *122*, 2371–2382.

Seaman, M.N.J., Mukadam, A.S., and Breusegem, S.Y. (2018). Inhibition of TBC1D5 activates Rab7a and can enhance the function of the retromer cargo-selective complex. J. Cell Sci. *131*, jcs217398.

Siddiqa, A., Broniarczyk, J., and Banks, L. (2018a). Papillomaviruses and Endocytic Trafficking. Int. J. Mol. Sci. *19*, E2619.

Siddiqa, A., Massimi, P., Pim, D., Broniarczyk, J., and Banks, L. (2018b). Human Papillomavirus 16 Infection Induces VAP-Dependent Endosomal Tubulation. J. Virol. *92*, e01514–e01517.

Spinosa, M.R., Progida, C., De Luca, A., Colucci, A.M., Alifano, P., and Bucci, C. (2008). Functional characterization of Rab7 mutant proteins associated with Charcot-Marie-Tooth type 2B disease. J. Neurosci. *28*, 1640–1648.

Stroupe, C. (2018). This Is the End: Regulation of Rab7 Nucleotide Binding in Endolysosomal Trafficking and Autophagy. Front. Cell Dev. Biol. *6*, 129.

Sun, J., Deghmane, A.E., Bucci, C., and Hmama, Z. (2009). Detection of activated Rab7 GTPase with an immobilized RILP probe. Methods Mol. Biol. *531*, 57–69.

Szafer, E., Rotman, M., and Cassel, D. (2001). Regulation of GTP hydrolysis on ADP-ribosylation factor-1 at the Golgi membrane. J. Biol. Chem. *276*, 47834–47839.

Tabuchi, M., Yanatori, I., Kawai, Y., and Kishi, F. (2010). Retromer-mediated direct sorting is required for proper endosomal recycling of the mammalian iron transporter DMT1. J. Cell Sci. *123*, 756–766.

Vardarajan, B.N., Bruesegem, S.Y., Harbour, M.E., Inzelberg, R., Friedland, R., St George-Hyslop, P., Seaman, M.N., and Farrer, L.A. (2012). Identification of Alzheimer disease-associated variants in genes that regulate retromer function. Neurobiol. Aging *33*, 2231.e15–2231.e30.

Yao, J., Yang, F., Sun, X., Wang, S., Gan, N., Liu, Q., Liu, D., Zhang, X., Niu, D., Wei, Y., et al. (2018). Mechanism of inhibition of retromer transport by the bacterial effector RidL. Proc. Natl. Acad. Sci. USA *115*, E1446–E1454.

Yoshihisa, T., Barlowe, C., and Schekman, R. (1993). Requirement for a GTPase-activating protein in vesicle budding from the endoplasmic reticulum. Science *259*, 1466–1468.

Young, J.M., Zine El Abidine, A., Gómez-Martinez, R.A., and Ozbun, M.A. (2019). The known and potential intersections of Rab-GTPases in human papillomavirus infections. Front. Cell Dev. Biol. *7*, 139.

Zhang, W., Kazakov, T., Popa, A., and DiMaio, D. (2014). Vesicular trafficking of incoming human papillomavirus 16 to the Golgi apparatus and endoplasmic reticulum requires γ-secretase activity. MBio *5*, e01777–e14.

Zhang, P., Monteiro da Silva, G., Deatherage, C., Burd, C., and DiMaio, D. (2018). Cell-penetrating peptide mediates intracellular membrane passage of human papillomavirus L2 protein to trigger retrograde trafficking. Cell *174*, 1465–1476.e13.

Cell Reports
Article

STAR★METHODS

KEY RESOURCES TABLE

REAGENT or RESOURCE	SOURCE	IDENTIFIER
Antibodies		
Mouse anti-Human Papillomavirus type 16 L1 protein clone CAMVIR-1 (RUO)	BD Biosciences	Cat#554171; RRID:N/A
Mouse monoclonal antibody anti-FLAG M2	Sigma Aldrich	Cat#F3165; RRID: AB_259529
Rabbit polyclonal antibody anti-TGN46	Abcam	Cat#ab50595 RRID:AB_2203289
Rabbit monoclonal antibody anti-VPS35 [EPR11501(B)]	Abcam	Cat#ab157220 RRID:AB_2636885
Mouse monoclonal antibody anti-VPS35	Abcam	Cat#ab57632 RRID:AB_946126
Goat polyclonal antibody anti-VPS35	Abcam	Cat#ab10099 RRID: AB_296841
Mouse monoclonal antibody anti-EEA1	BD Biosciences	Cat#610457 RRID: AB_397830
Rabbit monoclonal antibody anti-EEA1	Cell Signaling Technology	Cat#2411 RRID: AB_2096814
Rabbit polyclonal antibody anti-TBC1D5	Abcam	Cat#ab203896 RRID:N/A
Mouse monoclonal antibody anti-TBC1D5 (E-9)	Santa Cruz	Cat#sc-376296 RRID: AB_10988434
Rabbit polyclonal antibody anti-VARP	Abcam	Cat#ab108216 RRID: AB_10860158
Rabbit polyclonal antibody anti Pan-actin	Cell Signaling Technology	Cat#4968 RRID: AB_2313904
Rabbit monoclonal antibody anti-Rab7 (D95F2)	Cell Signaling Technology	Cat#9367 RRID: AB_1904103
Mouse monoclonal antibody anti-CD8 alpha (C8/468)	Abcam	Cat#ab187279 RRID: N/A
Mouse monoclonal antibody anti-GFP (B-2)	Santa Cruz	Cat#SC-9996 RRID: AB_627695
Rabbit monoclonal antibody anti-PDI (C81H6)	Cell Signaling Technology	Cat#3501 RRID: AB_2156433
Rabbit polyclonal antibody anti-VPS26	Abcam	Cat#ab23892 RRID: AB_2215043
Rat monoclonal antibody anti-BAP31 (CC-1)	Thermo Fisher	Cat#MA3-002 RRID: AB_781566
Rabbit polyclonal antibody anti-EGFR	Abcam	Cat#ab2430 RRID: AB_303065
Anti-FLAG M2 affinity gel	Sigma	Cat#A2220
Mouse monoclonal ANTI-FLAG® M2-Peroxidase (HRP) antibody produced in mouse	Sigma	Cat#A8592 RRID: N/A
Mouse monoclonal GST Antibody (B-14) HRP	Santa Cruz	Cat# sc-138 HRP RRID: N/A
Donkey anti-Mouse IgG (H+L) Highly Cross-Adsorbed Secondary Antibody, Alexa Fluor 488	Thermo Fisher	Cat#A-21202 RRID: AB_141607
Donkey anti-Rabbit IgG (H+L) Highly Cross-Adsorbed Secondary Antibody, Alexa Fluor 488	Thermo Fisher	Cat#A-21206 RRID: AB_2535792
Goat anti-Mouse IgG (H+L) Highly Cross-Adsorbed Secondary Antibody, Alexa Fluor 568	Thermo Fisher	Cat#A-11004 RRID: AB_2534072
Donkey anti-Rabbit IgG (H+L) Highly Cross-Adsorbed Secondary Antibody, Alexa Fluor 568	Thermo Fisher	Cat#A-10042 RRID: AB_2534017
Bacterial and Virus Strains		
One Shot Stbl3 Chemically Competent *E. coli*	Thermo Fisher	Cat#C737303
Rosetta 2(DE3) Competent Cells-Novagen	Millipore	Cat#71397
MegaX DH10B T1R Electrocomp Cells	Invitrogen	Cat#640003
Chemicals, and Recombinant Proteins		
Paraformaldehyde	Electron Microscopy Sciences	Cat#15710
Saponin	Sigma Aldrich	Cat#47036
Hoechst 33324, Trihydrochloride, Trihydrate	Thermo Fisher	Cat#H3570
Puromycin dihydrochloride	Sigma Aldrich	Cat#P-7255
Hygromycin B	Invitrogen	Cat#10687010
OptiPrep	Axis-Shield	Cat#AXS-1114542

(*Continued on next page*)

CellPress
OPEN ACCESS

Continued

REAGENT or RESOURCE	SOURCE	IDENTIFIER
Halt Protease and Phosphatase Inhibitor Single-Use Cocktail, EDTA-Free	Thermo Fisher	Cat#78443
RNase A, DNase and protease-free	Thermo Fisher	Cat#EN0531
Phusion High-Fidelity DAN Polymerase	New England Biolabs	Cat#M0530L
T4 DNA Ligase	New England Biolabs	Cat#M0202L
PCR Nucleotide Mix	Sigma Aldrich	Cat# 11581295001
**D**ulbecco's Modified Eagle's Medium – high glucose	Sigma Aldrich	Cat#D5671
Opti-MEM	GIBCO	Cat#31985-070
Fetal Bovine Serum	Atlanta Biologicals	Cat#S11150
L-Glutamine	Thermo Fisher	Cat#25030081
Pen Strep (Penicillin Streptomycin)	Thermo Fisher	Cat#15140122
Trypsin-EDTA (0.25%)	Thermo Fisher	Cat#25200056
Dulbecco's Phosphate Buffered Saline (DPBS), with $MgCl_2$ and $CaCl_2$	Sigma Aldrich	Cat#D8662
B-PER Bacterial Protein Extraction Reagent	Thermo Fisher	Cat#78243
DNase I solution	Sigma Aldrich	Cat#90082
Lysozyme solution	Sigma Aldrich	Cat#90083
Glutathione Sepharose	GE Healthcare	Cat#17075601
Protein A/G PLUS-Agarose	Santa Cruz	Cat#sc-2003
Critical Commercial Assays		
Duolink® *In Situ* PLA Probe Anti-Mouse PLUS	Sigma-Aldrich	Cat#DUO92001
Duolink® *In Situ* PLA Probe Anti-Rabbit MINUS	Sigma-Aldrich	Cat#DUO92005
Duolink® *In Situ* Detection Reagents Green	Sigma-Aldrich	Cat#DUO92014
In-Fusion HD cloning Kits	Clontech	Cat#638909
RNase-Free DNase Set	QIAGEN	Cat#79254
DNeasy Blood and Tissue Kits	QIAGEN	Cat#69504
iQ™SYBR Green Supermix	Bio-Rad	Cat#1708880
Lipofectamine® RNAiMAX Transfection Reagent	Thermo Fisher	Cat#13778100
TransIT-HeLaMONSTER® Transfection Kit	Mirus Bio	Cat# MIR2904
BCA Protein Assay Kit	Thermo Fisher	Cat#23225
Senescence β-Galactosidase Staining Kit	Cell Signaling Technology	Cat#9860
Experimental Models: Cell Lines		
HaCaT	AddexBio Technologies	Cat#T0020001
HeLa S3	ATCC	Cat#CCL-2.2 RRID:CVCL_0058
HaCaT/GFP1-10NES	Zhang et al., 2018	N/A
293TT	Buck et al., 2005	RRID:CVCL_1D85
293T	ATCC	Cat#CRL-3216 RRID:CVCL_0063
Oligonucleotides		
ON-TARGETplus human TBC1D5 siRNA (smartpool)	Dharmacon	Cat#L-020775-01-0005
ON-TARGETplus human VPS35 siRNA (smartpool)	Dharmacon	Cat#L-010894-00-0005
ON-TARGETplus humanRAB7A siRNA (smartpool)	Dharmacon	Cat# L-010388-00-0005
ON-TARGETplus human RAB7B siRNA (smartpool)	Dharmacon	Cat#L-018225-00-0005
Individual: ON-TARGETplus human TBC1D5 siRNA Targeted Region:ORF	Dharmacon	Cat# J-020775-09-0002
Individual: ON-TARGETplus human TBC1D5 siRNA Targeted Region:5′UTR,ORF	Dharmacon	Cat# J-020775-10-0002

(*Continued on next page*)

Continued

REAGENT or RESOURCE	SOURCE	IDENTIFIER
Individual: ON-TARGETplus human TBC1D5 siRNA Targeted Region:3′UTR	Dharmacon	Cat# J-020775-11-0002
Individual: ON-TARGETplus human TBC1D5 siRNA Targeted Region:ORF	Dharmacon	Cat# J-020775-12-0002
ON-TARGETplus Non-targeting siRNA Control Pool	Dharmacon	Cat# D-001810-10-05
HcRed Forward primer: GCACCCAGAGCATG AGAAT	Zhang et al., 2018	N/A
HcRed Reverse primer: TCGTAGGTGGTGGTT CTCT	Zhang et al., 2018	N/A
shRNA for VARP hairpin 1	Broad Institute	TRCN0000148842
shRNA for VARP hairpin 2	Broad Institute	TRCN0000148894
shRNA for VARP hairpin 3	Broad Institute	TRCN0000148965
Recombinant DNA		
$pMSCV_{puro}$	Clontech	Cat#634401
pCAG-HcRed	Addgene	Cat#11152
HPV p16sheLL	Buck et al., 2005	Addgene; Cat#37320
HPV p5sheLL	Buck et al., 2005	Addgene; Cat#46953
HPV p18sheLL	Buck et al., 2005	Addgene; Cat#37321
p16sheLL-CPP-GFP11	Zhang et al., 2018	N/A
pLenti CMV GFP Puro (658-5)	Addgene	Cat#17448
pLenti CMV GFP1-10NES	Zhang et al., 2018	N/A
pCD8-CIMPR	Popa et al., 2015; Seaman, 2007	N/A
psPAX2	Addgene	Cat#12260
pMD2.G	Addgene	Cat#12259
pRetroX-Tet-off Advanced	Clontech	Cat#632105
pRetroX-Tight-Pur	Clontech	Cat#632104
pRetroX-Tight-Hyg	Clontech	Cat#631034
pIRES neo-GFP-DMT1-II	Tabuchi et al., 2010	N/A
pGEX KG-GST-RILP	Christopher Burd	N/A
pCMV-SPORT6-TBC1D5	Dharmacon	Cat# MHS6278-202758175
pT_{hygro}-TBC1D5, full length	This paper	N/A
pT_{hygro}-TBC1D5, null mutant	This paper	N/A
pT_{hygro}-TBC1D5, TM replacement mutant	This paper	N/A
pT_{puro}-FA	This paper	N/A
pT_{puro}-JX2	This paper	N/A
pT_{puro}-JX2NA	This paper	N/A
pET28 Rab7A	Christopher Burd	N/A
pT_{puro}-Rab7A WT	This paper	N/A
pT_{puro}-Rab7A Q67L	This paper	N/A
pT_{puro}-Rab7A T22N	This paper	N/A
pT_{hygro}-Rab7A WT	This paper	N/A
pT_{hygro}-Rab7A Q67L	This paper	N/A
pT_{hygro}-Rab7A T22N	This paper	N/A
pCMV-SPORT6-Rab7B	Dharmacon	Cat#MHS6278-202756137
pT_{puro}-Rab7B WT	This paper	N/A
pT_{puro}-Rab7B Q67L	This paper	N/A
pT_{puro}-Rab7B T22N	This paper	N/A
pT_{hygro}-Rab7B WT	This paper	N/A

(*Continued on next page*)

Continued

REAGENT or RESOURCE	SOURCE	IDENTIFIER
pT_{hygro}-Rab7B Q67L	This paper	N/A
pT_{hygro}-Rab7B T22N	This paper	N/A
pCAG-BE2-IRES-GFP	This paper	N/A
Software and Algorithms		
FlowJo	FLOWJO, LLC	https://www.flowjo.com/solutions/flowjo RRID:SCR_008520
Fiji	National Institutes of Health, USA	http://fiji.sc RRID:SCR_002285
GraphPad Prism	GraphPad Software	https://www.graphpad.com/ RRID:SCR_002798
BlobFinder	The Centre for Image Analysis at Uppsala University	http://www.cb.uu.se/~amin/BlobFinder/ RRID:SCR_015788
IMARIS	Oxford Instruments	https://imaris.oxinst.com/ RRID: N/A
Leica LAS X	Leica	https://www.leica-microsystems.com/products/microscope-software/p/leica-las-x-ls/downloads/ RRID: N/A
Other		
SW55 Ti rotor	Beckman Coulter	Cat#342196
Thinwall Polypropylene Tubes	Beckman Coulter	Cat#326819
Slide-A-Lyzer Dialysis Cassettes, gamma-irradiated, 10,000 MWCO	Thermo Fisher	Cat#66453
Optima XPN-80 Ultracentrifuge	Beckman Coulter	Cat#A95765
Stratedigm S1000Exi Flow Cytometer	Stratedigm	N/A
SP5 confocal microscope	Leica	N/A
GloMax® Explorer Multimode Microplate Reader	Promega	N/A
Tube, Thinwall, Ultra-Clear, 800 μL	Beckman Coulter	Cat#344090
Kimble® 885300-0002 Kontes® 2mL All Glass Dounce Tissue Grinder with Large & Small Pestles	Capitol Scientific	KIM-885300-0002
Amicon Ultra-0.5 Centrifugal Filter Unit with molecular weight cut-off of 3 kDa	Millipore	UFC500324

RESOURCE AVAILABILITY

Lead Contact

Further information and requests for resources and reagents should be directed to and will be fulfilled by the Lead Contact, Daniel DiMaio (daniel.dimaio@yale.edu).

Materials Availability

All unique reagents generated in this study will be made available on request, but we may require a payment and/or a completed Materials Transfer Agreement if there is potential for commercial application.

Data and Code Availability

This study did not generate datasets or code.

EXPERIMENTAL MODEL AND SUBJECT DETAILS

HeLa S3 cells

HeLa S3 cells, purchased from American Type Culture Collection (ATCC), are a clonal derivative of the parent human female HeLa cell line. Cells were cultured at 37°C in DMEM (Sigma Aldrich) with HEPES and L-glutamine, supplemented with 10% fetal bovine serum (FBS) and 100 units/mL penicillin streptomycin, in 5% CO_2. For infectivity experiments, 1×10^5 HeLa S3 cells were seeded in 12-well plates and cultured in the same medium for 16 h before infection. For cellular uptake experiments, 5×10^4 HeLa S3 cells were seeded in eight-chambered glass coverslips and cultured in the same medium overnight. Cell line was authenticated by confirming expression of HPV18 E6/E7 oncogene expression. All cell lines were periodically tested for mycoplasma contamination.

HaCaT cells

HaCaT cells purchased from AddexBio Technologies are spontaneously transformed keratinocytes from human male histologically normal skin. HaCaT cells were cultured at 37°C in DMEM with HEPES and L-glutamine, supplemented with 10% FBS and 100 units/mL penicillin streptomycin, in 5% CO_2. To generate HaCaT/GFP1-10NES stable cell line, 3×10^4 HaCaT cells were seeded in 24-well plates and cultured in the same medium for 16 h before transduction. HaCaT/GFP1-10NES cells stably expressing GFP1-10NES were cultured at 37°C in DMEM with HEPES and L-glutamine, supplemented with 10% FBS, 100 units/mL penicillin streptomycin, and 1 μg/mL puromycin (Sigma Aldrich), in 5% CO_2.

HEK293T/17 cells

HEK293T/17 (293T) cells used for making lentiviruses were purchased from ATCC. 293T is a female human embryonic kidney (HEK) cell line, carries a stably expressed Simian virus 40 (SV40) genome, and expresses large T antigen. Clone 17 was selected specifically for its high transfectability. 293T cells were cultured at 37°C in DMEM with HEPES and L-glutamine, supplemented with 10% FBS and 100 units/mL penicillin streptomycin, in 5% CO_2. For producing lentiviruses, 2×10^6 cells were seeded in 100 mm dishes and cultured in the same medium overnight. Cell line was authenticated by confirming expression of nuclear SV40 large T antigen.

293TT cells

293TT cells, obtained from Christopher Buck (NIH), were generated by introducing SV40 Large T antigen cDNA into female HEK293T cells to increase Large T antigen expression. 293TT were cultured at 37°C in DMEM with HEPES and L-glutamine, supplemented with 10% FBS, 100 units/mL penicillin streptomycin, and 250 μg/mL hygromycin, in 5% CO_2. To produce PsVs, 8×10^6 cells were seeded in 150 mm dishes and cultured in the same medium for 16 h before transfection. Cell line was authenticated by confirming expression of nuclear SV40 large T antigen.

METHOD DETAILS

Producing HPV pseudovirus

We constructed the pCAG-BE2-IRES-GFP plasmid by inserting a gBlock (Integrated DNA Technologies, Iowa, USA) encoding BPV E2 and an internal ribosome entry site (IRES) into pCAG-GFP (Addgene #11150) digested with EcoRI and AgeI. HPV PsVs were produced by using polyethylenimine (PEI) to co-transfect 293TT cells with pCINeo-GFP (obtained from Christopher Buck (NIH)), pCAG-HcRed (Addgene #11152) or pCAG-BE2-IRES-GFP, together with wild-type p16sheLL, p5sheLL, p18sheLL (Buck et al., 2005) or double mutant (DM) p16sheLL-DM, which contains mutations that inactivate the retromer binding sites in L2 (Popa et al., 2015). For split GFP experiments, we produced PsV by using p16sheLL-CPP-GFP11, which contains seven GFP11 repeats at the C terminus of L2, as described (Zhang et al., 2018). Packaged PsVs were purified by density gradient centrifugation in OptiPrep as described (Buck et al., 2005), and the integrity of the stocks was assessed by monitoring L1 and L2 levels following SDS-PAGE. Wild-type PsVs were titered by flow cytometry for fluorescence two days after infection of HeLa S3 cells. For experiments comparing wild-type and DM HPV16 PsVs, encapsidated reporter plasmids were quantified by qPCR as described (Zhang et al., 2018), and PsV stocks containing the same number of wild-type and mutant encapsidated reporter plasmids were used to infect cells. Plasmids for PsV stocks used in the same experiment were quantified in parallel.

Senescence-associated β-galactosidase staining and colony formation

1×10^5 HeLa S3 cells were seeded on 6 cm^2 plates. After 16 h, cells were infected by HPV16-BE2 PsV at MOI of 5. A second infection was done at the same MOI one day later, and the growth medium was changed every two days. After 14 days, cells were stained for senescence-associated β-galactosidase activity according to the manufacturer's instructions (Cell Signaling Technology).

To determine the appropriate MOI of HPV16-BE2 PsV to use in the traptamer screen, 1×10^5 HeLa S3 cells were seeded on 6 cm^2 plates. After 16 h, cells were infected by HPV16-BE2 PsV at MOI of 1, 5, 10 and 20. A second infection was done at the same MOI one day later, and the growth medium was changed every 2 days. After 14 days, cells were fixed in methanol and stained with 5% Giemsa solution (Sigma-Aldrich).

Traptamer library construction

$pMSCV_{puro}$-FA was constructed by digesting $pMSCV_{puro}$ with BglII and EcoRI and inserting a BglII to EcoRI gBlock fragment encoding FLAG-APEX2 (Hung et al., 2016) and containing a BamHI site immediately upstream of the stop codon. To construct the EHFA library expressing traptamers with a randomized, predominantly hydrophobic segment, a long 5′ degenerate oligonucleotide (FWD long) that encodes the hydrophobic segment was synthesized. FWD long consisted of a 5′ BamHI restriction site followed by a library specific 5′ primer binding site for amplification, 26 randomized codons and 2 tandem stop codons. In the randomized segment, the composition of A:G:C:T was 1:1:1:1 at the first position of each codon; 1:1:1:7 at the second position; and 0:1:1:1 at the third position (Table S2). This oligonucleotide encodes a protein sequence comprised of ~80% hydrophobic amino acids. To convert FWD long to double-stranded DNA, it was annealed to a non-degenerate oligonucleotide (REV long) that was complementary to the tandem stop codons and 3′ sequence in FWD long (Table S2). Extension was performed by PCR consisting of 10 ng of each long oligonucleotide along with Pfu reaction buffer, 0.2 mM of each dNTP, and 1 μL Pfu Turbo polymerase (Agilent Technologies, Inc.) in a total volume of

100 μL. PCR settings were 94°C for 5 min; two cycles of 94°C for 1 min, 38°C for 1 min, and 72°C for 1 min; and 72°C for 10 min. To amplify the double-stranded DNA, short primers corresponding to the 5′ ends of the FWD and REV long oligonucleotides (FWD short and REV short in Table S2) were added to the reaction at a final concentration of 500 nM, and PCR was performed by the following settings: 94°C for 5 min; 30 cycles of 94°C for 1 min, 41°C for 1 min, and 72°C for 1 min; 72°C for 10 min; hold at 4°C. The amplified products were digested with BamHI and EcoRI, purified, and ligated into the $pMSCV_{puro}$-FA retroviral vector digested with the same restriction endonucleases. After transformation of *Escherichia coli* strain DH10β with the ligation reaction, approximately ~250,000 ampicillin-resistant colonies were pooled. Plasmid DNA was extracted from this pool to generate the EHFA library. To confirm the amino acid composition and structure of clones, plasmid DNA from randomly picked individual ampicillin-resistant colonies was sequenced.

Selection and recovery of traptamers that block HPV16-BE2 infection

The EHFA library was transfected into 293T cells together with pCL-Eco packaging plasmid and pVSV-G, which expresses the vesicular stomatitis virus G protein, to generate a retrovirus stock. Transfection with Lipofectamine 2000 (Invitrogen) was used in all transfections to produce retroviruses. One million HeLa S3 cells in each of five 10 cm^2 plates were infected with EHFA retrovirus stock at MOI of ~0.2 with 4 μg/mL polybrene. Forty-eight h.p.i., 0.5 μg/mL puromycin was added and the selection was imposed for four days. For the screen, one million puromycin-resistant cells per plate were seeded in ten 15 cm^2 plates. After 16 h, the cells were infected with HPV16-BE2 PsV at MOI of 20. A second HPV16-BE2 infection was done at the same MOI one day later, and the growth medium was changed every 2 days. Fourteen days after the first infection, individual colonies of proliferating cells were isolated using cloning cylinders. Cells were expanded, and genomic DNA was isolated using DNeasy Blood and Tissue Kit (QIAGEN).

Retroviral inserts in genomic DNA from the expanded cells were recovered by using PCR with short primers specific to the library (FWD short and REV short in Table S2) (PCR procedure as in Heim et al., 2015). PCR products were purified, digested with BamHI and EcoRI, and cloned into $pMSCV_{puro}$-FA vector.

Inducible system for traptamer expression

$pRetroX\text{-}Tight_{puro}$ (pT_{puro}) vector without a BamHI site (pT-WOB) was constructed by PCR-directed mutagenesis of pT_{puro} vector (Clontech #632104). The FA fragment was amplified from $pMSCV_{puro}$-FA and cloned into pT-WOB vector at the NotI and EcoRI sites, generating pT-FA. To construct pT-JX2, the FA-traptamer BamHI plus EcoRI fragment from $pMSCV_{puro}$-FA-JX2 was cloned into pT-FA. pT-JX2NA, which does not contain the APEX2 fragment, was constructed from pT-WOB and a gBlock encoding FLAG-JX2NA. Retrovirus expressing Tetracycline-controlled transactivator protein tTA was produced from pRetroX-Tet-off Advanced plasmid (Tet-off) (Clontech). HeLa S3 and HaCaT cells were transduced with the tTA retrovirus and selected with neomycin to construct HeLa-tTA cells. HeLa-tTA cells were then transduced with retrovirus produced from pT-JX2, pT-JX2NA, or pT-FA, and cells were selected with puromycin. Unless specified otherwise, all experiments were performed in the absence of doxycycline to obtain maximal traptamer expression.

SV40 infectivity

SV40 was produced in CV-1 cells as previously described (Goodwin et al., 1998). To measure SV40 infection, 1×10^5 HeLa S3 cells expressing JX2 or control cells in 12-well plates were incubated with SV40 at MOI of 0.5. Forty-eight h.p.i., cells were harvested and fixed in cold methanol. Infectivity was measured by staining for intracellular SV40 large T antigen with a 1:50 dilution of the Sc-147 antibody (Santa Cruz, Inc.), and flow cytometry.

Transmembrane domain prediction and carbonate extraction assay

The following TM domain prediction programs were used: Phobius (http://phobius.sbc.su.se/); TMHMM (http://www.cbs.dtu.dk/services/TMHMM/); and DAS (https://tmdas.bioinfo.se/DAS/index.html). The sequence of human TBC1D5 was downloaded from uniport (https://www.uniprot.org/uniprot/Q92609).

2×10^7 HeLa S3 cells expressing endogenous TBC1D5 or mutant TBC1D5 and transfected with TBC1D5 siRNA or clonal HeLa cells expressing pT-JX2 or pT-FA in the absence of doxycycline were plated in one 15 cm^2 plate for each condition. Sixteen hours later, cells were harvested and resuspended in 500 μL of swelling buffer (10 mM HEPES [pH 7.5], 1.5 mM $MgCl_2$, 10 mM KCl, and 0.5 mM DTT) supplemented with 1X Halt protease and phosphatase inhibitor cocktail (Thermo Fisher). The cell suspension was incubated on ice for 30 min and then mechanically homogenized using a 2 mL Dounce homogenizer with the B pestle. The cell homogenate was centrifuged at 16.1k x g for 10 min at 4°C to remove intact cells and nuclei. The resulting supernatant (fraction T) was centrifuged at 100k x g in a SW55Ti rotor for 30 min at 4°C. The supernatant was saved as fraction S1. The pellet containing membranes was then washed with HN buffer (50 mM HEPES [pH 7.5], 150 mM NaCl supplemented with 1X Halt protease and phosphatase inhibitor cocktail) and centrifuged at 100k x g in a SW55Ti rotor for 10 min at 4°C. The pellet (P1) was resuspended in 50 μL of buffer containing 10 mM Tris [pH 7.5], 150 mM NaCl, 5 mM DTT, and 2 mM $MgCl_2$ for 15 min on ice and then mixed with 500 μL of solution containing 0.1 M Na_2CO_3 (pH ~11.7) for 30 min on ice. The carbonate-extracted preparation was then centrifuged at 100k x g in a SW55Ti rotor for 30 min. The supernatant (fraction S2) was concentrated with Amicon ultracentrifugal filters (3 kDa molecular weight cut-off). The pellet after centrifugation was washed in cold HN buffer and recentrifuged as above to generate pellet fraction P2. P2 was dissolved in 50 μL Laemmli loading buffer. All fractions were analyzed by SDS-PAGE and immunoblotting.

HPV internalization

3.5 × 10^4 clonal HeLa-tTA cells expressing pT-JX2 or pT-FA in the absence of doxycycline were infected on glass coverslips in 24-well plates with HPV16 PsV containing a GFP reporter plasmid at MOI of 50. After 8 h, cells were fixed with 4% paraformaldehyde (Electron Microscopy Sciences) and permeabilized with 1% Saponin (Sigma-Aldrich) at room temperature for 1 h. Cells were immunostained with anti-HPV16 L1 antibody (BD #554171), followed by 1:200 dilution of AlexaFluor-conjugated secondary antibody (Thermo Fisher). The slides were mounted in mounting solution with DAPI (Abcam, ab104139), and images were recorded on a Leica SP5 confocal microscope.

Proximity ligation assay

3.5 × 10^4 clonal HeLa-tTA cells expressing pT-JX2 or pT-FA in the absence of doxycycline were infected on glass coverslips in 24-well plates with HPV PsV containing HcRed reporter plasmid at MOI of 150. For knockdown experiments, 2.5 × 10^4 HeLa cells seeded on coverslips in 24-well plates were transfected with 10 nM siRNA for 48 h and then infected with HPV PsV at MOI of 150. Lipofectamine RNAiMAX (Invitrogen) was used for all siRNA transfections. Infected cells were fixed and permeabilized as above at 8 or 16 h.p.i. as indicated. Cells were then incubated with pairs of antibodies (one from mouse and one from rabbit) (source and dilutions of antibodies listed in Table S3). PLA was carried out with Duolink reagents from Sigma Aldrich according to the manufacturer's directions as described (Lipovsky et al., 2015, Zhang et al., 2018). Briefly, cells were incubated with a pair of suitable PLA antibody probes in a humidified chamber, which were then subjected to ligation and amplification with fluorescent substrate at 37°C. The slides were mounted in mounting solution with DAPI. Cells were imaged with a Leica SP5 confocal microscope. Images were processed by Fiji and quantified by Blobfinder software to measure fluorescence intensity in each sample. The average fluorescence intensity per cell was normalized to the appropriate control sample as indicated in each experiment.

Split GFP assay

To generate cells expressing cytoplasmic GFP1-10NES, lentiviruses were produced by co-transfecting 293T cells with $pLenti_{puro}$ GFP1-10NES (Zhang et al., 2018), lentiviral packaging plasmid psPAX2, and envelope plasmid pMD2.G. Two days later, the lentiviral supernatant was harvested and filtered. HaCaT cells were infected with GFP1-10NES lentivirus and selected for 2 days in medium containing 1 μg/mL puromycin. Single cells were then plated in 96-well plates, and monoclonal cell strains were isolated. To generate stable cell lines expressing both GFP1-10NES and JX2 (HaCaT GFP1-10NES+JX2), the FA-JX2 DNA fragment was amplified from pT_{puro}-JX2 and ligated into $pLenti_{hygro}$ vector by using BamHI and SalI to generate $pLenti_{hygro}$-JX2. $Lenti_{hygro}$-JX2 lentivirus produced in 293T cells was used to infect stable GFP1-10NES HaCaT cells, and cells were selected by hygromycin treatment. To demonstrate cytoplasmic protrusion of L2 during PsV infection, 3 × 10^4 pooled HaCaT GFP1-10NES-JX2 cells seeded on eight-chambered glass slides were incubated for 3h at MOI of 2000 with HPV16 containing an HcRed reporter plasmid and wild-type L2 or L2 containing GFP11 inserted at the C terminus (HPV16-CPP-GFP11; [Zhang et al., 2018]). Nuclei were stained with Hoescht 33342 and live cells were analyzed with a Leica SP5 confocal microscope.

RILP pull down assay

The GST-RILP plasmid (obtained from Christopher Burd, Yale University) in pGEX KG vector (Addgene #77103) or pGEX KG expressing GST alone was transformed into *E. coli* strain BL21. Bacterial cell cultures with an OD of ~0.4 were induced by 0.8 mM isopropyl β-D-1-thiogalactopyranoside (IPTG) at 21°C for 18 h. Bacteria were harvested and lysed with B-per lysis buffer (Thermo Scientific) and purified by using a pre-equilibrated slurry of glutathione beads (Thermo Scientific, #16100) in 50 mM Tris, 150 mM NaCl, 1mM $MgCl_2$, pH 8.0. Purified proteins were eluted from the beads with reduced 20mM glutathione and exchanged into HEPES buffer (20 mM HEPES [pH 7.4], 50 mM NaCl, 5 mM $MgCl_2$, 1 mM DTT) by dialysis and quantified by BCA protein assay. 2 × 10^5 clonal HeLa-tTA cells expressing pT-JX2 or pT-FA in the absence of doxycycline were plated in 6 cm^2 plates. After 12 h, cells were transfected with 10 nM TBC1D5 or control scrambled siRNA and infected 48 h later with HPV16 PsV at MOI of 150. At 12 h.p.i., cells were lysed with 400 μL ice-cold lysis buffer (20 mM HEPES [pH 7.4], 50 mM NaCl, 5 mM $MgCl_2$, 1 mM DTT and 0.15% Triton X-100) supplemented with 1 X Halt protease and phosphatase inhibitor cocktail (Thermo Fisher). The lysate was centrifuged at 14,000 rpm for 20 min at 4°C. The supernatant was incubated with 15 μg of purified GST or GST-RILP at 4°C for 2 h. Then 40 μL of pre-equilibrated slurry of glutathione beads were added and the complex was incubated at 4°C for another 3 h, followed by two washes in lysis buffer. Bound proteins were eluted with 40 μL of 2 X SDS sample buffer at 100°C, followed by SDS-PAGE and immunoblotting after normalization for total extracted protein, as determined by BCA protein assay. A portion of the clarified total lysate was electrophoresed as Input. HRP-conjugated secondary antibodies (donkey) were used at 1:3000. Signal was detected with Supersignal West Pico or Femto Chemiluminescent Substrate (Thermo Fisher).

Co-immunoprecipitation assays of JX2-FLAG and VPS35

3.5 × 10^5 clonal HeLa-tTA cells expressing pT-JX2 or pT-FA in the absence of doxycycline were seeded in 6 cm^2 plates and infected with HPV16 PsV at MOI of 150. For knockdown experiments, 2 × 10^5 HeLa S3 cells expressing JX2 or empty vector were plated in 6 cm^2 plates, and after 16 h cells were transfected with 10 nM siRNA. Forty-eight h later, cells were infected with HPV16 PsV at MOI of 150. At 12 h.p.i., cells were washed with cold PBS and lysed with 400 μL ice-cold lysis buffer (20 mM HEPES [pH 7.4], 50 mM NaCl, 5 mM $MgCl_2$, 1 mM DTT and 0.2% Triton X-100) supplemented with 1X Halt protease and phosphatase inhibitor cocktail (Thermo

Fisher). The lysate was clarified by centrifugation at 14,000 rpm for 20 min at 4°C. Immunoprecipitation was carried out by adding anti-FLAG affinity gel (Sigma #A2220) or anti-VPS35 (Abcam, ab10099) antibody and protein A/G-PLUS-agarose beads to the clarified cell lysates at 4°C for 4 h, followed by 5 washes with lysis buffer. Protein complexes were eluted from the beads by heating to 100°C in 2X SDS sample buffer. Eluates and the Input were normalized for total extracted protein, as determined by bicinchoninic acid (BCA) protein assay and analyzed by SDS-PAGE followed by immunoblotting or staining with Silver Stain Kit (Pierce).

Generation of VARP knockdown stable cells

To generate stable VARP knockdown cell lines, pLKO.1 vectors encoding a VARP shRNA were transfected into 293T cells together with pSPAX2 and pMD2.G packaging plasmids to produce lentiviruses expressing VARP shRNA (Broad Institute.,). HeLa S3 cells were transduced with an individual lentivirus and selected with puromycin.

TBC1D5 rescue experiment

The full-length wild-type *TBC1D5* gene fragment was amplified from pCMV-SPORT6-TBC1D5 (Dharmacon) and cloned into pT_{hygro} vector by using NotI and MluI restriction sites. Plasmids expressing TBC1D5 without the hydrophobic central region (from K354 to K407, null mutant) and TBC1D5 containing the PDGF β receptor TM domain in place of the central hydrophobic region (PRTM mutant) were generated by PCR-directed mutagenesis of wild-type pT_{hygro}-TBC1D5. Retrovirus for each construct was produced in 293T cells as described above. HeLa-tTA cells expressing pT-FA in the absence of doxycycline were transduced with the retroviruses expressing wild-type or mutant TBC1D5 and selected with hygromycin. siRNA targeting the *TBC1D5* 3′-UTR (Dharmacon, J-020775-11) was transfected to knockdown endogenous TBC1D5.

Generation and expression of Rab7 mutants

Q67L and T22N mutant Rab7A fragments were amplified from pET28 Rab7A constructs (obtained from Christopher Burd) and cloned into the pT_{puro} vector at the BamHI and EcoRI sites. The wild-type Rab7B fragment was amplified from Rab7B cDNA in pCMV-SPORT6 vector (Dharmacon) and cloned into the pT_{hygro} vector (Clontech #631034) at the BamHI and EcoRI sites. The Q67L and T22N mutant Rab7B fragments were generated by PCR-directed mutagenesis of pT_{hygro}-Rab7B construct. Retrovirus expressing each construct was produced in 293T cells as described above. HeLa S3 cells were transduced with Rab7A retroviruses and selected with puromycin or with Rab7B retroviruses and selected with hygromycin. To generate stable cell lines co-expressing Rab7A and Rab7B mutants, HeLa-tTA cells were sequentially transduced with Rab7A retrovirus and selected with puromycin and then transduced with Rab7B retrovirus and selected with hygromycin.

Immunofluorescence microscopy

2.5×10^4 HeLa S3 cells were seeded on glass coverslips in 24-well plates and transfected 16 h later with 10 nM VPS35 or control siRNA. Forty-eight hours after transfection, cells were mock-infected or infected with wild-type HPV16 PsV at MOI of 150 or with DM mutant PsV containing the same number of encapsidated HcRed reporter plasmids. At 12 h.p.i., cells were fixed for 15 min at room temperature with 4% paraformaldehyde, permeabilized with 1% saponin for 30 min, and incubated with 1:400 anti-FLAG mouse antibody (Sigma, F3165), 1:100 anti-EEA1 mouse (BD, 610457) or rabbit (CST, 2411) antibody, 1:75 anti-TBC1D5 rabbit (Abcam, ab203896) or mouse (Santa Cruz, SC-376296) antibody, 1:100 anti-VPS35 goat antibody (Abcam, ab10099), or 1:120 anti-VARP rabbit antibody (Abcam, ab108216). Cells were then incubated at room temperature for 1 h with 1:200 AlexaFluor-conjugated secondary antibodies (Life Technologies). The slides were mounted as above, and images were captured using a Leica SP5 confocal microscope. In most experiments, a single Z-plane is shown. In Figure 5C, right panels, sequential Z-planes spaced by ~0.2 micron were recorded for three-dimensional reconstruction with the Imaris software package.

Co-immunoprecipitation of FLAG-tagged L2 protein

2×10^5 HeLa S3 cells were plated in 6 cm^2 plates. After 12 h, cells were transfected with 10 nM siRNA for 48 h and then mock-infected or infected at MOI of 150 with HPV16 PsV containing FLAG-tagged L2. At 12 h.p.i., cells were washed twice with PBS, once with 25 mM sodium phosphate (pH 7.4), and cross-linked with 1.5 mM DSP [dithiobis(succinimidyl propionate)] in 25 mM sodium phosphate buffer (pH 7.4) for 30 min at room temperature. The reaction was quenched with 100 mM Tris HCl (pH 7.4) for 15 min at room temperature. The cells were then washed with cold PBS and lysed with 400 μL of lysis buffer (20 mM HEPES [pH 7.4], 50 mM NaCl, 5 mM $MgCl_2$, 1 mM DTT and 0.15% Triton X-100) supplemented with 1 X Halt protease and phosphatase inhibitor cocktail (Thermo Fisher). The lysate was centrifuged at 14,000 rpm for 20 min at 4°C. Protein concentration was determined by BCA assay. As input, 40 μL of supernatant was reserved. The remainder of the supernatant was incubated with 40 μL of pre-blocked anti-FLAG affinity gel and gently rocked for 3 h at 4°C. Bound proteins were eluted with 40 μL of 2X SDS sample buffer heated to 100°C, followed by SDS-PAGE and immunoblotting with appropriate antibodies. HRP-conjugated secondary antibodies (donkey) were used at 1:3000 and signal was detected with Supersignal West Pico or Femto Chemiluminescent Substrate.

CD8-CIMPR and GFP-DMT1-II trafficking assays

2.5×10^4 HeLa-tTA cells expressing pT_{puro} and pT_{hygro}; pT-CA-Rab7A plus pT-CA-Rab7B; or pT-DN-Rab7A plus pT-DN-Rab7B; or transfected with 10 nM TBC1D5 or control siRNA in the absence of doxycycline were seeded on coverslips in 24-well plates. After an

additional 24 h, 1 μg of CD8-CIMPR plasmid (obtained from Matthew Seaman, Cambridge Institute for Medical Research) was transfected into the cells by using Trans-IT HeLaMONSTER reagent (Mirus Bio). Twenty-four hours later, unfixed cells were incubated for 3 h at 37°C with a 1:20 dilution of mouse antibody recognizing the extracellular domain of CD8 (Abcam, ab187279). The cells were then fixed for 15 min at room temperature with 4% paraformaldehyde, permeabilized with 1% Saponin for 30 min, and stained with 1:200 anti-TGN46 (Abcam, ab50595), followed by 1:200 AlexaFluor-conjugated secondary antibodies (Life Technologies). The slides were mounted in mounting solution with DAPI and images were acquired with a Leica SP5 confocal microscope.

2.5×10^4 HeLa-tTA cells expressing pT_{puro} and pT_{hygro}; pT-CA-Rab7A plus pT-CA-Rab7B; or pT-DN-Rab7A plus pT-DN-Rab7B in the absence of doxycycline transfected with 10 nM TBC1D5 or control siRNA were seeded on the coverslips in 24-well plates. Twenty-four hours later, 1 μg of GFP-DMT1-II plasmid (obtained from Mitsuaki Tabuchi, Kagawa University) (Tabuchi et al., 2010) was transfected into the cells. After an additional 24 h, cells were fixed and permeabilized as above and incubated with 1:100 anti-GFP mouse antibody (Santa Cruz, B-2) and 1:75 anti-EEA1 rabbit antibody (CST, 2411), followed by staining with 1:200 AlexaFluor-conjugated secondary antibodies (Life Technologies). Slides were mounted as above, and images were acquired with a Leica SP5 confocal microscope.

QUANTIFICATION AND STATISTICAL ANALYSIS

Quantitation of PLA assay

Images obtained from PLA experiments were processed by BlobFinder software which performs a single cell analysis and quantifies the fluorescence signal intensity per cell for each sample (Allalou and Wählby, 2009). The signal intensity of at least 200 cells for each condition was averaged in each experiment. The average intensity for L1 plus EEA1, L1 plus VPS35, FLAG (JX2) plus TBC1D5, and EEA1 plus VPS35 samples was normalized to cells infected with wide-type HPV16 PsV at 8 h.p.i.; the average intensity for L1 plus TGN46 samples was normalized to cells infected with wild-type HPV16 PsV at 16 h.p.i. The relative fluorescence intensity of three independent experiments were averaged and plotted. Statistical analysis was performed using GraphPad Prism 7 software. Results are presented as mean and standard deviation. Comparisons between control and experimental groups were made using one-way ANOVA or unpaired two-tailed Student's t tests. p values of $p < 0.05$ were considered to indicate statistical significance.

Quantitation of Split GFP assay

Reconstituted GFP signal was quantified using Fiji software. The corrected total cellular fluorescence (CTCF) = Integrated density – (Area of selected cell X Mean fluorescence of background readings), was calculated. The CTCF of at least 100 cells for each condition was quantified and averaged. Statistical analysis was performed using GraphPad Prism 7 software. Data are presented as mean and standard deviation. Student's t tests were performed to assess statistical significance between control and experimental groups. p values of $p < 0.05$ were considered to indicate statistical significance.

Quantitation of immunofluorescence

For co-localization tests, Pearson's correlation coefficient between the respective channels was obtained in ImageJ by using coloc2 plugin. At least 30 cells were analyzed for each condition. Statistical analysis was performed using GraphPad Prism 7 software. Results are presented as mean and standard deviation, with each cell represented by a single symbol. Comparisons between control and experimental groups were made using one-way ANOVA tests. p values of $p < 0.05$ were considered to indicate statistical significance.

Cell Reports, Volume *31*

Supplemental Information

TBC1D5-Catalyzed Cycling of Rab7 Is Required for Retromer-Mediated Human Papillomavirus Trafficking during Virus Entry

Jian Xie, Erin N. Heim, Mac Crite, and Daniel DiMaio

Figure S1

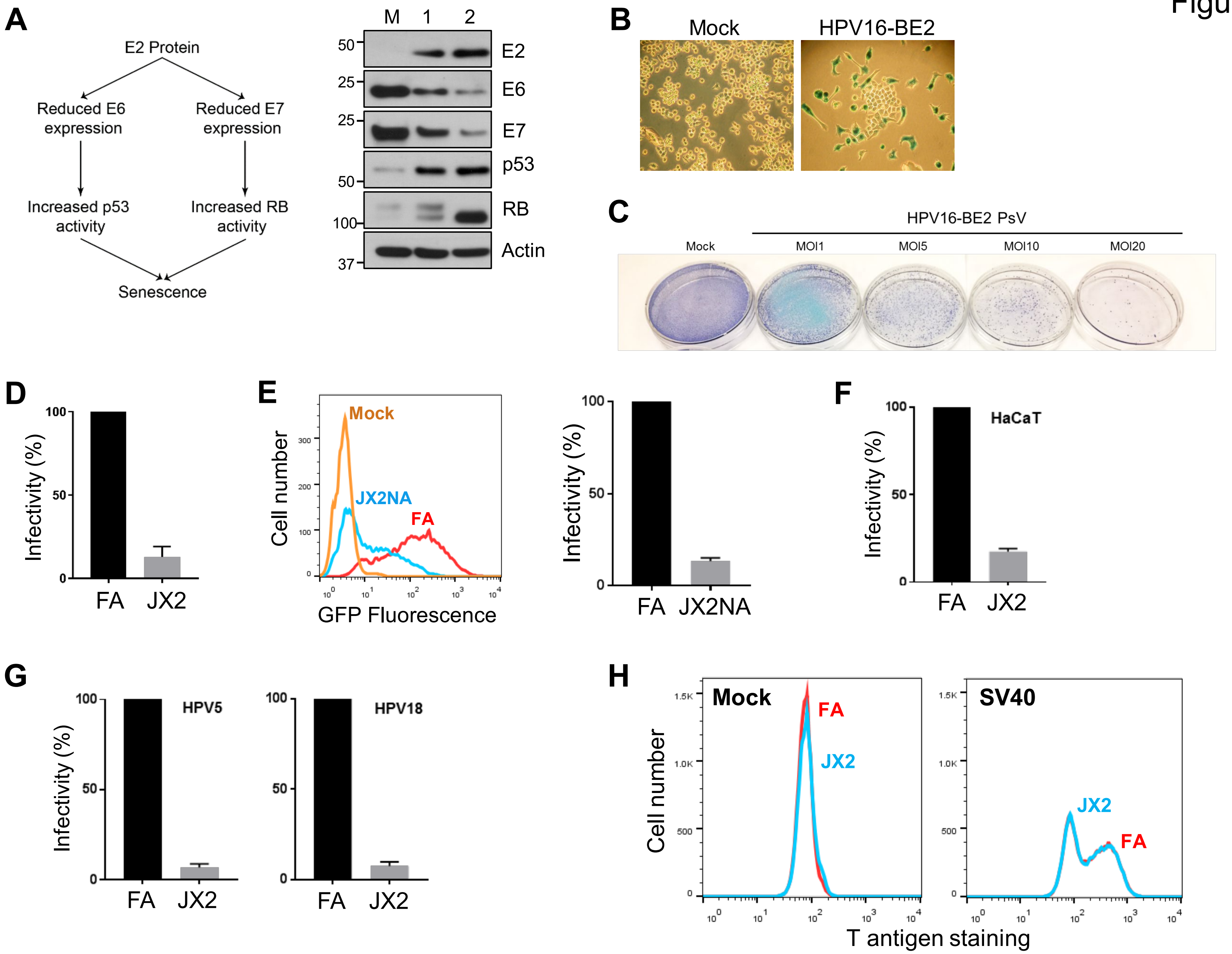

Related to Figure 1

Figure S1. Related to Figure 1. Isolation and validation of a traptamer that inhibits HPV infection. (A) Left panel. Schematic of biochemical pathway leading to senescence following E2 expression. Right panel. Extracts were prepared from mock-infected HeLa S3 cells (M) and HeLa S3 cells one and two days after infection with HPV16-BE2 at MOI of 20. Samples were subjected to SDS-PAGE and immunoblotted with antibodies recognizing BPV E2, HPV18 E6, HPV18 E7, p53, $p105^{Rb}$, and actin as loading control. **(B)** Photomicrographs of mock-infected HeLa S3 cells (left panel) and HeLa S3 cells 14 days after infection with HPV16-BE2 at MOI of 5 (right panel) stained for senescence-associated β-galactosidase. **(C)** HeLa S3 cells were mock-infected or infected with HPV16-BE2 at the indicated multiplicities of infection (MOI), plated at low cell density, incubated for two weeks, and stained with Giemsa. **(D)** Clonal HeLa-tTA cells expressing pT-FA or pT-JX2 in the absence of doxycycline were infected with HPV16-GFP PsV at MOI of 2, and the fraction of cells expressing GFP two days later was quantified by flow cytometry as a measure of infectivity. Graph shows mean and standard deviation of three independent experiments relative to cells expressing FA, which was set at 100%. **(E)** Clonal HeLa-tTA cells expressing pT-FA or pT-JX2NA, which lacks the APEX2 segment, were mock-infected or infected with HPV16-GFP PsV in the absence of doxycycline and analyzed as in **panel D**. Graph on left shows flow cytometry histograms. Averaged results of three independent experiments are shown at right. **(F)** HaCaT keratinocytes expressing vector control or JX2 were infected and analyzed as in **panel D**. **(G)** Clonal HeLa-tTA cells expressing pT-FA or pT-JX2 in the absence of doxycycline were infected with HPV5 and HPV18 PsV, as indicated, at MOI of 2 and analyzed as in **panel D. (H)** Clonal HeLa-tTA cells expressing pT-FA or pT-JX2 in the absence of doxycycline were mock-infected (left panel) or infected with SV40 at MOI of 1 (right panel). Two days later, cells were stained with antibody recognizing SV40 large T antigen and analyzed by flow cytometry.

Figure S2

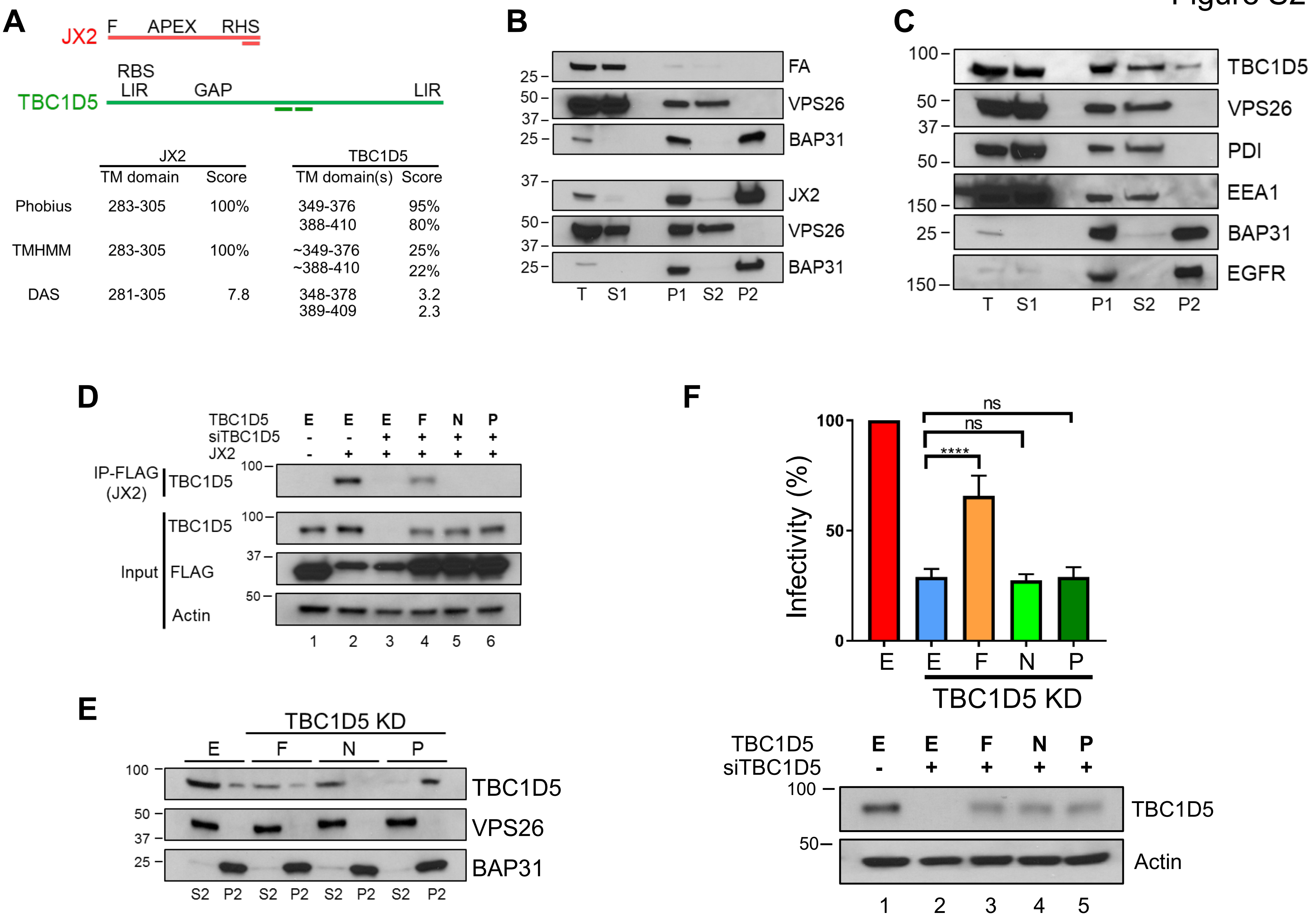

Related to Figures 1 and 4

Figure S2. Related to Figures 1 and 4. JX2 is a transmembrane protein. (A) Schematic diagram of JX2 (red) and TBC1D5 (green) with domains indicated: F, FLAG epitope; APEX, APEX2 segment; RHS, randomized hydrophobic segment; RBS, retromer binding site; GAP, GTPase activating segment; LIR, LC3 interacting region. Short lines represent putative TM domains. Table at bottom shows TM domains predicted by Phobius, TMHMM, or DAS prediction programs. The numbers in the TM domain columns give the boundaries of the predicted TM domain(s). The score indicates the probability that these sequences adopt a TM existence or the DAS score, where numbers higher than 2.2 reflect a high confidence of adopting a TM existence. **(B)** Clonal HeLa-tTA cells expressing pT-FA (top panels) or pT-JX2 (bottom panels) were osmotically swollen, mechanically sheared, fractionated into soluble and membrane fractions, and extracted with carbonate. Fractions were subjected to SDS-PAGE and immunoblotted for FLAG (recognizing FA and JX2), VPS26 (a peripheral membrane protein), and BAP31 (a TM protein). T, total extract; S1, supernatant containing soluble proteins; P1, total membrane pellet; S2, supernatant containing carbonate-extracted luminal and peripheral membrane proteins; P2, carbonate-resistant integral membrane protein pellet. **(C)** Lysates of uninfected HeLa S3 cells were prepared and processed as in **panel B**. Fractions were also immunoblotted for TBC1D5; the peripheral membrane protein, EEA1; the luminal protein, protein disulfide isomerase (PDI); and the TM protein, EGF receptor. **(D)** Clonal HeLa-tTA cells expressing JX2 (+) or FA (-) in the absence of doxycycline were transduced to express full-length TBC1D5 (F), the TBC1D5 null mutant (N) or the TBC1D5 PRTM mutant (P) or expressed only endogenous TBC1D5 (E). Cells were then transfected with scrambled control siRNA (-) or siRNA targeting the 3' untranslated region of the *TBC1D5* gene (+). Forty-eight hours later, extracts were prepared and expression of TBC1D5 was determined by immunoblotting (Input) or were analyzed by co-immunoprecipitation for JX2 binding as in Figure 3B. **(E)** Clonal HeLa-tTA cells expressing endogenous TBC1D5 or exogenous TBC1D5 and knocked down for endogenous expression as in **panel D** were subjected to carbonate extraction as in **panel B**. Only P2 and S2 fractions are shown. **(F)** Clonal HeLa-tTA cells as in **panel D** were infected 48 h after siRNA transfection with HPV16-GFP PsV at MOI of 2, and GFP fluorescence was measured 48 h.p.i. Infectivity was normalized to cells expressing endogenous TBC1D5. Bottom panels show immunoblot of TBC1D5 and actin expression.

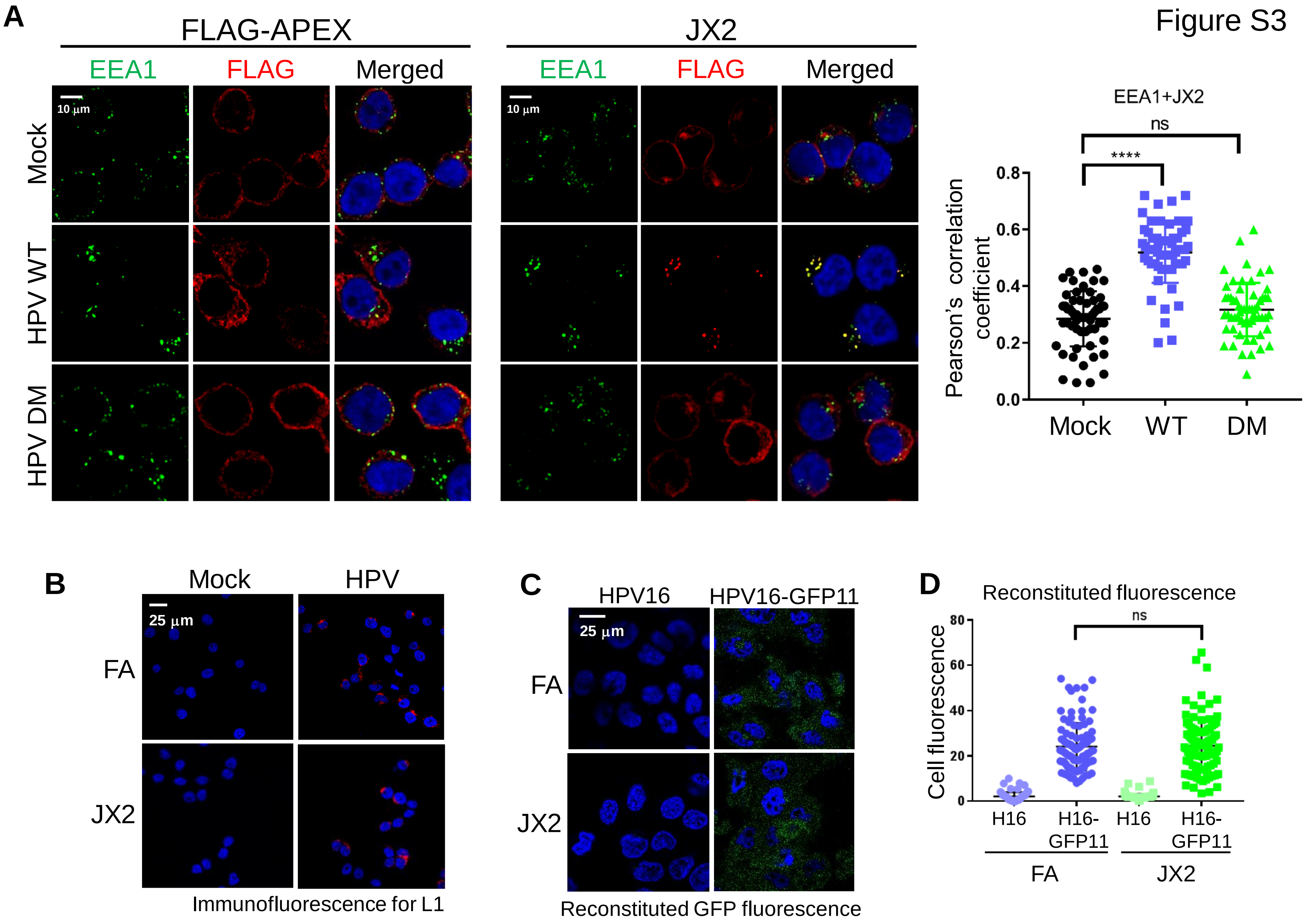

Related to Figures 2 and 5

Figure S3. Related to Figures 2 and 5. JX2 does not inhibit HPV internalization or cytoplasmic protrusion of L2. (A) HeLa-tTA cells expressing pT-FA or pT-JX2 in the absence of doxycycline were mock-infected or infected with wild-type HPV16-HcRed PsV at MOI of 150 or with DM HPV16-HcRed PsV containing the same number of encapsidated HcRed reporter plasmids. At 12 h.p.i., cells were stained with antibodies recognizing EEA1 (green) and FLAG-tagged JX2 (red) and visualized by confocal microscopy. Co-localization of EEA1 and JX2 is pseudocolored yellow in the Merged panels. A single confocal Z-plane is shown in each image. Graph shows Pearson's Coefficient for co-staining, with each dot representing an individual cell (at least 60 cells for each condition). ns, not significant; ****, $p<0.0001$. **(B)** Clonal HeLa-tTA cells expressing pT-FA or pT-JX2 in the absence of doxycycline were mock-infected or infected with HPV16 PsV at MOI of 50. At 8 h.p.i., cells were permeabilized and stained with antibody recognizing HPV16 L1 (red) and DAPI to stain nuclei (blue). **(C)** Clonal HaCaT-GFP1-10NES cells expressing pT-JX2 (bottom panels) or pT-FA (top panels) were infected at MOI of 2,000 with HPV16-HcRed PsV containing wild-type L2 (left panels) or L2 containing GFP11 fused to its C terminus (HPV16-GFP11) (right panels). Three h.p.i., cells were examined by confocal microscopy to detect reconstituted GFP fluorescence as a measure of cytoplasmic protrusion of L2. Fluorescence, green; nuclei, blue. **(D)** Fluorescence of cells as in **panel C** (at least 100 cells for each condition) was quantitated and shown as mean and standard deviation. H16, HPV16 PsV; H16-GFP11, HPV16-GFP11. Each dot represents an individual cell. ns, not significant.

Figure S4

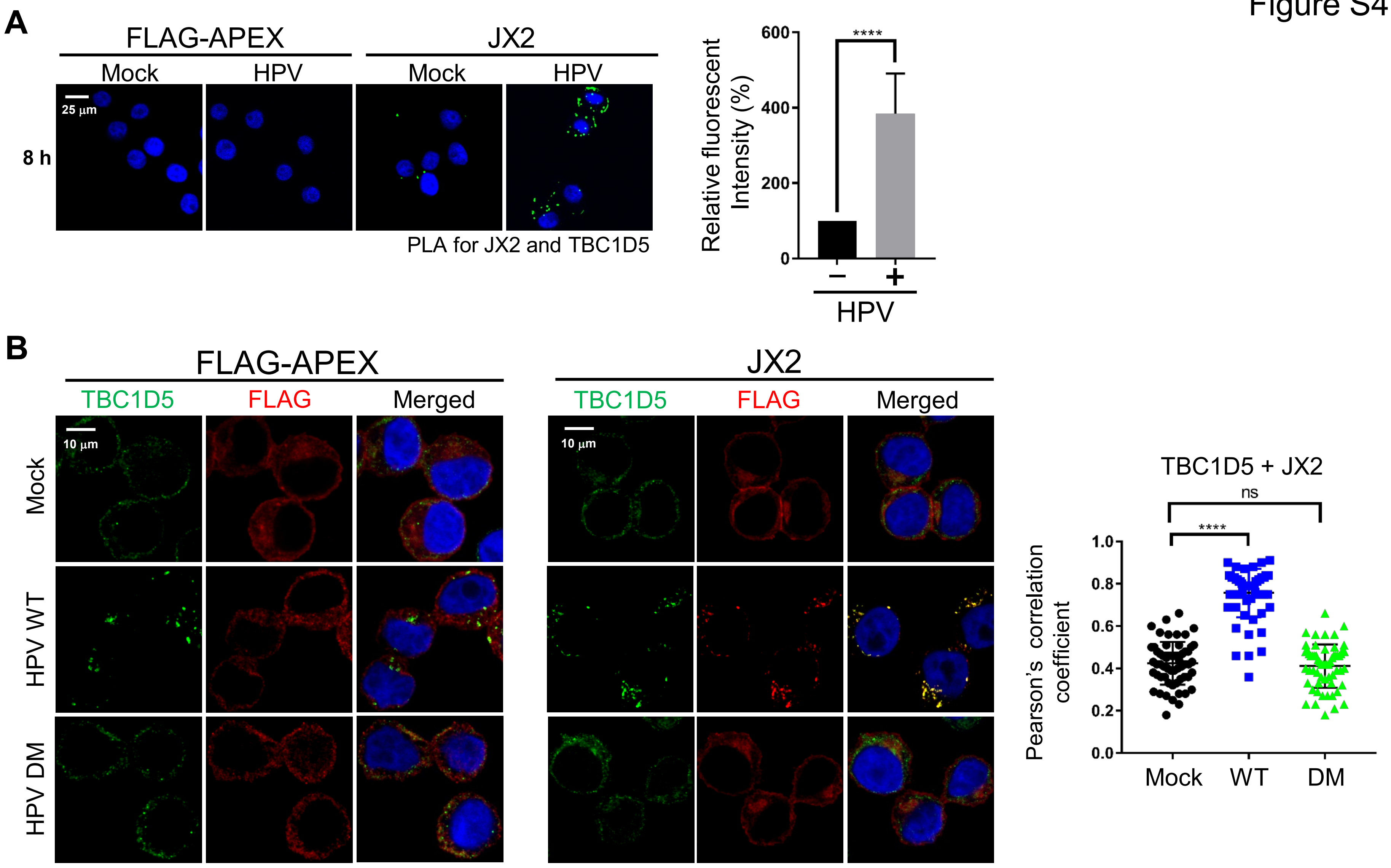

Related to Figures 2 and 5

Figure S4. Related to Figures 2 and 5. HPV infection induces co-localization of TBC1D5 and JX2. (A) Left panels. Clonal HeLa-tTA cells expressing pT-FA or pT-JX2 in the absence of doxycycline were mock-infected or infected with HPV16-HcRed PsV at MOI of 150. Eight h.p.i., cells were processed for PLA with antibodies recognizing TBC1D5 and FLAG-tagged JX2. PLA signal is green; nuclei are stained blue. Right panel. JX2-TBC1D5 PLA signal in at least 200 cells for each condition expressing JX2 with and without HPV16 infection was quantified. Results show mean signal and standard deviation normalized to mock-infected cells, which were set to 100%. ****, $p<0.0001$. **(B)** Cells as in **panel A** were mock-infected or infected with wild-type HPV16-HcRed PsV at MOI of 150 or DM mutant PsV containing the same number of encapsidated HcRed reporter plasmids. Twelve h.p.i., cells were stained with antibody recognizing TBC1D5 (green) or FLAG (red) and visualized by confocal microscopy. Overlap between TBC1D5 and FLAG is pseudocolored yellow in the Merged panels. Nuclei are stained blue. A single confocal Z-plane is shown in all images. Graph shows Pearson's correlation co-efficient of the overlap for at least 60 cells for each condition, displayed as in Figure 5B. ns, not significant; ****, $p<0.0001$.

Figure S5

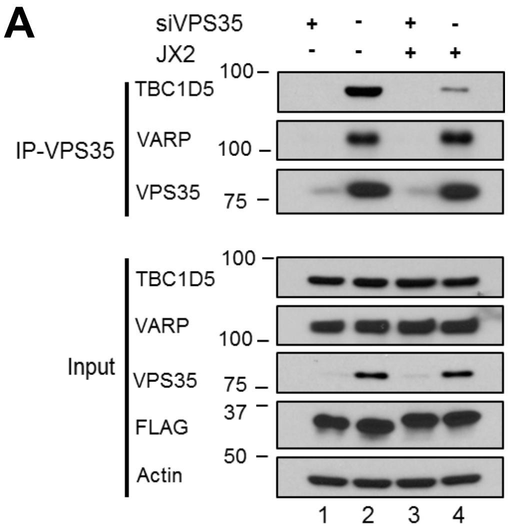

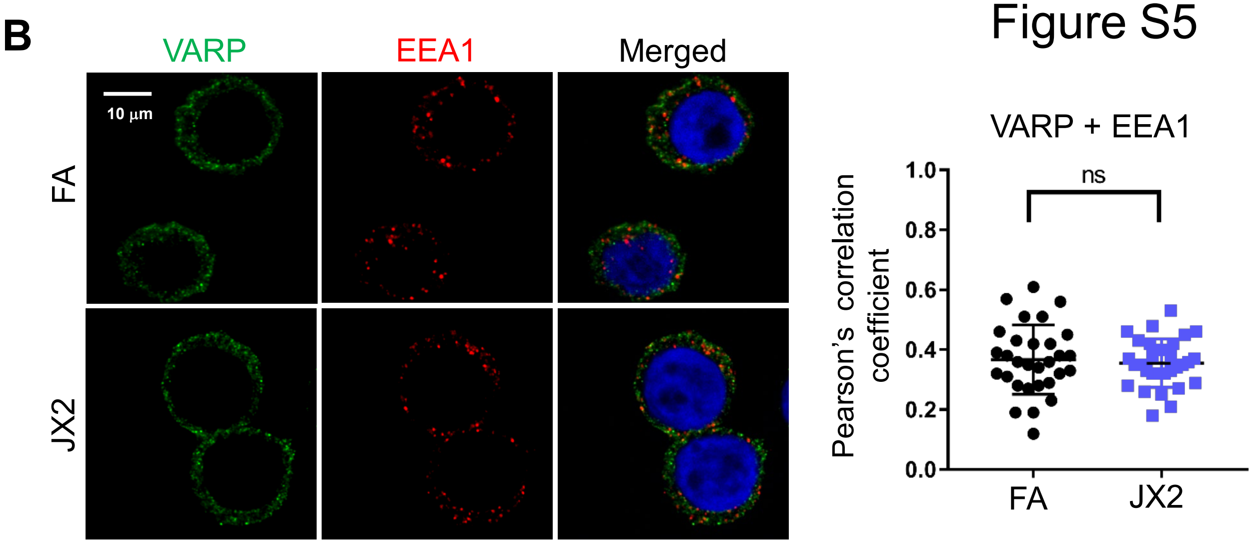

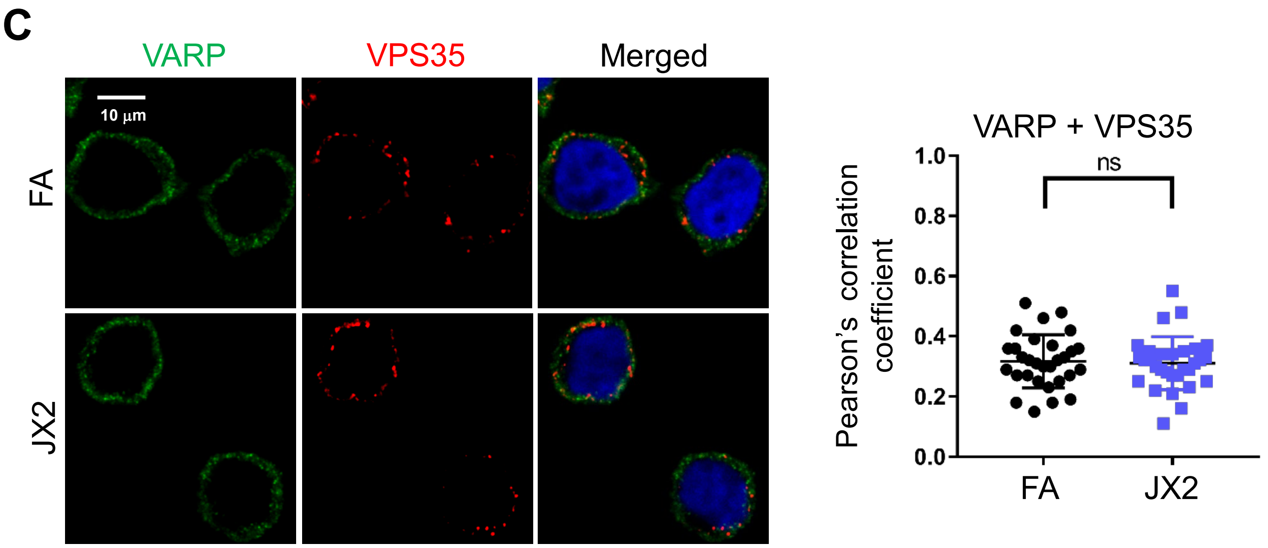

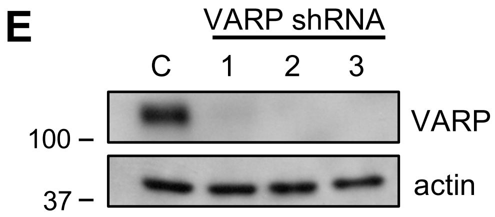

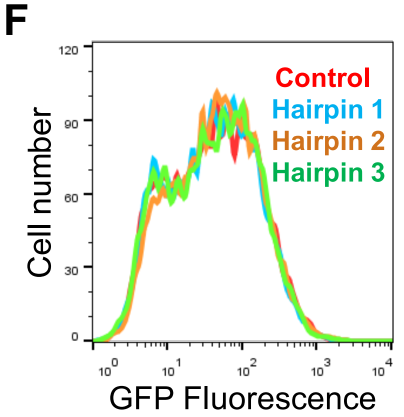

D

Hairpin 1: CCAAGCTGTGTTGCTTCATTA
Hairpin 2: CCGGAGTTCAGCTTTAACATA
Hairpin 3: GATCCCTAATTGGATGGCAAA

Related to Figures 3 and 4

Figure S5. Related to Figures 3 and 4. Lack of role of VARP in JX2 activity or HPV infection. (A) HeLa-tTA cells expressing pT-FA (-) or pT-JX2 (+) in the absence of doxycycline were transfected with control siRNA (-) or siRNA targeting VPS35 (+). Extracts were prepared two days after transfection and immunoprecipitated with anti-VPS35, electrophoresed, and immunoblotted for the indicated proteins. Input shows samples without immunoprecipitation. **(B and C)** HeLa-tTA cells expressing pT-FA or pT-JX2 in the absence of doxycycline were stained with antibody recognizing VARP (green) and either EEA1 (**panel B**) or VPS35 (**panel C**) (red). Co-localization is pseudocolored yellow in Merged panels, which is quantitated in the graphs. **(D)** Targeting sequences of shRNAs inhibiting VARP expression. **(E)** Western blot showing knockdown of VARP expression by the indicated shRNAs. C, controlled scrambled shRNA. **(F)** Flow cytometry histogram of cells expressing the indicated shRNAs and infected with HPV16-GFP PsV at MOI of 2. GFP fluorescence was measured 48 h.p.i. ns, not significant.

Figure S6

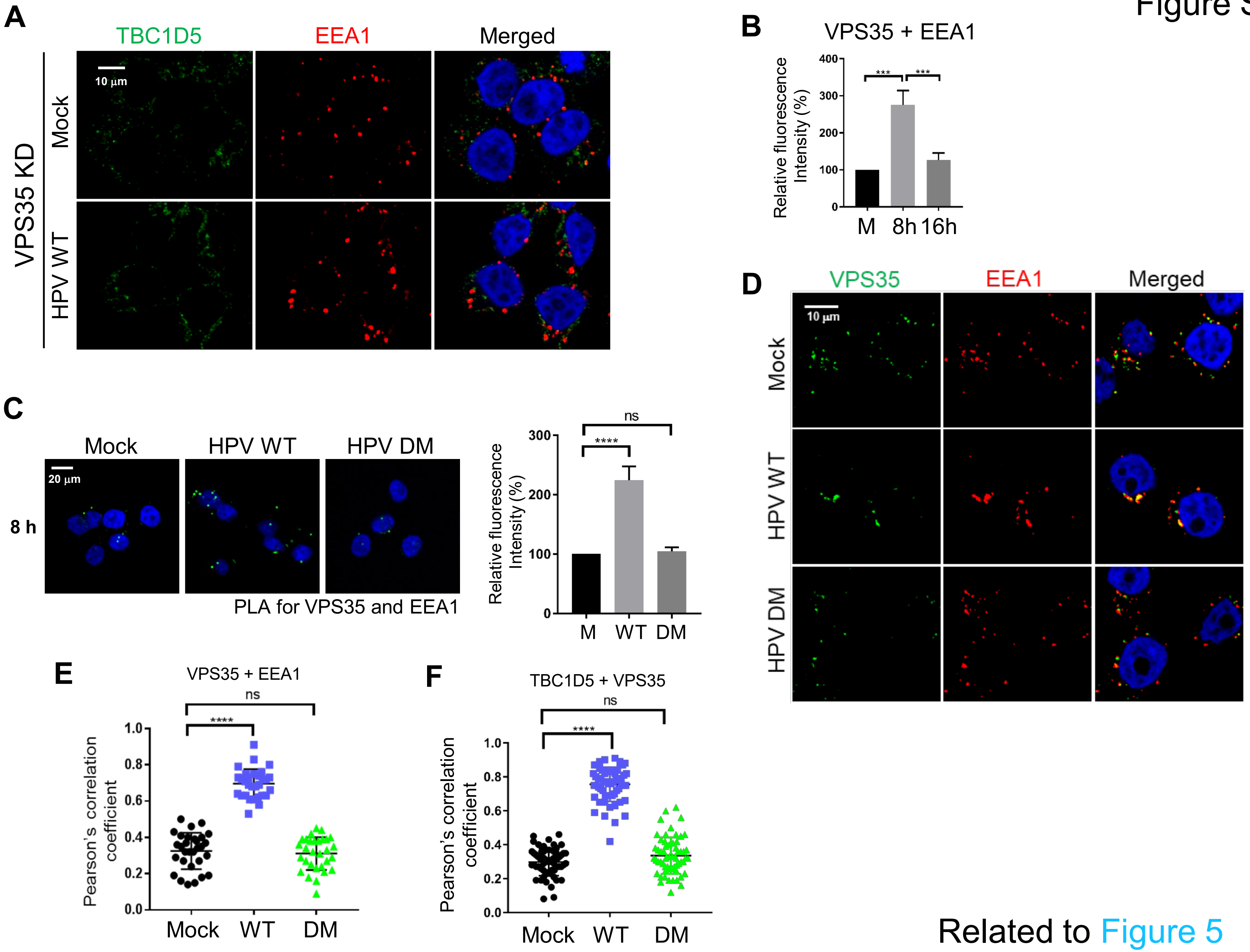

Related to Figure 5

Figure S6. Related to Figure 5. Effect of HPV infection on distribution of VPS35, EEA1, and TBC1D5. (A) See legend to Figure 5A. **(B)** VPS35 and EEA1 PLA signal as in Figure 5C (left panels) at 8 and 16 h.p.i were quantified as described in the legend to Figure 2B. ***, $p<0.001$. **(C)** HeLa S3 cells were mock-infected or infected with wild-type HPV16-HcRed PsV at MOI of 150 or DM mutant PsV containing the same number of encapsidated HcRed reporter plasmids. At 8 h.p.i., cells were subjected to PLA with antibodies recognizing VPS35 and EEA1. PLA signal is green, and nuclei are blue. VPS35-EEA1 PLA signal was quantified and displayed as described in legend to Fig. 2B. **(D)** HeLa S3 cells were infected as in **panel C**. At 12 h.p.i., cells were stained with antibodies recognizing VPS35 (green) and EEA1 (red) and visualized by confocal microscopy. Overlap between VPS35 and EEA1 is pseudocolored yellow in Merged panel. Nuclei are stained blue. **(E)** VPS35-EEA1 overlap in images as in **panel D** was quantified as described in Figure 5B. **(F)** TBC1D5-VPS35 overlap in images as in Figure 5D was quantified as described in Figure 5B, for at least 60 cells for each condition. ns, not significant; ****, $p<0.0001$.

Figure S7

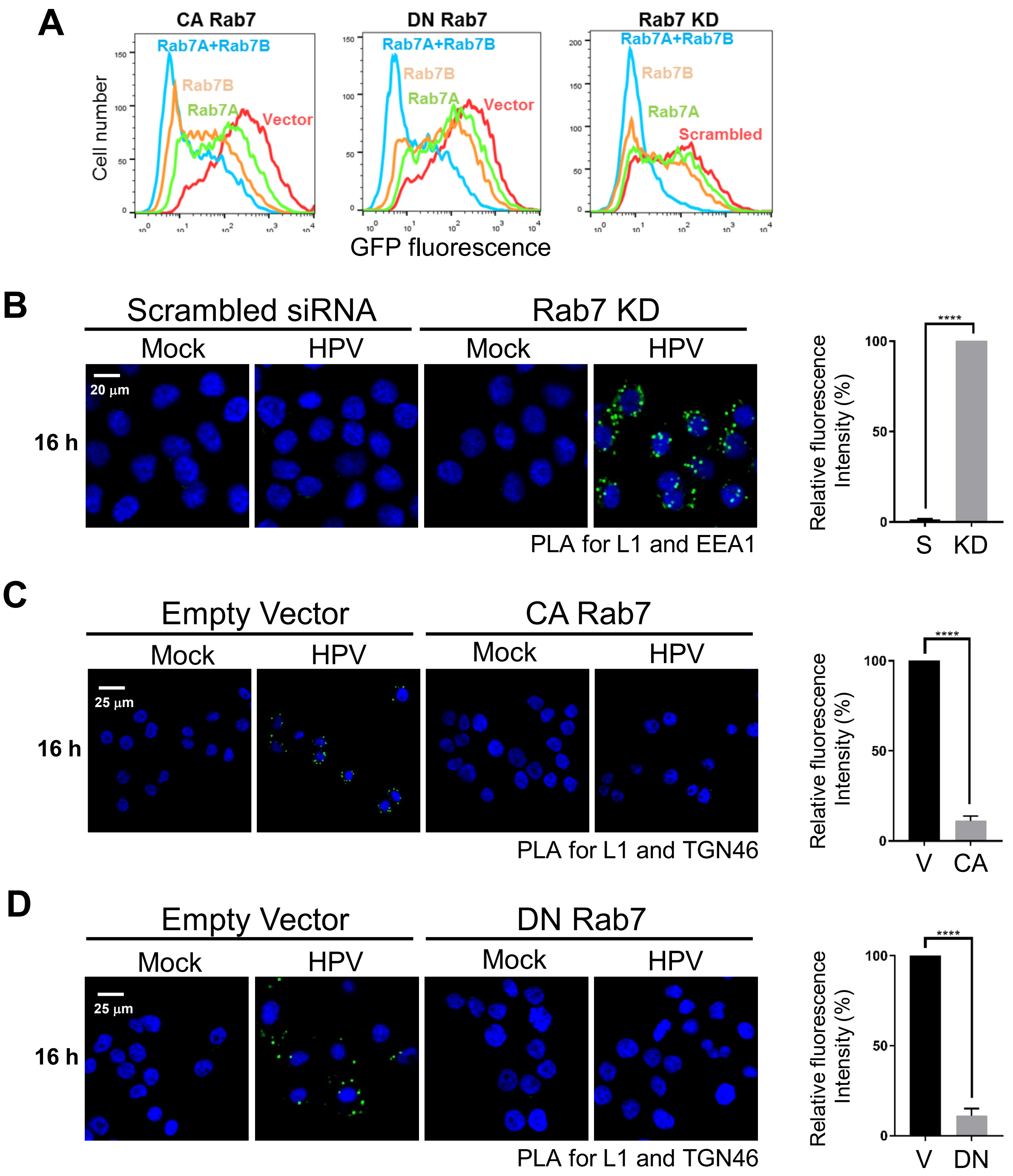

Related to Figure 6

Figure S7. Related to Figure 6. Rab7 cycling is required for HPV infection and transit to the trans-Golgi network. (A) HeLa-tTA cells as in Figure 6B were infected with HPV16-GFP PsV at MOI of 2. Two days after infection, GFP fluorescence was measured by flow cytometry. Graphs show representative flow cytometry histograms. **(B)** Left panels. HeLa S3 cells were transfected with control scrambled siRNA or siRNAs targeting Rab7A and Rab7B. Two days later, cells were mock-infected or infected with HPV16-HcRed PsV at MOI of 150. Sixteen h.p.i., cells were subjected to PLA with antibodies recognizing HPV L1 and EEA1. PLA signal is green; nuclei are stained blue. Right panel. L1-EEA1 PLA signal in at least 200 cells for each HPV-infected condition was quantified and displayed as described in legend to Figure 2B. S, control scrambled siRNA; KD, Rab7A and Rab7B siRNA. ****, p<0.0001. **(C)** Left panels. Clonal HeLa-tTA cells expressing pT_{puro} and pT_{hygro} (Empty Vector) or expressing CA Rab7A plus Rab7B in the absence of doxycycline were mock-infected or infected with HPV16-HcRed PsV at MOI of 150. Sixteen h.p.i., cells were subjected to PLA with antibodies recognizing HPV L1 and TGN46. PLA signal is green; nuclei are stained blue. Right panel. L1-TGN46 PLA signal in at least 200 cells for each HPV-infected condition was quantified and displayed as described in legend to Figure 2B. V, empty vector; CA, constitutively-active Rab7A and Rab7B. ****, p<0.0001. **(D)** Left panels. As in **panel C**, except cells expressed DN Rab7A plus Rab7B instead of CA Rab7. Right panel. L1-TGN46 PLA signal in at least 200 cells for each HPV-infected condition was quantified and displayed as described in legend to Figure 2B. V, empty vector; DN, dominant-negative Rab7A and Rab7B. ****, p<0.0001.

Table S1. Related to Figures 4, 6, 7, and S7. Summary of the Effect of TBC1D5 and Rab7 on Retromer-mediated Cargo Trafficking

	infectivity HPV	endosome entry		endosome accumulation		TGN entry		retromer association	retromer dissociation
		HPV	cell cargo	HPV	cell cargo	HPV	cell cargo	HPV	HPV
wild-type cells	+	+	+	-	-	+	+	+	+
TBC1D5 knockdown	-	+	+	+	-	-	+	+	-
CA Rab7	-	+	+	+	-	-	+	+	-
DN Rab7	-	+	+	+	+	-	-	-	n.a.

n.a. – not applicable

Table S2. Related to STAR METHODS. Oligonucleotides for Library Construction and Recovery

FWD long	GCCTGCTAGGGATCCGGCGGC N5B N5B N5B N5B N5B N5B N5B N5B N5B N5B N5B N5B N5B N5B N5B N5B N5B N5B N5B N5B N5B N5B N5B N5B N5B N5B N5B N5B N5B N5B TAG TAG CCT GAT GAG GAA<br><br>N is A:G:C:T = 1:1:1:1<br>5 is A:G:C:T = 1:1:1:7<br>B is A:G:C:T = 0:1:1:1
REV Long	GAATTCTCCCTACACTGCTCACTCATCAGGCTACTA
FWD short	GGCTCTTTTGTCTGACCCTGTATTCCGCCC
REV short	GACACT GAATTC TCC CTA CAC TGC TCA CTC

Table S3. Related to STAR METHODS. Antibodies Used for Proximity Ligation Assays

Antibody 1 (Mouse)		Antibody 2 (Rabbit)	
anti-HPV16 L1	BD Biosciences 554171; 1:75[a]	anti-EEA1<br>anti-TGN46<br>anti-VSP35	Cell Signaling Technologies 2411; 1:50<br>Abcam ab50595; 1:150<br>Abcam ab157220; 1:75
anti-FLAG (recognizes JX2)	Sigma F3165; 1:200	anti-TBC1D5	Abcam ab203896; 1:50
EEA1	BD Biosciences 610457; 1:75	anti-VPS35	as above

[a] Dilutions used are indicated for all antibodies.
